# Strain‐Release Driven Epoxidation and Aziridination of Bicyclo[1.1.0]butanes via Palladium Catalyzed σ‐Bond Nucleopalladation

**DOI:** 10.1002/ange.202217064

**Published:** 2023-01-12

**Authors:** Bernhard Wölfl, Nils Winter, Jiajing Li, Adam Noble, Varinder K. Aggarwal

**Affiliations:** ^1^ School of Chemistry University of Bristol Cantock's Close Bristol BS8 1TS UK

**Keywords:** Aziridines, Bicyclo[1.1.0]Butanes, Epoxides, Spiro Compounds, Strained Molecules

## Abstract

The development of preparative methods for the synthesis of four‐membered carbocycles is gaining increasing importance due to the widespread utility of cyclic compounds in medicinal chemistry. Herein, we report the development of a new methodology for the production of spirocyclic epoxides and aziridines containing a cyclobutane motif. In a two‐step one‐pot process, a bicyclo[1.1.0]butyl sulfoxide is lithiated and added to a ketone, aldehyde or imine, and the resulting intermediate is cross‐coupled with an aryl triflate through C−C σ‐bond alkoxy‐ or aminopalladation with concomitant epoxide or aziridine formation. After careful optimization, a remarkably efficient reaction was conceived that tolerated a broad variety of both aromatic and aliphatic substrates. Lastly, through several high yielding ring‐opening reactions, we demonstrated the excellent applicability of the products as modular building blocks for the introduction of three‐dimensional structures into target molecules.

## Graphical abstract

Strained spirocyclic epoxides and aziridines were synthesized via three‐component coupling between in situ generated 1‐lithio bicyclo [1.1.0]butane, a broad variety of ketones, aldehydes and *N*‐tosyl imines, and aryl triflates. The key step involves a highly diastereoselective palladium‐catalyzed C−C σ‐bond alkoxyarylation (or aminoarylation) of bicyclo [1.1.0]butyl carbinolate intermediates.

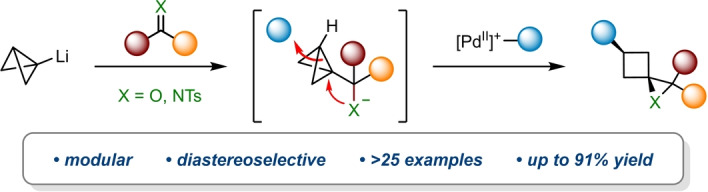

## Introduction

**Scheme 1:**
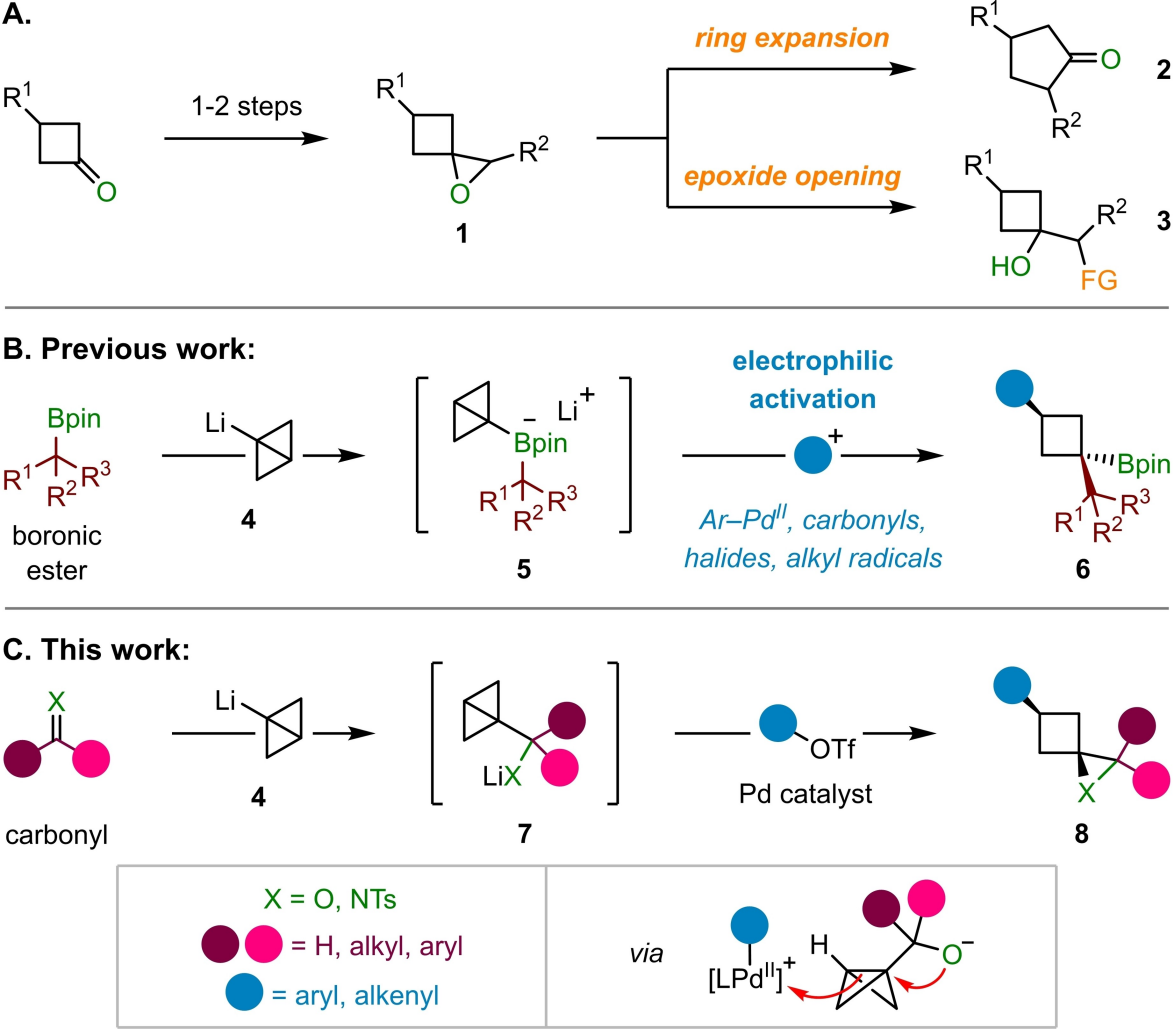
A) Synthesis and applications of 1‐oxaspiro[2.3]hexanes. B) Modular synthesis of 1,1,3‐trisubstituted cyclobutanes by electrophilic activation of bicyclo[1.1.0]butyl boronates. C) Strain‐release driven spirocyclization approach to 1‐oxaspiro[2.3]hexanes and 1‐azaspiro[2.3]hexanes from bicyclo[1.1.0]butyl carbinolates.

Spirocyclic epoxides belonging to the class of 1‐oxaspiro[2.3]hexanes **1** are highly versatile compounds for organic synthesis (Scheme 1A).^[1]^ Their intrinsic strain renders these structures prone to ring‐expansion,^[2]^ which has been extensively exploited for the synthesis of cyclopentanones **2**.^[3]^ An alternative application of these epoxides, which is becoming increasingly prominent, is the formation of hydroxy‐substituted cyclobutanes **3** through ring‐opening reactions with nucleophiles, offering a convenient method to introduce cyclobutane motifs into target molecules.^[4]^ The incorporation of such structures into drug‐molecules can potentially improve, alter, or modulate both physicochemical and pharmaco‐kinetic properties.^[5]^ Moreover these three‐dimensional motifs provide a gateway beyond the flat (two‐dimensional) structures that have long dominated small molecule screening libraries.^[6]^

1‐Oxaspiro[2.3]hexanes **1** are commonly obtained from cyclobutanones, either by reaction with a carbenoid (e.g., sulfur ylides),[^1^, ^7^] or via olefination and subsequent epoxidation[^1^, ^8^] of the resulting methylene cyclobutane.^[9]^ However, for the introduction of different functional groups onto the cyclobutane ring, these strategies require this functionality to be pre‐installed in the cyclobutanone starting material. Due to the rising importance of four‐membered carbo‐ and heterocycles in medicinal chemistry, there is an increasing demand for new, efficient, rapid, and modular synthetic methods for their construction.^[10]^ We recently reported a modular synthesis of 1,1,3‐trisubstituted cyclobutanes **6** through strain‐release driven reactions of bicyclo[1.1.0]butyl (BCB) boronates **5** formed from boronic esters and BCB lithium **4** (Scheme 1B).[^11^, ^12^] These reactions proceed via activation of the strained central C−C σ‐bond of the BCB group of **5** with various electrophiles, which triggers a 1,2‐metallate rearrangement to generate cyclobutyl boronic esters **6**. We reasoned that a related electrophile‐induced ring‐opening of BCB carbinolates **7**, accessed by addition of **4** to a carbonyl, could provide modular access to 1‐oxaspiro[2.3]hexanes **8** (X=O) in a strain‐release spirocyclization (Scheme 1C).[^13^, ^14^] Herein, we report the successful realization of this strategy by reacting ketone and aldehyde‐derived BCB‐carbinolates **7** with aryl triflates in a C−C σ‐bond alkoxypalladation. Two C−C bonds and one C−O bond are generated in a single operation and the reactions proceed with excellent diastereoselectivity over a wide range of substrates. In addition, *N*‐tosyl imines could be employed to access spirocyclic aziridines via an analogous aminopalladation, thus showcasing the potential of strain‐release driven C−C σ‐bond nucleopalladations for accessing diverse spirocycles.

## Results and Discussion

**Scheme 2:**
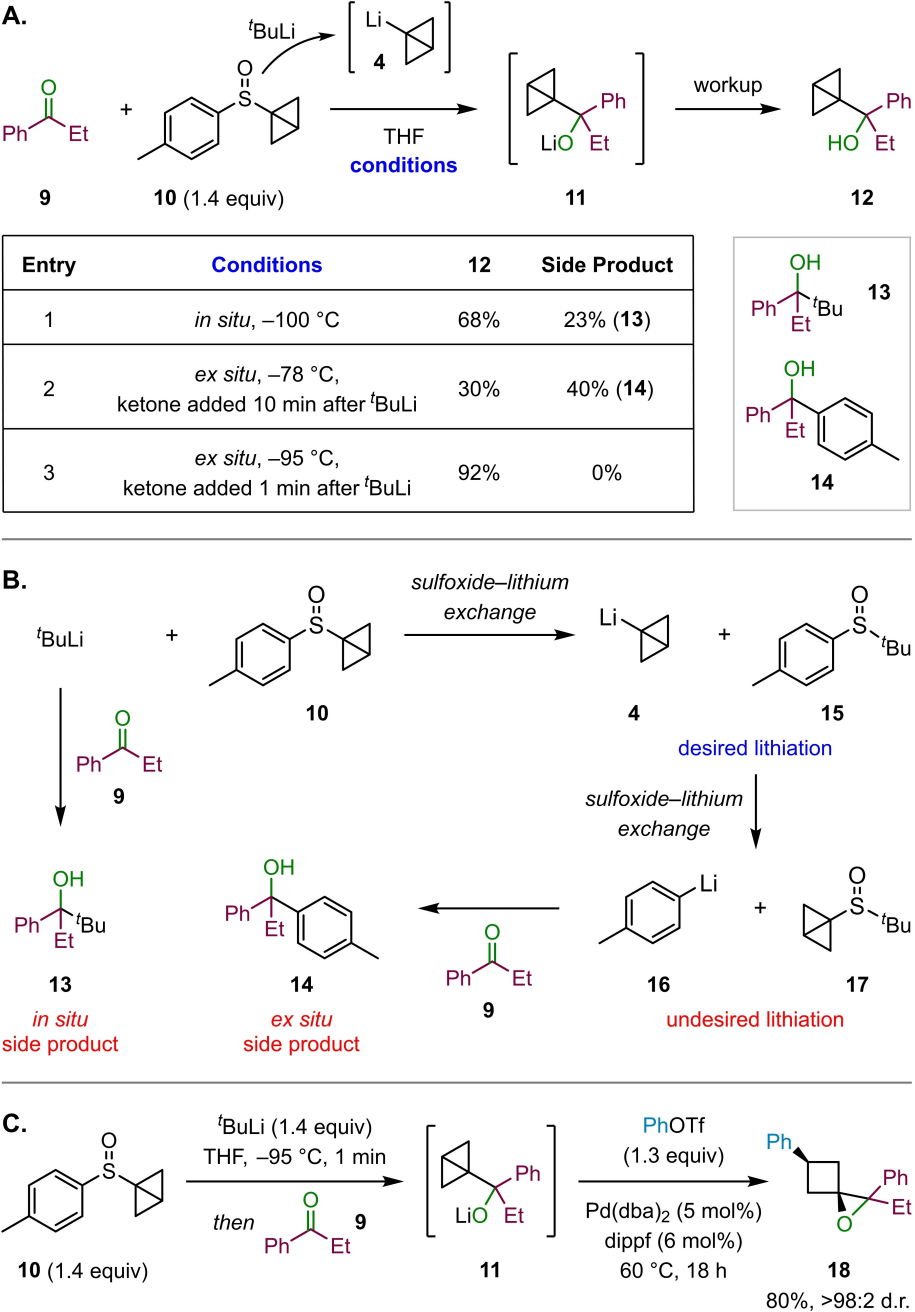
A) Optimization of BCB carbinolate formation; yields determined by ^1^H NMR analysis. B) Formation of side products during BCB carbinolate formation. C) Palladium‐catalyzed cross‐coupling for the formation of 1‐oxaspiro[2.3]hexanes; yield of isolated product.

At the outset of this project we envisioned designing a practical and efficient synthetic methodology that would allow us to generate a broad variety of 1‐oxaspiro[2.3]hexane derivatives. Therefore, the first stage of our investigation was to identify reliable conditions for the formation of the key BCB‐carbinolates **7** from carbonyls and BCB‐Li **4**. In our previously reported reactions of BCB‐boronates, we demonstrated that **4** could be conveniently generated in situ from BCB sulfoxide **10** by sulfoxide‐lithium exchange.[^11a^, ^11b^, ^11c^] Sulfoxide **10** represents an excellent gateway to the formation of cyclobutane derivatives as it is a bench stable white solid, which can be readily made in two steps from commercially available substances. We initially investigated the formation of BCB carbinolate **11** through in situ lithiation of sulfoxide **10** with *tert*‐butyllithium in the presence of propiophenone (**9**) (Scheme 2A, entry 1). Unfortunately, these conditions proved problematic due to the formation of side product **13**, derived from the reaction of ketone **9** with *tert*‐butyllithium. Even at temperatures as low as −100 °C (ethanol/liquid nitrogen), a significant amount of **13** was observed along with the expected BCB carbinol **12**. In an attempt to avoid this side reaction, a stepwise process was pursued consisting of ex situ formation of BCB‐Li **4** by addition of *tert*‐butyllithium to sulfoxide **10** at −78 °C, thus ensuring that *tert‐*butyllithium would be completely consumed before addition of ketone **9** after 10 min. Contrary to our expectations, following this ex situ lithiation procedure, large quantities of a different side product **14** were observed (Scheme 2A, Entry 2). This unexpected side product originated from the reaction of ketone **9** with *p*‐tolyllithium (**16**) (Scheme 2B). As this product was exclusively observed under ex situ conditions in the absence of ketone **9**, we propose that 1‐lithio bicyclo[1.1.0]butane (**4**), which is the kinetic product of the initial sulfoxide‐lithium exchange reaction, undergoes a second sulfoxide–lithium exchange with sulfoxide byproduct **15** to form **16** and dialkyl sulfoxide **17** as the thermodynamic products (Scheme 2B).^[15]^ This could be remedied by both reducing the time interval between the addition of *tert‐*butyllithium and ketone **9** to just 1 min, as well as shortening the addition time of the reagents to 1 min. Additionally, the reaction temperature was lowered to −95 °C (acetone/liquid nitrogen). Indeed, after implementing all these modifications, the interfering side products were no longer observed, allowing for a reproducible procedure towards BCB carbinolate **11** (Scheme 2A, Entry 3).

For the subsequent BCB ring‐opening/spirocyclization step to generate 1‐oxaspiro[2.3]hexane **18**, we investigated the palladium‐catalyzed cross‐coupling of BCB carbinolate **11** with phenyl triflate (Scheme 2C).^[11a]^ We were delighted to find that simple addition of phenyl triflate and a solution of bis(dibenzylideneacetone)palladium(0) (Pd(dba)_2_) and 1,1′‐bis(diisopropylphosphino)ferrocene (dippf) to the solution of **11**, followed by heating at 60 °C for 18 h, provided 1‐oxaspiro[2.3]hexane **18** in excellent yield and as a single diastereomer.

**Scheme 3:**
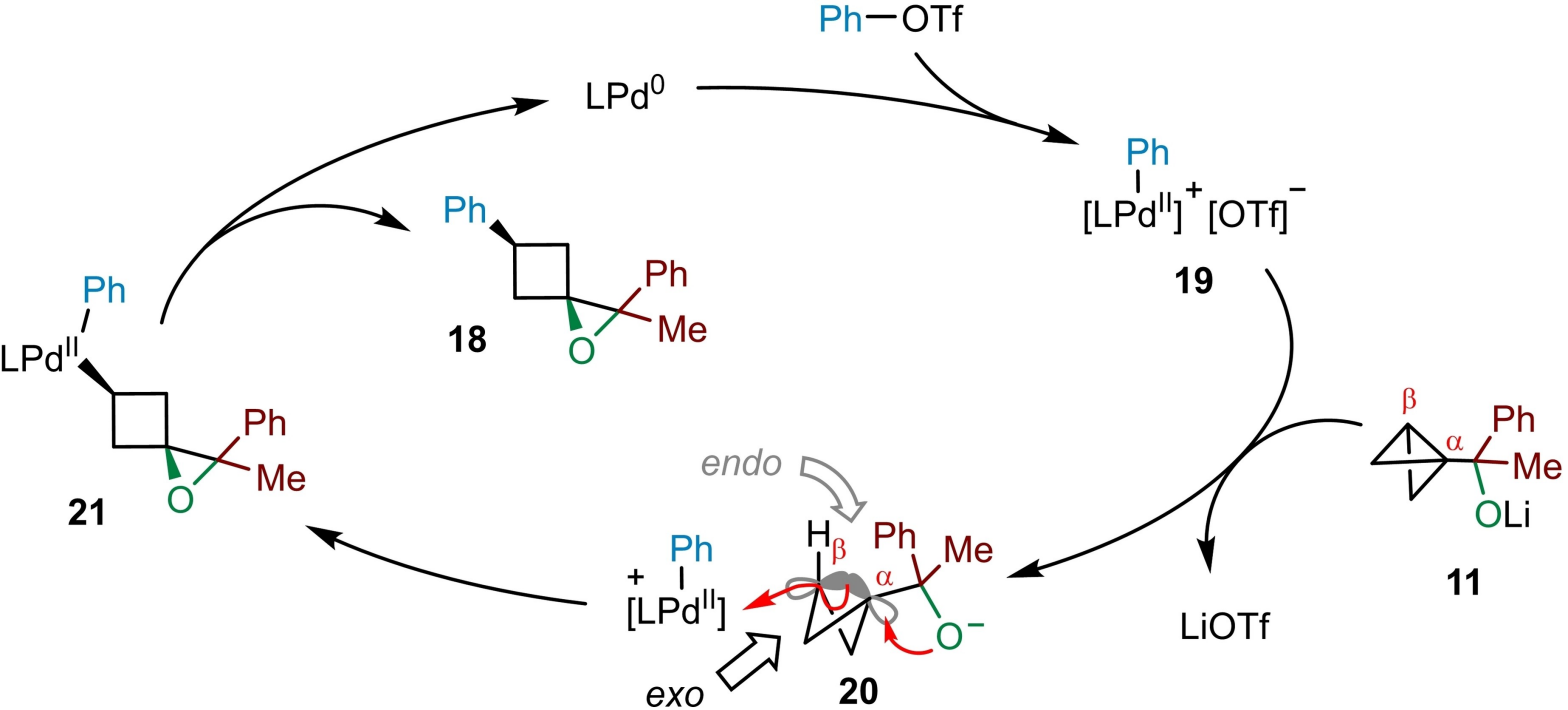
Proposed catalytic cycle for palladium‐catalyzed spirocyclization.

Based on our previous studies on BCB‐boronate complexes,[^11a^, ^11b^] we propose the catalytic cycle shown in Scheme 3 for the palladium‐catalyzed spirocyclization of BCB carbinolate **11**. The mechanism commences with an oxidative addition of phenyl triflate to the Pd^0^ catalyst to give electrophilic aryl palladium (II) complex **19**, which subsequently approaches the β‐carbon of the BCB of **11** from the *exo* face (giving ion pair **20**). With the C−O bond of the alkoxide and the central C−C bond of the bicyclo[1.1.0]butane motif in anti‐periplanar alignment, C−C σ‐bond alkoxypalladation occurs with simultaneous formation of a new C−O bond at the α‐carbon,^[16]^ cleavage of the strained central C−C bond of the BCB, and palladation of the β‐carbon. Finally, reductive elimination of complex **21** releases 1‐oxaspiro[2.3]hexane **18** and regenerates Pd^0^ to complete the catalytic cycle.

One of the driving forces of this reaction is the release of strain energy upon cleavage of the central C−C σ‐bond of the bicyclo[1.1.0]butane in **11**. Interestingly, the 1‐oxaspiro[2.3]hexane products also exhibit considerable strain energy, which is comparable to their hydrocarbon analogue spiro[2.3]hexane (≈56 kcal mol^−1^).^[17]^ However, the exceptionally high strain energy of the bicyclo[1.1.0]butane framework (≈66 kcal mol^−1^) means that a substantial strain‐release still occurs.^[18]^ The preference for approach of palladium complex **19** to the *exo* face of BCB **11** over the *endo* face originates from the central C−C σ‐bond bond of the bicyclo[1.1.0]butane being composed of largely unhybridized 2*p*‐orbitals, leading to increased electron density on the *exo* face of the β‐carbon.^[16]^ Additionally, the steric bulk of the carbinolate moiety on the α‐carbon hinders approach to the *endo* face. These factors result in excellent diastereoselectivity for cyclobutane formation with the oxygen and aryl substituents in a *cis* relationship. Notably, only one diastereomer was observed for all products synthesized.

**Scheme 4:**
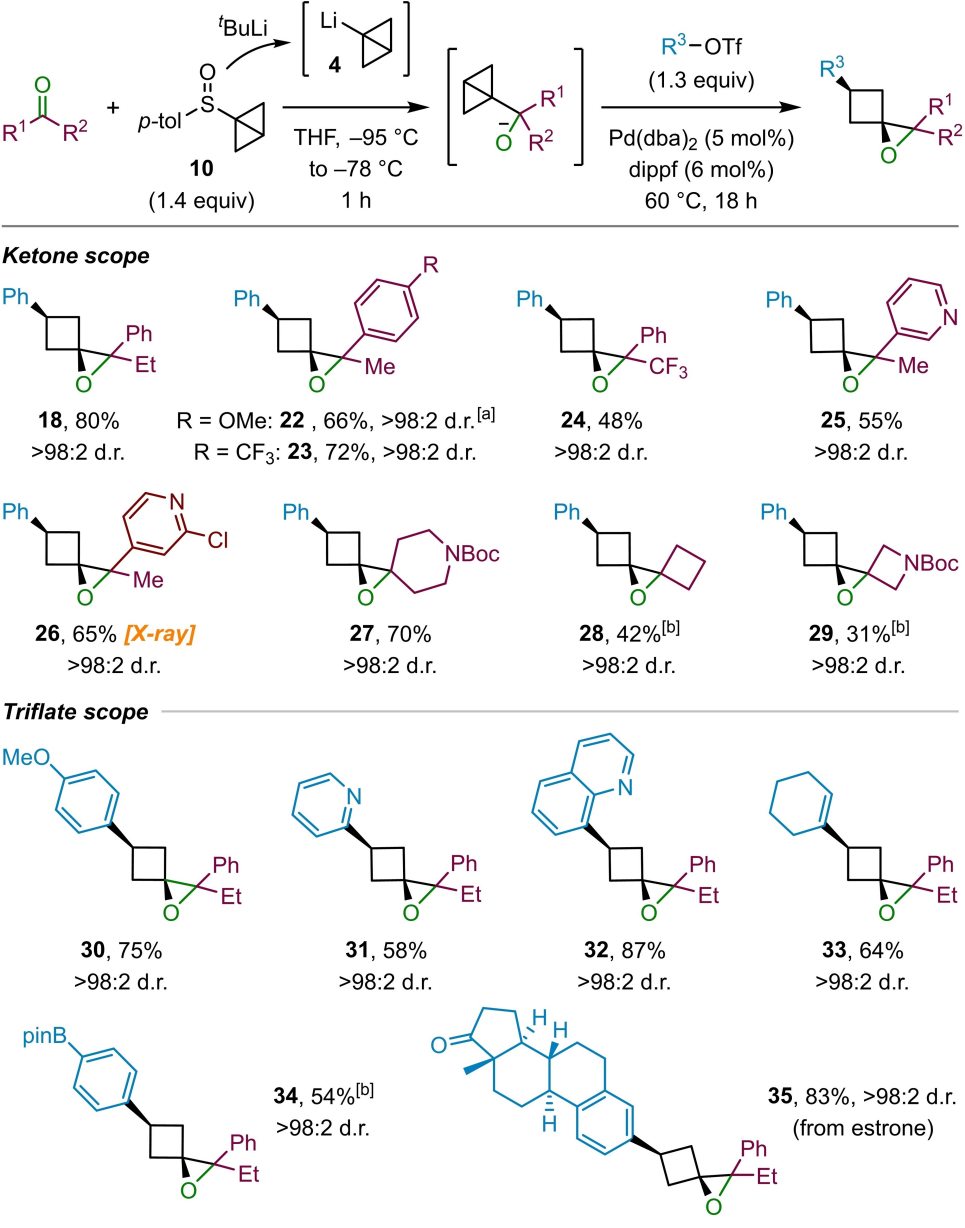
Scope of ketones and triflates employed in the epoxide formation. Reactions performed on 0.3 mmol scale. [a] Yield determined by ^1^H NMR analysis. [b] Catalyst loading: Pd(dba)_2_ (10 mol %), dippf (12 mol %).

Having established a route towards 1‐oxaspiro[2.3]hexane derivatives, we investigated the scope of ketones that could be employed (Scheme 4). In addition to propiophenone (**9**), which provided epoxide **18** in high yield, a number of other aryl ketones containing electron‐deficient aromatic and heteroaromatic substituents gave the corresponding epoxides **23**–**26** in moderate to good yields. The relative stereochemistry of epoxide **26** was determined by X‐ray analysis and all others are assigned by analogy.^[19]^ In the case of ketones containing electron‐rich aromatic substituents, such as **22**, the epoxide product was prone to rearrangement to cyclopentanones (see Scheme 1A) during purification on silica gel;[^2^, ^20^] deactivation of the silica with triethylamine prior to flash column chromatography could not prevent this rearrangement. In this case an NMR yield is provided. Aliphatic cyclic ketones were also found to be feasible substrates for the reaction, which provided dispiro systems **27**–**29** in moderate to good yields. Although the dispiro epoxides **28** and **29** required increased catalyst loading to obtain only moderate yields, their successful formation from cyclobutanone and 1‐Boc‐3‐azetidinone, respectively, is remarkable considering the strain energy of these ring systems is estimated to be ≈83 kcal mol^−1^ (by comparison to their hydrocarbon analogues),^[17]^ which is significantly higher than that of the bicyclo[1.1.0]butane starting material (≈66 kcal mol^−1^).^[18]^

Next, we explored the scope of the aryltriflates, using propiophenone (**12**) as the ketone (Scheme 4). The electron‐rich 4‐methoxy phenyl triflate gave the corresponding product **30** in good yield. In the case of electron‐deficient aryl triflates, containing electron‐withdrawing substituents in the para position such as acetyl and trifluoromethyl groups, no conversion was observed. However, heterocyclic triflates, such as 2‐pyridyl (**31**) and 8‐quinolyl triflate (**32**) were found to be suitable substrates. Cyclohexenyl triflate (**33**) also worked well, providing the corresponding alkenyl‐substituted cyclobutane in good yield. A boronic ester substituent on the aryl triflate (**34**) was tolerated, providing a convenient handle for further cross‐coupling reactions. Lastly, pushing the limitations of this methodology, we tested a more complex aryl triflate derived from the natural product estrone (**35**), which was transformed into the corresponding epoxide in excellent yield.

**Scheme 5:**
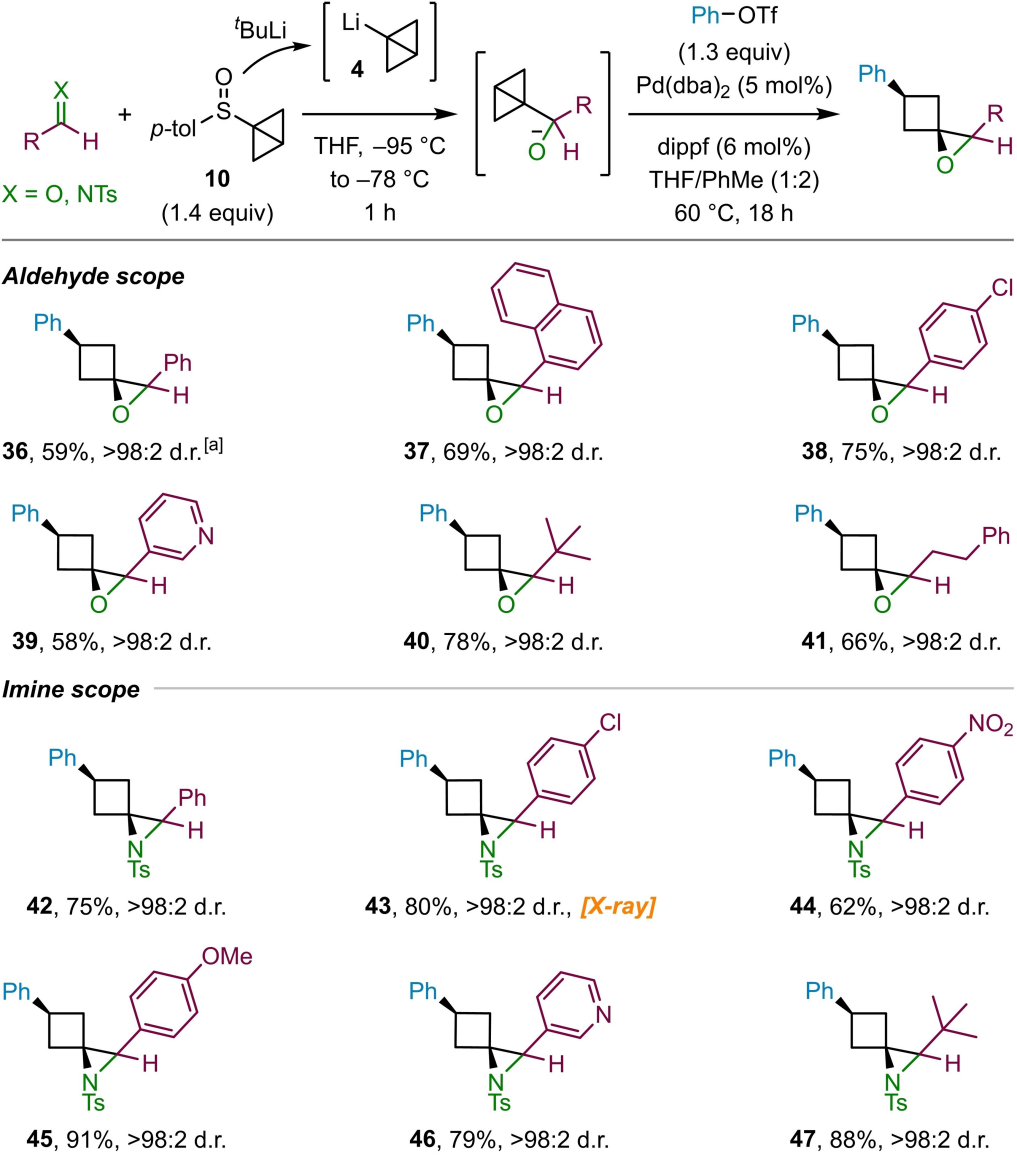
Scope of aldehydes and *N*‐tosyl imines employed in the epoxide and aziridine formation. Reactions performed on 0.3 mmol scale. [a] 2.0 mmol scale.

Having explored the scope of ketones and triflates we sought to extend our methodology to also accommodate aldehydes as substrates (Scheme 5). However, whilst the formation of the carbinolate intermediate proceeded as planned, the subsequent palladium‐catalyzed spirocyclization only gave poor yields of products. In exploring other conditions, we discovered that the addition of toluene as a co‐solvent proved to be beneficial, with reactions performed in a 1 : 2 mixture of THF/toluene giving high yields of the corresponding epoxides. By applying these adjusted reaction conditions, a range of aldehydes could be successfully used. Showcasing the efficiency of the modified procedure, the reaction of benzaldehyde was performed on a 2 mmol scale to give **36** in 59 % yield. Epoxides derived from aromatic aldehydes, such as 1‐naphthaldehyde (**37**) and 4‐chlorobenzaldehyde (**38**), as well as the heteroaromatic aldehyde nicotinaldehyde (**39**), were formed in good yields. Additionally, aliphatic aldehydes, including pivalaldehyde (**40**) and dihydrocinnamaldehyde (**41**), were tolerated and furnished the corresponding epoxides in high yields.

As nitrogen containing three‐dimensional organic molecules are of particular interest in medicinal chemistry,^[21]^ we were eager to explore whether imines could be used in place aldehydes, thus expanding our product portfolio to also include 1‐azaspiro[2.3]hexane derivatives. Indeed, we discovered that the modified reaction conditions developed for aldehydes could be directly translated to *N*‐tosyl imines (Scheme 5), with the benzaldehyde‐derived imine providing aziridine **42** in high yield. Imines containing electron‐deficient aryl substituents, such as 4‐chlorophenyl (**43**, X‐ray^[22]^) and 4‐nitrophenyl (**44**), were also good substrates for the aziridination reaction. Notably, aziridines containing the electron‐rich 4‐methoxyphenyl substituent (**45**) were obtained in excellent yield and proved to be sufficiently stable on silica for efficient purification, contrasting the analogous ketone substrate **22** that failed due to the tendency of electron‐rich epoxides to undergo rearrangement to cyclopentanones (see above). Furthermore, 3‐pyridinyl (**46**) and *tert*‐butyl (**47**) containing aziridines were formed in good to excellent yield.

**Scheme 6:**
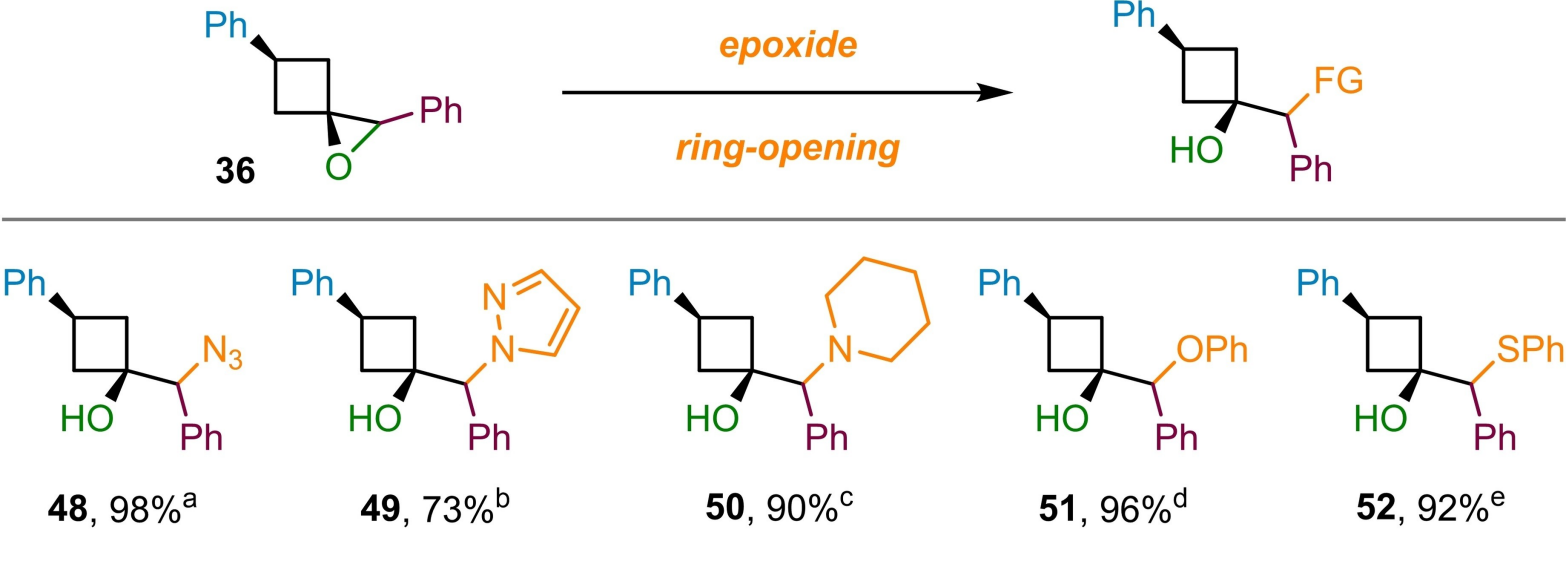
Derivatization of the epoxide products via ring‐opening reactions. Abbreviated reaction conditions are indicated as footnotes: [a] NH_4_Cl, NaN_3_; [b] pyrazole, NaH; [c] piperidine, triethylamine; [d] phenol, NaH; [e] sodium thiophenolate.

Lastly, we investigated the synthetic utility of the 1‐oxaspiro[2.3]hexane products (Scheme 6). Ring‐opening reactions of the epoxide functionality in 1‐oxaspiro[2.3]hexanes are increasingly used for the introduction of hydroxy‐substituted cyclobutyl groups into target structures.^[4]^ Indeed, a Reaxys search identified more than 20 patents that utilize such reactions for the synthesis of biologically active compounds in the last 15 years. Furthermore, the aspect of introducing a broad variety of nucleophiles through epoxide opening reactions unlocks an additional layer of diversity to our versatile methodology. We initially explored nitrogen‐based nucleophiles for epoxide opening because nitrogen‐containing motifs, such as N‐heterocycles, are prominently featured in pharmaceutical target molecules.^[21]^ Pleasingly, high yielding reactions where possible with azide (**48**) and N‐heterocycles, such as pyrazole (**49**) and piperidine (**50**). In addition, the use of other heteroatom‐based nucleophiles, including phenolate (**51**) and thiolate (**52**), gave the corresponding cyclobutanols in excellent yields. The strain‐release associated with the epoxide ring‐opening appears to be an important driving force for the reaction, delivering excellent yields despite the sterically demanding nature of trisubstituted epoxides.

## Conclusion

In summary, we successfully developed a new methodology for the efficient production of spirocyclic epoxides and aziridines through a three‐component coupling reaction between readily available BCB sulfoxide **10**, ketones, aldehydes or imines, and aryl/alkenyl triflates. Careful optimization of the initial lithiation of **10** was required to avoid competing reaction pathways during the formation of the key BCB carbinolate intermediate, which enabled the subsequent palladium‐catalyzed cross‐coupling with triflates to proceed with high efficiency. The resulting 1‐oxaspiro[2.3]hexane and 1‐azaspiro[2.3]hexane products where formed as single diastereomers due to a highly stereoselective C−C σ‐bond nucleopalladation.

The reaction tolerated a broad variety of both aromatic and aliphatic ketones, and a diverse set of aryl triflates. In addition, after simple optimization of the solvent used in the reaction, aromatic and aliphatic aldehydes and *N*‐tosyl imines were also demonstrated to be excellent substrates. Especially noteworthy findings of this work are the dispiro compounds, derived from cyclobutanone (**28**) and 1‐Boc‐3‐azetidione (**29**), which appear to possess strain energy surpassing their bicyclo[1.1.0]butane precursor, implying a “strain‐increase spirocyclization”. Lastly, through several high yielding epoxide ring‐opening reactions with different nucleophiles, we demonstrated the applicability of the 1‐oxaspiro[2.3]hexane products as building blocks for the convenient introduction of the three‐dimensional cyclobutyl motif into target molecules.

## Conflict of interest

The authors declare no conflict of interest.

1

## Supporting information

As a service to our authors and readers, this journal provides supporting information supplied by the authors. Such materials are peer reviewed and may be re‐organized for online delivery, but are not copy‐edited or typeset. Technical support issues arising from supporting information (other than missing files) should be addressed to the authors.

Supporting Information

## Data Availability

The data that support the findings of this study are available in the Supporting Information of this article.
