# Supplementary material for: Strain‐Release Driven Epoxidation and Aziridination of Bicyclo[1.1.0]butanes via Palladium Catalyzed σ‐Bond Nucleopalladation
Source: Angew Chem Weinheim Bergstr Ger. 2023 Jan 12;135(7):e202217064. doi: 10.1002/ange.202217064 (PMC10952369; doi:10.1002/ange.202217064)
Supplement: Supplementary file 1 — Supporting Information [file ANGE-135-0-s001.pdf]

Angewandte Chemie
Eine Zeitschrift der Gesellschaft Deutscher Chemiker

Supporting Information

Strain-Release Driven Epoxidation and Aziridination of Bicyclo[1.1.0]butanes via Palladium Catalyzed σ-Bond Nucleopalladation

*B. Wölfl, N. Winter, J. Li, A. Noble, V. K. Aggarwal**

TABLE OF CONTENTS

LIST OF SUPPLEMENTARY SCHEMES, FIGURES AND TABLES 2

LIST OF CHARACTERISED PRODUCTS 2

1. MATERIALS AND GENERAL METHODS 4

- 1.1. Glassware, Solvents and Reagents 4
- 1.2. Chromatography and Instrumentation 4
- 1.3. Naming of Compounds 4

2. EXPERIMENTAL DATA 5

- 2.1. Reaction Optimisation 5
  - 2.1.1. Experimental data for Entry 3: 5
  - 2.1.2. Side Products for Other Entries: 6
- 2.2. Synthesis of Starting material and Substrates 8
- 2.3. General Procedures 9
  - 2.3.1. General Procedure A: Ketone Scope 9
  - 2.3.2. General Procedure B: Triflate Scope 9
  - 2.3.3. General Procedure C: Aldehyde and Imine Scope 10
  - 2.3.4. General Notes 10
- 2.4. Substrate Scope 11
- 2.5. Derivatisation Reactions 30
- 2.6. Unsuccessful Substrates 35

3. X-RAY CRYSTALLOGRAPHIC ANALYSIS 36

- 3.1. 26 (CCDC number: 2115071) and 43 (CCDC number: 2115072) 36

4. SPECTROSCOPIC DATA 38

5. REFERENCES 77

LIST OF SUPPLEMENTARY SCHEMES, FIGURES AND TABLES

Table 1: Investigation of carbinolate formation 5
Figure 1. Synthesised Substrates 8
Figure 2. Unsuccessful Substrates 35
Table 2: Crystal data and structure refinement for 26 and 43 36
Figure 3. Crystal structure of 26 with the anisotropic displacement parameters depicted at the 50% probability level and hydrogens omitted for clarity. 37
Figure 4. Crystal structure of 43 with the anisotropic displacement parameters depicted at the 50% probability level. Disorder and hydrogens omitted for clarity. 37

LIST OF CHARACTERISED PRODUCTS

1-(bicyclo[1.1.0]butan-1-yl)-1-phenylpropan-1-ol (12) 5
2,2-dimethyl-3-phenylpentan-3-ol (13) 6
1-phenyl-1-(*p*-tolyl)propan-1-ol (14) 6
1-(*tert*-butylsulfinyl)-4-methylbenzene (15) 7
cis-2-ethyl-2,5-diphenyl-1-oxaspiro[2.3]hexane (18) 11
cis-2-(4-methoxyphenyl)-2-methyl-5-phenyl-1-oxaspiro[2.3]hexane (22) 12
cis-2-methyl-5-phenyl-2-(4-(trifluoromethyl)phenyl)-1-oxaspiro[2.3]hexane (23) 12
cis-2,5-diphenyl-2-(trifluoromethyl)-1-oxaspiro[2.3]hexane (24) 13
3-(*cis*-2-methyl-5-phenyl-1-oxaspiro[2.3]hexan-2-yl)pyridine (25) 13
2-chloro-4-(*cis*-2-methyl-5-phenyl-1-oxaspiro[2.3]hexan-2-yl)pyridine (26) 14
tert-butyl *cis*-2-phenyl-11-oxa-8-azadispiro[3.0.5^5.1^4]undecane-8-carboxylate (27) 15
cis-2-phenyl-9-oxadispiro[3.0.3^5.1^4]nonane (28) 15
tert-butyl *cis*-7-phenyl-9-oxa-2-azadispiro[3.0.3^5.1^4]nonane-2-carboxylate (29) 16
cis-2-ethyl-5-(4-methoxyphenyl)-2-phenyl-1-oxaspiro[2.3]hexane (30) 16
2-(*cis*-2-ethyl-2-phenyl-1-oxaspiro[2.3]hexan-5-yl)pyridine (31) 17
8-(*cis*-2-phenyl-1-oxaspiro[2.3]hexan-5-yl)quinoline (32) 18
cis-5-(cyclohex-1-en-1-yl)-2-ethyl-2-phenyl-1-oxaspiro[2.3]hexane (33) 18
4,4,5,5-tetramethyl-2-(4-(*cis*-2-phenyl-1-oxaspiro[2.3]hexan-5-yl)phenyl)-1,3,2-dioxaborolane (34) 19
(8*R*,9*S*,13*S*,14*S*)-3-(*cis*-2-ethyl-2-phenyl-1-oxaspiro[2.3]hexan-5-yl)-13-methyl-6,7,8,9,11,12,13,14,15,16-decahydro-17*H*-cyclopenta[a]phenanthren-17-one (35) 20
cis-2,5-diphenyl-1-oxaspiro[2.3]hexane (36) 21
cis-2-(naphthalen-1-yl)-5-phenyl-1-oxaspiro[2.3]hexane (37) 22
cis-2-(4-chlorophenyl)-5-phenyl-1-oxaspiro[2.3]hexane (38) 23
cis-5-phenyl-1-oxaspiro[2.3]hexan-2-yl)pyridine (39) 23
cis-2-(*tert*-butyl)-5-phenyl-1-oxaspiro[2.3]hexane (40) 24
cis-2-phenethyl-5-phenyl-1-oxaspiro[2.3]hexane (41) 25
cis-2,5-diphenyl-1-tosyl-1-azaspiro[2.3]hexane (42) 25
cis-2-(4-chlorophenyl)-5-phenyl-1-tosyl-1-azaspiro[2.3]hexane (43) 26
cis-2-(4-nitrophenyl)-5-phenyl-1-tosyl-1-azaspiro[2.3]hexane (44) 27
cis-2-(4-methoxyphenyl)-5-phenyl-1-tosyl-1-azaspiro[2.3]hexane (45) 27
cis-5-phenyl-2-(pyridin-3-yl)-1-tosyl-1-azaspiro[2.3]hexane (46) 28
cis-2-(*tert*-butyl)-5-phenyl-1-tosyl-1-azaspiro[2.3]hexane (47) 29

cis-1-(azido(phenyl)methyl)-3-phenylcyclobutan-1-ol (48)[11] 30
cis-3-phenyl-1-(phenyl(1*H*-pyrazol-1-yl)methyl)cyclobutan-1-ol (49)[12] 31
cis-3-phenyl-1-(phenyl(piperidin-1-yl)methyl)cyclobutan-1-ol (50)[14] 32
cis-1-(phenoxy(phenyl)methyl)-3-phenylcyclobutan-1-ol (51)[12] 33
cis-3-phenyl-1-(phenyl(phenylthio)methyl)cyclobutan-1-ol (52)[15] 34

1. MATERIALS AND GENERAL METHODS

1.1. Glassware, Solvents and Reagents

All manipulations were performed with oven-dried (130 °C for a minimum of 12 h) or flame-dried glassware using standard Schlenk techniques under an atmosphere of nitrogen, unless otherwise stated.

All anhydrous solvents were commercially supplied or dried using an Anhydrous Engineering alumina column drying system (THF, toluene, Et_2O, CH_2Cl_2). Reagents were purchased from commercial sources and used as received. Bis(dibenzylideneacetone)palladium(0) ($Pd(dba)_2$) [CAS: *32005-36-0*] and 1,1'-bis (diisopropylphosphino)ferrocene (dippf) [CAS: *97239-80-0*] were purchased from Sigma-Aldrich and used as received. All liquid ketone reagents were filtered through a plug of neutral alumina prior to use. All liquid aldehyde reagents were distilled prior to use. All organolithium reagents were titrated against *N*-benzylbenzamide.[1]

1.2. Chromatography and Instrumentation

Thin layer chromatography (TLC) was performed using Merck Kieselgel 60 F254 fluorescent treated silica, which was visualised under UV light, or by staining with aqueous basic potassium permanganate followed by heating, *p*-anisaldehyde solution followed by heating, Hanessian's stain (CAM stain) followed by heating, or an ethanolic solution of phosphomolybdic acid followed by heating, as stated.
Flash column chromatography (FCC) was carried out using Sigma-Aldrich silica gel (60 Å, 230–400 mesh, 40–63 µm) or a Biotage Isolera™ flash purification system. In cases where automated column chromatography was employed the solvent gradient and flow rate are indicated.
NMR spectra were recorded at various field strengths, as indicated, using Bruker 400 MHz, Varian VNMR 400 MHz, Varian VNMR 500 MHz, or Bruker Cryo 500 MHz for ^{1}H, ^{11}B, ^{13}C and ^{19}F acquisitions. All NMR spectra were recorder at 25 °C unless otherwise stated. Chemical shifts (δ) are reported in parts per million (ppm) and referenced $CDCl_3$ (^{1}H: 7.26 ppm; ^{13}C: 77.16 ppm) or DMSO-d_6 (^{1}H: 2.50 ppm; ^{13}C: 39.5 ppm). Coupling constants (*J*) are given in Hertz (Hz) and refer to apparent multiplicities (s = singlet, d = doublet, t = triplet, q = quartet, p = pentet, sex = hextet, h = heptet, m = multiplet, br = broad signal, dd = doublet of doublets, etc.). The ^{1}H NMR spectra are reported as follows: chemical shift (multiplicity, coupling constants, number of protons). NMR yields were determined by ^{1}HNMR analysis using dibromomethane as an internal standard. Diastereomeric ratios (d.r.) were determined by ^{1}H NMR analysis of the crude reaction mixture.
High resolution mass spectra (HRMS) were recorded on a Bruker Daltonics MicrOTOF II by Electrospray Ionisation (ESI); a Thermo Scientific QExactive by Electron Ionisation (EI); a Thermo Scientific Orbitrap Elite by ESI or Atmospheric Pressure Chemical Ionisation (APCI); or a Bruker UltrafleXtreme by Matrix-assisted Laser Desorption/Ionisation (MALDI).
IR spectra were recorded neat as a thin film on a Perkin Elmer Spectrum One FT-IR. Selected absorption maxima (ν_{max}) are reported in wavenumbers (cm^{-1}).

1.3. Naming of Compounds

Compound names are those generated by ChemDraw Professional 20.0 software (PerkinElmer), following the IUPAC nomenclature.

2. EXPERIMENTAL DATA

2.1. Reaction Optimisation

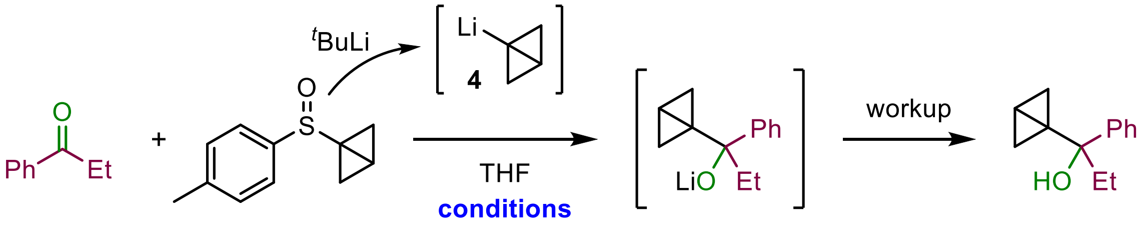

9 **10** (1.4 equiv) **11** **12**

Entry	Conditions	12	Side Product
1	*in situ*, –100 °C	68%	23% (**13**)
2	*ex situ*, –78 °C, ketone added 10 min after ^{t}BuLi	30%	40% (**14**)
3	*ex situ*, –95 °C, ketone added 1 min after ^{t}BuLi	92%	0%

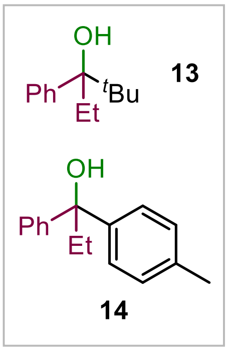

Table 1: Investigation of carbinolate formation

2.1.1. Experimental data for Entry 3:

1-(bicyclo[1.1.0]butan-1-yl)-1-phenylpropan-1-ol (12)

12
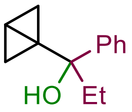

tert-Butyl lithium (1.7 M in pentane, 80.0 µL, 0.136 mmol, 1.36 equiv) was added dropwise (within 1 min) to a solution of BCB-sulfoxide **10** (26.0 mg, 0.135 mmol, 1.35 equiv) in THF (1.00 mL) at –95 °C (acetone/liquid nitrogen) and stirred vigorously for 1 min, before a solution of propiophenone (13.3 µL, 13.4 mg, 0.100 mmol, 1.00 equiv) in THF (0.300 mL) was added dropwise (within 1 min). Dry ice was added to the cooling bath and stirring continued for 1 h. After the reaction was allowed to warm to room temperature, water was added to quench the reaction, then Et_2O was added, and the phases were separated. The aqueous phase was extracted with Et_2O (3 x) and the combined organic phases were dried over $MgSO_4$, filtered, and concentrated under reduced pressure. A colourless oil was obtained as crude product, which could not be purified via flash column chromatography due to instability of the product on SiO_2. The yield was determined by NMR using CH_2Br_2 (7.0 µL, 17 mg, 0.10 mmol) as internal standard. ^{1}H NMR yield: 92%. Isolated yield: 0%.

NMR Spectroscopy of crude product (*see spectra*):

crude ^{1}H NMR (400 MHz, $CDCl_3$): δ_H 7.45 – 7.42 (m, 2H), 7.34 – 7.30 (m, 2H), 7.24 – 7.20 (m, 1H), 1.93 (qd, *J* = 7.4, 4.7 Hz, 2H), 1.60 – 1.58 (m, 1H), 1.50 (dd, *J* = 2.8, 0.9 Hz, 2H), 0.81 (t, *J* = 7.4 Hz, 3H), 0.60 (dd, *J* = 1.5, 0.7 Hz, 1H), 0.52 (dd, *J* = 1.5, 0.7 Hz, 1H) ppm. The crude product also contained sulfoxide **15** as major byproduct, as well as other impurities.

2.1.2. Side Products for Other Entries:

2,2-dimethyl-3-phenylpentan-3-ol (13)

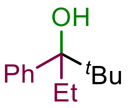

13

NMR Spectroscopy (*see spectra*):

^{1}**H NMR** (400 MHz, $CDCl_3$): δ_H 7.39 – 7.36 (m, 2H), 7.33 – 7.29 (m, 2H), 7.24 – 7.20 (m, 1H), 2.23 (dq, *J* = 14.8, 7.4 Hz, 1H), 1.87 (dq, *J* = 14.4, 7.3 Hz, 1H), 1.68 (s, 1H), 0.91 (s, 9H), 0.68 (t, *J* = 7.3 Hz, 3H) ppm;

^{13}**C NMR** (101 MHz, $CDCl_3$): δ_C 143.0, 127.9, 127.2, 126.3, 81.4, 38.5, 27.1, 26.0, 8.3 ppm.

IR (film): ν_{max} 3607, 2968, 2877, 1446, 1364, 968 cm^{-1}.

HRMS ($APCI^+$): m/z calc'd for $C_{13}H_{20}O$ $[M+H]^+$, 193.1587; found, 193.1587.

1-phenyl-1-(*p*-tolyl)propan-1-ol (14)

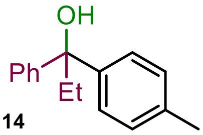

14

NMR Spectroscopy (*see spectra*):

^{1}**H NMR** (400 MHz, $CDCl_3$): δ_H 7.43 – 7.40 (m, 2H), 7.33 – 7.28 (m, 4H), 7.24 – 7.19 (m, 1H), 7.13 – 7.11 (m, 2H), 2.34 – 2.28 (m, 5H), 2.32 (s, 1H), 2.31 (q, *J* = 7.3 Hz, 2H), 2.04 (s, 1H), 0.89 (t, *J* = 7.3 Hz, 3H) ppm;

^{13}**C NMR** (101 MHz, $CDCl_3$): δ_C 147.2, 144.2, 136.5, 129.0, 128.2, 126.8, 126.2, 126.2, 78.5, 34.6, 21.1, 8.3 ppm.

All recorded spectroscopic data matched those previously reported in the literature.[2]

1-(*tert*-butylsulfinyl)-4-methylbenzene (15)

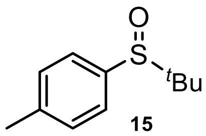

15

NMR Spectroscopy (*see spectra*):

^{1}H NMR (400 MHz, $CDCl_3$): δ_H 7.48 – 7.46 (m, 2H), 7.29 – 7.27 (m, 2H), 2.41 (s, 3H), 1.16 (s, 9H) ppm;

^{13}C NMR (101 MHz, $CDCl_3$): δ_C 141.7, 136.9, 129.2, 126.4, 55.8, 22.9, 21.6 ppm.

All recorded spectroscopic data matched those previously reported in the literature.[3]

2.2. Synthesis of Starting material and Substrates

Bicyclo[1.1.0]butyl sulfoxide **10**[4] and substrates to generate compounds **32**,[5] **34**,[6] **35**,[7] **43**,[8] **44**,[8] **45**,[8] **46**[9] and **47**[10] were prepared according to literature procedures indicated in Figure S1. All recorded spectroscopic data matched those previously reported in the literature.

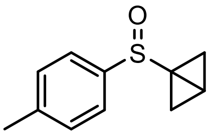

10
J. Am. Chem. Soc.
2019, *141,* 9511-9515.

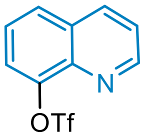

S32
Chem. Eur. J.
2013, *19,* 3504-3511.

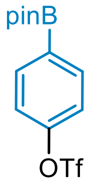

S34
J. Am. Chem. Soc.
2019, *141,* 14126-14130

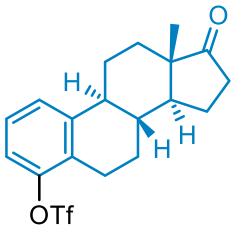

S35
Angew. Chem. Int. Ed.
2019, *58,* 7318-7323

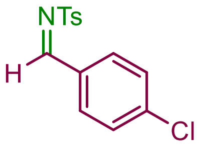

S43
J. Am. Chem. Soc.
2014, *136,* 1082-1089.

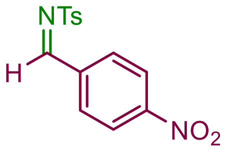

S44
J. Am. Chem. Soc.
2014, *136,* 1082-1089.

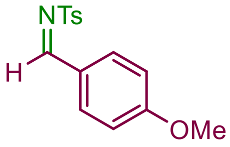

S45
J. Am. Chem. Soc.
2014, *136,* 1082-1089.

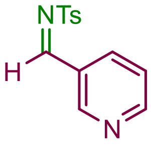

S46
Organic Chemistry Frontiers
2020, *7,* 578-583.
For reference spectra see:
J. Org. Chem. **1998,** *63*, 2800-2801.

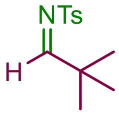

S47
J. Org. Chem.
2004, *69*, 1409-1412.

Figure 1. Synthesised Substrates

2.3. General Procedures

2.3.1. General Procedure A: Ketone Scope

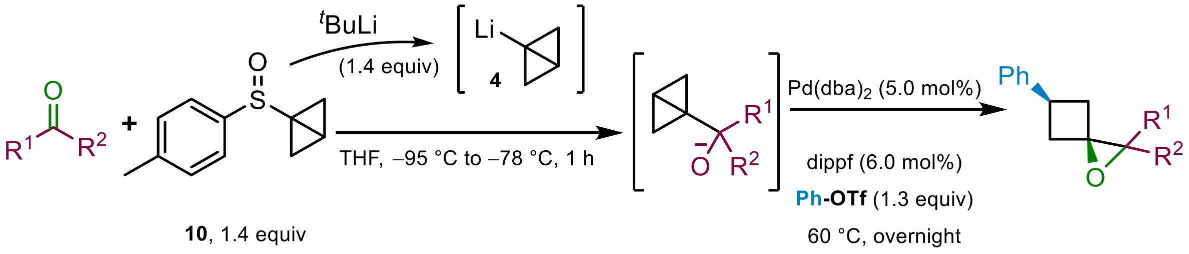

tert-Butyl lithium (in pentane, 0.405 mmol, 1.35 equiv)[A] was added dropwise[B] to a solution of BCB-sulfoxide **10** (77.9 mg, 0.405 mmol, 1.35 equiv) in THF[C] (3.00 mL) at –95 °C (acetone/liquid nitrogen) and stirred vigorously for 1 min,[D] before a solution of ketone (0.300 mmol, 1.00 equiv) in THF[C] (0.900 mL) was added dropwise.[B]. Dry ice was added to the cooling bath and stirring continued for 1 h. After removing the cooling bath phenyl trifluoromethanesulfonate (63.0 µL, 88.2 mg, 0.39 mmol, 1.30 equiv) was added, followed by.a solution[E] of Pd(dba)$_2$ (8.6 mg, 15 µmol, 5.0 mol%) and dippf (7.5 mg, 18 µmol µmol, 6.0 mol%) in THF[C] (0.900 mL) (pre-mixed under nitrogen for 45 min). The flask was sealed and heated at 60 °C (oil bath) for 18 h.[F] Water was added to quench the reaction, then Et_2O[G] was added, and the phases were separated. The aqueous phase was extracted with Et_2O (3 x)[G] and the combined organic phases were dried over $MgSO_4$, filtered, and concentrated under reduced pressure. The crude material was purified by flash column chromatography.

Notes: see *2.3.4. General Notes*

2.3.2. General Procedure B: Triflate Scope

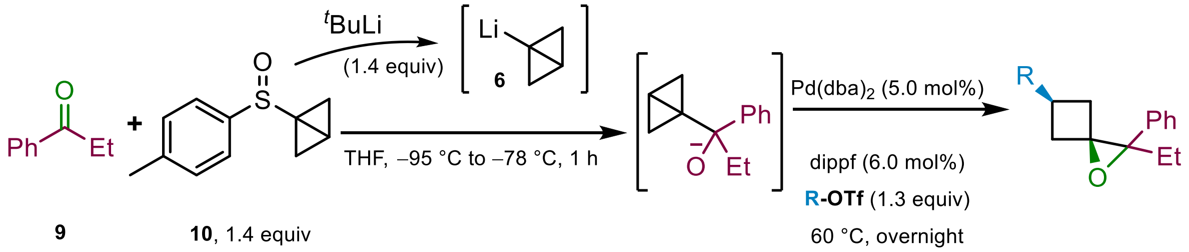

tert-Butyl lithium (in pentane, 0.405 mmol, 1.35 equiv)[A] was added dropwise[B] to a solution of BCB-sulfoxide **10** (77.9 mg, 0.405 mmol, 1.35 equiv) in THF[C] (3.00 mL) at –95 °C (acetone/liquid nitrogen) and allowed to stir for 1 min,[D] before a solution of propiophenone (40.0 µL, 40.0 mg, 0.300 mmol, 1.00 equiv) in THF[C] (0.900 mL) was added dropwise.[B]. Dry ice was added to the cooling bath and stirring continued for 1 h. After removing the cooling bath, the triflate (0.39 mmol, 1.30 equiv) was added, followed by a solution[E] of Pd(dba)$_2$ (8.6 mg, 15 µmol, 5.0 mol%) and dippf (7.5 mg, 18 µmol µmol, 6.0 mol%) in THF[C] (0.900 mL) (pre-mixed under nitrogen for 45 min). The flask was sealed and heated at 60 °C (oil bath) for 18 h.[F] Water was added to quench the reaction,

then Et_2O[G] was added, and the phases were separated. The aqueous phase was extracted with Et_2O (3 x)[G] and the combined organic phases were dried over $MgSO_4$, filtered, and concentrated under reduced pressure. The crude material was purified by flash column chromatography.

Notes: see *2.3.4. General Notes*

2.3.3. General Procedure C: Aldehyde and Imine Scope

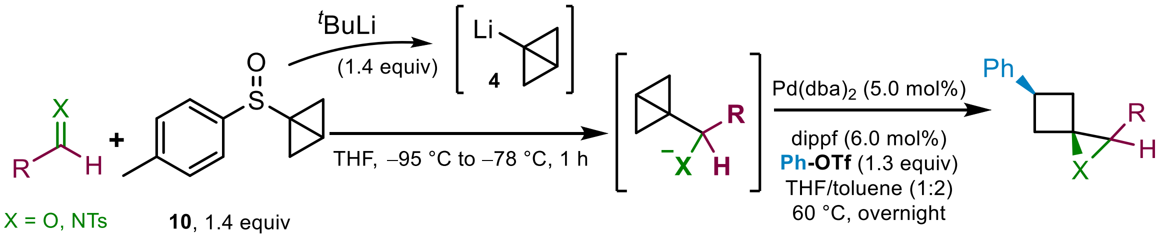

tert-Butyl lithium (in pentane, 0.405 mmol, 1.35 equiv)[A] was added dropwise[B] to a solution of BCB-sulfoxide **10** (77.9 mg, 0.405 mmol, 1.35 equiv) in THF[C] (3.00 mL) at –95 °C (acetone/liquid nitrogen) and allowed to stir for 1 min,[D] before a solution of aldehyde or *N*-tosyl imine[H] (0.300 mmol, 1.00 equiv) in THF[C] (0.900 mL) was added dropwise.[B]. Dry ice was added to the cooling bath and stirring continued for 1 h. After removing the cooling bath toluene[C] (7 mL) and phenyl trifluoromethanesulfonate (63.0 µL, 88.2 mg, 0.39 mmol, 1.30 equiv) were added, followed by.a solution[E] of $Pd(dba)_2$ (8.6 mg, 15 µmol, 5.0 mol%) and dippf (7.5 mg, 18 µmol µmol, 6.0 mol%) in toluene[C] (0.900 mL) (pre-mixed under nitrogen for 1 h). The flask was sealed and heated at 60 °C (oil bath) for 18 h.[F] Water was added to quench the reaction, then Et_2O[G] was added, and the phases were separated. The aqueous phase was extracted with Et_2O (3 x)[G] and the combined organic phases were dried over $MgSO_4$, filtered, and concentrated under reduced pressure. The crude material was purified by flash column chromatography.

2.3.4. General Notes

(**A**) *tert*-Butyl lithium should be carefully titrated prior to use.[1] (**B**) On this scale this dropwise addition takes approximately 1 min. On larger scale (2 mmol) the addition time was extended to 2 min. (**C**) Anhydrous and degassed solvent (freeze/pump/thaw, 3 cycles). (**D**) Lower time intervals may lead to the formation of side products (e.g. *in situ* side product **13**). On larger scale (2 mmol) the time interval between the additions was extended to 2 min. (**E**) The catalyst solution was prepared in a flame dried Schlenk flask by dissolving $Pd(dba)_2$ and dippf in anhydrous degassed solvent and stirring at room temperature under nitrogen until a clear orange solution was formed. (**F**) While stirring overnight the reaction typically changed colour from orange to brown. (**G**) Et_2O was most commonly used for extractions. However, if products containing *N*-heterocyclic structures were obtained EtOAc was used for extracting these products instead. (**H**) In case of certain *N*-tosyl imines, due to their lower solubility in THF they were added in lower concentration as indicated in the specific examples.

2.4. Substrate Scope

cis-2-ethyl-2,5-diphenyl-1-oxaspiro[2.3]hexane (18)

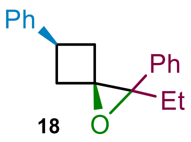

18

Prepared following **General Procedure A**, using propiophenone (40.0 µL, 40.0 mg, 0.300 mmol, 1.00 equiv), dissolved in THF (0.900 mL), as starting material. Biotage Isolera™ flash purification on silica gel (Sfär High Capacity 25 g silica cartridge), eluting with EtOAc/hexane (0 – 2%, v/v) gave epoxide **18** (63.5 mg, 80%) as a pale-yellow oil.

R_f = 0.22 (2:98 EtOAc/hexane, UV, cerium molybdate)

NMR Spectroscopy (*see spectra*):

> **^{1}H NMR** (400 MHz, $CDCl_3$): δ_H 7.41 – 7.23 (m, 9H), 7.23 – 7.17 (m, 1H), 3.23 (apparent p, *J* = 8.8 Hz, 1H), 2.89 (dddd, *J* = 12.7, 8.5, 4.2, 1.3 Hz, 1H), 2.63 (ddd, *J* = 12.7, 9.0, 1.3 Hz, 1H), 2.41 (ddd, *J* = 12.8, 9.0, 1.3 Hz, 1H), 2.31 (dq, *J* = 14.1, 7.5 Hz, 1H), 2.19 (dddd, *J* = 12.8, 8.5, 4.2, 1.3 Hz, 1H), 1.58 (dq, *J* = 14.7, 7.4 Hz, 1H), 0.97 (t, *J* = 7.4 Hz, 3H) ppm;

> **^{13}C NMR** (101 MHz, $CDCl_3$): δ_C 144.9, 138.4, 128.6, 128.3, 127.2, 126.7, 126.3, 68.1, 67.5, 37.3, 31.4, 26.6, 9.3 ppm.

IR (film): ν_{max} 3060, 3027, 2970, 2933, 1603, 1496, 1450, 1297 cm^{-1}.

HRMS (ESI$^+$): m/z calc'd for $C_{19}H_{21}O$ $[M+H]^+$,265.1587; found, 265.1583.

cis-2-(4-methoxyphenyl)-2-methyl-5-phenyl-1-oxaspiro[2.3]hexane (22)

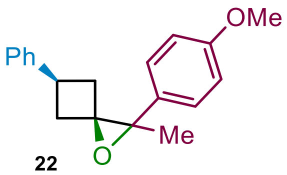

22

Prepared following **General Procedure A**, using 4'-methoxyacetophenone (45.1 mg, 0.300 mmol, 1.00 equiv), dissolved in THF (0.900 mL), as starting material. A brown oil was obtained as crude product, which could not be purified via flash column chromatography due to instability of the product on SiO_2. The yield was determined by NMR using CH_2Br_2 (21.0 µL, 52.0 mg, 0.300 mmol) as internal standard. ^{1}H NMR yield: 66%. Isolated yield: 0%. We also tested Florisil and neutral aluminium oxide for the purification epoxide **22** but the product underwent the Meinwald rearrangement in all cases.

NMR Spectroscopy (_see spectra_):

crude ^{1}H NMR (400 MHz, $CDCl_3$): δ_H 3.81 (s, 3H), 3.21 (apparent p, J = 8.8 Hz, 1H), 2.86 (ddd, J = 13.1, 8.5, 4.0 Hz, 1H), 2.63 (dd, J = 12.8, 9.0 Hz, 1H), 2.49 (dd, J = 12.8, 9.0 Hz, 1H), 2.28 (ddd, J = 13.1, 8.9, 4.0 Hz, 1H), 1.69 (s, 3H).ppm. Protons in the aromatic reagion could not be assigned. The crude product also contained sulfoxide **15** as major byproduct, as well as other impurities.

cis-2-methyl-5-phenyl-2-(4-(trifluoromethyl)phenyl)-1-oxaspiro[2.3]hexane (23)

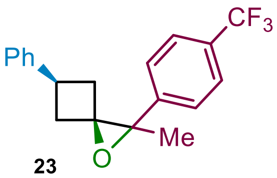

23

Prepared following **General Procedure A**, using 4'-(trifluoromethyl)acetophenone (56.4 mg, 0.300 mmol, 1.00 equiv), dissolved in THF (0.900 mL), as starting material. Biotage Isolera™ flash purification on silica gel (Sfär High Capacity 10 g silica cartridge), eluting with EtOAc/hexane (0 – 8%, v/v) gave epoxide **23** (68.8 mg, 72%) as a white crystalline solid.

m.p.: = 56 – 57 °C (hexane)

R_f = 0.18 (5:95 EtOAc/hexane, UV, cerium molybdate)

NMR Spectroscopy (_see spectra_):

^{1}H NMR (500 MHz, $CDCl_3$): δ_H 7.61 (d, J = 8.1 Hz, 2H), 7.44 (d, J = 8.1 Hz, 2H), 7.33 – 7.30 (m, 2H), 7.26 – 7.19 (m, 3H), 3.21 (apparent p, J = 8.8 Hz, 1H), 2.88 (dddd, J = 12.8, 8.5, 4.2, 1.3 Hz, 1H), 2.66 (ddd, J = 12.9, 9.1, 1.3 Hz, 1H), 2.49 (ddd, J = 12.9, 9.0, 1.3 Hz, 1H), 2.17 (dddd, J = 12.8, 8.6, 4.2, 1.3 Hz, 1H), 1.71 (s, 3H) ppm;

^{13}C NMR (126 MHz, $CDCl_3$): δ_C 144.5, 144.3, 129.6 (q, $^2J_{CF}$ = 32.4 Hz), 128.6, 126.6, 126.5, 126.4, 125.3

(q, $^3J_{CF}$ = 3.8 Hz), 124.3 (q, $^1J_{CF}$ = 272.0 Hz), 67.5, 63.4, 37.1, 36.9, 31.1, 19.7 ppm;

^{19}F NMR (377 MHz, $CDCl_3$): δ_F –62.4 ppm.

IR (film): ν_{max} 2973, 2933, 1324, 1164, 1122, 1078, 1016 cm^{-1}.

HRMS (ESI$^+$): m/z calc'd for $C_{19}H_{17}F_3ONa$ [M+Na]$^+$, 341.1124; found, 341.1128.

cis-2,5-diphenyl-2-(trifluoromethyl)-1-oxaspiro[2.3]hexane (24)

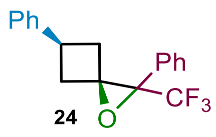

24

Prepared following **General Procedure A**, using 2,2,2-trifluoroacetophenone (42.0 µL, 52.2 mg, 0.300 mmol, 1.00 equiv), dissolved in THF (0.900 mL), as starting material. Biotage Isolera™ flash purification on silica gel (Sfär High Capacity 10 g silica cartridge), eluting with EtOAc/hexane (0 – 5%, v/v) gave epoxide **24** (43.4 mg, 48%) as a white amorphous solid.

R*f* = 0.22 (2:98 EtOAc/hexane, UV, cerium molybdate)

NMR Spectroscopy (*see spectra*):

^{1}H NMR (500 MHz, $CDCl_3$): δ_H 7.50 – 7.47 (m, 2H), 7.44 – 7.39 (m, 3H), 7.34 – 7.31 (m, 2H), 7.26 – 7.21 (m, 3H), 3.37 (apparent p, J = 8.8 Hz, 1H), 3.11 – 3.06 (m, 1H), 2.89 (dd, J = 13.7, 8.9 Hz, 1H), 2.42 (dd, J = 13.5, 8.7 Hz, 1H), 2.31 (ddd, J = 13.6, 8.8, 4.1 Hz, 1H) ppm;

^{13}C NMR (126 MHz, $CDCl_3$): δ_C 144.0, 130.8, 129.3, 128.7, 128.7, 127.2, 127.2, 126.6, 126.6, 124.0 (q, $^1J_{CF}$ = 279.1 Hz), 66.3, 64.0 (q, $^2J_{CF}$ = 35.9 Hz), 37.5, 36.6, 31.0 ppm;

^{19}F NMR (377 MHz, $CDCl_3$): δ_F –69.7 ppm.

IR (film): ν_{max} 3030, 2988, 2939, 1497, 1331, 1158, 1124 cm^{-1}.

HRMS (ESI$^+$): m/z calc'd for $C_{18}H_{15}F_3ONa$ [M+Na]$^+$, 327.0967; found, 327.0981.

3-(_cis_-2-methyl-5-phenyl-1-oxaspiro[2.3]hexan-2-yl)pyridine (25)

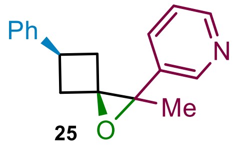

25

Prepared following **General Procedure A**, using 3-acetylpyridine (33.0 µL, 36.3 mg, 0.300 mmol, 1.00 equiv), dissolved in THF (0.900 mL), as starting material. Biotage Isolera™ flash purification on silica gel (Sfär High Capacity 10 g silica cartridge), eluting with acetone/pentane (5 – 40%, v/v) gave epoxide **25** (41.2 mg, 55%) as a white amorphous solid.

R*f* = 0.12 (20:80 EtOAc/hexane, UV, cerium molybdate)

NMR Spectroscopy (*see spectra*):

^{1}H NMR (400 MHz, $CDCl_3$): δ_H 8.65 – 8.56 (m, 2H), 7.67 (d, *J* = 7.7 Hz, 1H), 7.36 – 7.30 (m, 3H), 7.26 – 7.18 (m, 3H), 3.23 (apparent p, *J* = 8.7 Hz, 1H), 2.88 (ddd, *J* = 12.1, 8.3, 3.6 Hz, 1H), 2.66 (dd, *J* = 12.9, 8.9 Hz, 1H), 2.52 (dd, *J* = 12.9, 8.7 Hz, 1H), 2.21 (ddd, *J* = 12.8, 8.6, 4.3 Hz, 1H), 1.73 (s, 3H) ppm;

^{13}C NMR (101 MHz, $CDCl_3$): δ_C 147.6, 147.0, 144.3, 136.5, 134.8, 128.6, 126.6, 126.5, 123.5, 67.7, 62.1, 37.0, 36.8, 31.0, 19.5 ppm.

IR (film): ν_{max} 3028, 2970, 2930, 1495, 1454, 1422, 1379, 1080, 1022 cm^{-1}.

HRMS (ESI^+): m/z calc'd for $C_{17}H_{18}NO$ $[M+H]^+$, 252.1383; found, 252.1387.

2-chloro-4-(*cis*-2-methyl-5-phenyl-1-oxaspiro[2.3]hexan-2-yl)pyridine (26)

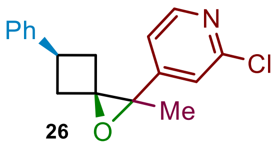

26

Prepared following **General Procedure A**, using 4-acetyl-2-chloropyridine (46.7 mg, 0.300 mmol, 1.00 equiv), dissolved in THF (0.900 mL), as starting material. Biotage Isolera™ flash purification on silica gel (Sfär High Capacity 10 g silica cartridge), eluting with acetone/pentane (1 – 10%, v/v) gave epoxide **26** (55.3 mg, 65%) as a white crystalline solid.

m.p.: = 88 – 89 °C (DCM/hexane)

R*f* = 0.33 (20:80 acetone/hexane, UV, cerium molybdate)

NMR Spectroscopy (*see spectra*):

^{1}H NMR (400 MHz, $CDCl_3$): δ_H 8.36 (dd, *J* = 5.2, 0.7 Hz, 1H), 7.35 – 7.20 (m, 6H), 7.15 (dd, *J* = 5.2, 1.5 Hz, 1H), 3.23 (apparent p, *J* = 8.8 Hz, 1H), 2.87 (dddd, *J* = 13.0, 8.6, 4.2, 1.4 Hz, 1H), 2.66 (ddd, *J* = 13.1, 9.0, 1.3 Hz, 1H), 2.53 (ddd, *J* = 13.0, 8.9, 1.3 Hz, 1H), 2.19 (dddd, *J* = 12.9, 8.6, 4.2, 1.6 Hz, 1H), 1.68 (s, 3H) ppm;

^{13}C NMR (101 MHz, $CDCl_3$): δ_C 152.9, 152.0, 149.6, 144.2, 128.7, 126.6, 121.7, 119.9, 67.9, 62.4, 36.9, 36.7, 31.0, 18.8 ppm.

IR (film): ν_{max} 3028, 2972, 2931, 1593, 1543,1367, 1129, 1079 cm^{-1}.

HRMS (ESI^+): m/z calc'd for $C_{17}H_{17}ClNO$ $[M+H]^+$, 286.0993; found, 286.0999.

tert-butyl cis-2-phenyl-11-oxa-8-azadispiro[3.0.5⁵.1⁴]undecane-8-carboxylate (27)

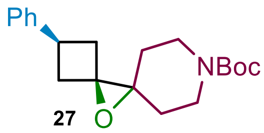

27

Prepared following **General Procedure A**, using 1-Boc-4-piperidone (59.8 mg, 0.300 mmol, 1.00 equiv), dissolved in THF (0.900 mL), as starting material. Biotage Isolera™ flash purification on silica gel (Sfär High Capacity 10 g silica cartridge), eluting with EtOAc/hexane (3 – 30%, v/v) gave epoxide **27** (69.5 mg, 70%) as a white crystalline solid.

m.p.: = 120 – 121 °C (DCM)

R*f* = 0.19 (15:85 EtOAc/hexane, UV, cerium molybdate)

NMR Spectroscopy (*see spectra*):

> **^{1}H NMR** (400 MHz, $CDCl_3$): δ_H 7.35 – 7.28 (m, 4H), 7.24 – 7.20 (m, 1H), 3.77 – 3.71 (m, 2H), 3.44 (ddd, *J* = 13.2, 9.3, 3.7 Hz, 2H), 3.29 (apparent p, *J* = 8.8 Hz, 1H), 2.75 – 2.63 (m, 2H), 2.61 – 2.48 (m, 2H), 1.75 (ddd, *J* = 13.7, 9.3, 4.5 Hz, 2H), 1.49 (s, 9H), 1.51 – 1.42 (m, 2H) ppm;

> **^{13}C NMR** (101 MHz, $CDCl_3$): δ_C 154.9, 144.5, 128.6, 126.6, 126.4, 79.9,65.3, 62.9, 42.4 (br s, CNBoc), 36.6, 31.6, 30.8, 28.6 ppm.

IR (film): ν_{max} 2973, 2929, 1694 (C=O), 1416, 1365, 1239, 1166, 1128 cm^{-1}.

HRMS (ESI^+): m/z calc'd for $C_{20}H_{27}NO_3Na$ $[M+Na]^+$, 352.188314; found, 352.188504.

cis-2-phenyl-9-oxadispiro[3.0.3⁵.1⁴]nonane (28)

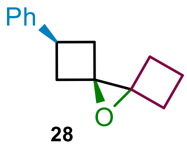

28

Prepared following **General Procedure A**, using cyclobutanone (22.4 µL, 21.0 mg, 0.300 mmol, 1.00 equiv), dissolved in THF (0.900 mL), as starting material. The reaction was performed with a different catalyst loading: $Pd(dba)_2$ (17.3 mg, 30.1 µmol, 10 mol%) and dippf (15.0 mg, 36.0 µmol, 12 mol%) in THF (1.80 mL). Biotage Isolera™ flash purification on silica gel (Sfär 25 g silica cartridge), eluting with EtOAc/hexane (0 – 6%, v/v) gave epoxide **28** (25.2 mg, 42%) as a colourless oil.

R*f* = 0.15 (3:97 EtOAc/hexane, UV, cerium molybdate)

NMR Spectroscopy (*see spectra*):

> **^{1}H NMR** (400 MHz, $CDCl_3$): δ_H 7.36 – 7.29 (m, 4H), 7.24 – 7.20 (m, 1H), 3.30 (apparent p, *J* = 8.7 Hz, 1H), 2.64 – 2.52 (m, 4H), 2.51 – 2.42 (m, 2H), 2.29 – 2.22 (m, 2H), 1.98 – 1.88 (m, 1H), 1.78 (dp, *J* = 11.2, 8.9 Hz, 1H) ppm;

^{13}**C NMR** (101 MHz, $CDCl_3$): δ_C 144.7, 128.6, 126.7, 126.4, 66.6, 63.5, 36.8, 30.9, 29.1, 12.2 ppm.

IR (film): ν_{max} 3027, 2965, 2928, 1494, 1454, 1236, 1105, 1061, 1037 cm^{-1}.

HRMS (ESI$^+$): m/z calc'd for $C_{14}H_{16}ONa$ $[M+Na]^+$, 223.1093; found, 223.1093.

***tert*-butyl *cis*-7-phenyl-9-oxa-2-azadispiro[3.0.3^5.1^4]nonane-2-carboxylate (29)**

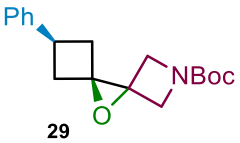

29

Prepared following **General Procedure A**, using 1-Boc-3-azetidinone (51.4 mg, 0.300 mmol, 1.00 equiv), dissolved in THF (0.900 mL), as starting material. The reaction was performed with a different catalyst loading: $Pd(dba)_2$ (17.3 mg, 30.1 µmol, 10 mol%) and dippf (15.0 mg, 36.0 µmol, 12 mol%) in THF (1.80 mL). Biotage Isolera™ flash purification on silica gel (Sfär 25 g silica cartridge), eluting with EtOAc/hexane (3 – 30%, v/v) gave epoxide **29** (28.0 mg, 31%) as a pale-yellow oil.

R*f* = 0.19 (15:85 EtOAc/hexane, UV, cerium molybdate)

NMR Spectroscopy (*see spectra*):

^{1}**H NMR** (400 MHz, $CDCl_3$): δ_H 7.37 – 7.27 (m, 4H), 7.26 – 7.19 (m, 1H), 4.19 – 4.12 (m, 4H), 3.37 (apparent p, *J* = 8.8 Hz, 1H), 2.62 (apparent d, *J* = 8.7 Hz, 4H), 1.48 (s, 9H) ppm;

^{13}**C NMR** (101 MHz, $CDCl_3$): δ_C 156.3, 144.0, 128.7, 126.6, 80.2, 62.7, 62.4, 55.9, 36.6 (br s, CNBoc), 30.8, 28.5 ppm.

IR (film): ν_{max} 2975, 2932, 1702 (C=O), 1391, 1134 cm^{-1}.

HRMS (Nanospray$^+$): m/z calc'd for $C_{18}H_{23}NO_3Na$ $[M+Na]^+$, 324.1576; found, 324.1588.

***cis*-2-ethyl-5-(4-methoxyphenyl)-2-phenyl-1-oxaspiro[2.3]hexane (30)**

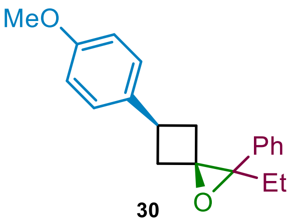

30

Prepared following **General Procedure B**, using 4-methoxyphenyl trifluoromethanesulfonate (70.0 µL, 99.1 mg, 0.387 mmol, 1.29 equiv). Biotage Isolera™ flash purification on silica gel (Sfär High Capacity 10 g silica cartridge), eluting with EtOAc/hexane (1 – 10%, v/v) gave epoxide **30** (66.5 mg, 75%) as a colourless oil.

R*f* = 0.22 (5:95 EtOAc/hexane, UV, cerium molybdate)

NMR Spectroscopy (see spectra):

^{1}**H NMR** (400 MHz, $CDCl_3$): δ_H 7.32 – 7.18 (m, 5H), 7.13 – 7.10 (m, 2H), 6.81 – 6.77 (m, 2H), 3.72 (s, 3H), 3.11 (apparent p, *J* = 8.7 Hz, 1H), 2.80 (dddd, *J* = 12.7, 8.4, 4.3, 1.3 Hz, 1H), 2.51 (ddd, *J* = 12.7, 9.0, 1.3 Hz, 1H), 2.30 (ddd, *J* = 12.8, 9.0, 1.3 Hz, 1H), 2.26 – 2.19 (m, 1H), 2.10 (dddd, *J* = 12.8, 8.5, 4.3, 1.3 Hz, 1H), 1.51 (dq, *J* = 14.6, 7.4 Hz, 1H), 0.91 (t, *J* = 7.4 Hz, 3H) ppm;

^{13}**C NMR** (101 MHz, $CDCl_3$): δ_C 158.1, 138.4, 137.0, 128.2, 127.6, 127.2, 126.6, 113.9, 68.1, 67.4, 55.4, 37.5, 30.7, 26.5, 9.3 ppm.

IR (film): ν_{max} 2970, 2934, 1513, 1246 (C-O), 1178, 1066, 1037 cm^{-1}.

HRMS (ESI$^+$): m/z calc'd for $C_{20}H_{22}O_2Na$ [M+Na]$^+$, 317.151201; found, 317.152069.

2-(*cis*-2-ethyl-2-phenyl-1-oxaspiro[2.3]hexan-5-yl)pyridine (31)

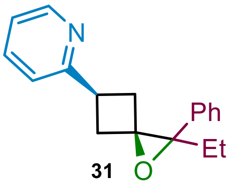

31

Prepared following **General Procedure B**, using 2-pyridyl trifluoromethanesulfonate (60.0 µL, 88.6 mg, 0.390 mmol, 1.30 equiv). Biotage Isolera™ flash purification on silica gel (Sfär High Capacity 10 g silica cartridge), eluting with acetone/hexane (3 – 30%, v/v) gave epoxide **31** (46.5 mg, 58%) as a colourless oil.

R*f* = 0.22 (15:85 EtOAc/hexane, UV, cerium molybdate)

NMR Spectroscopy (see spectra):

^{1}**H NMR** (400 MHz, $CDCl_3$): δ_H 8.55 (ddd, *J* = 4.9, 1.8, 1.0 Hz, 1H), 7.61 (td, *J* = 7.6, 1.8 Hz, 1H), 7.37 – 7.30 (m, 4H), 7.30 – 7.22 (m, 1H), 7.22 – 7.19 (m, 1H), 7.12 (ddd, *J* = 7.5, 4.9, 1.2 Hz, 1H), 3.36 (apparent p, *J* = 8.7 Hz, 1H), 2.91 – 2.85 (m, 1H), 2.82 (ddd, *J* = 12.7, 8.8, 0.8 Hz, 1H), 2.58 (ddd, *J* = 12.8, 8.8, 1.2 Hz, 1H), 2.30 (dq, *J* = 14.1, 7.5 Hz, 1H), 2.21 – 2.15 (m, 1H), 1.58 (dq, *J* = 14.6, 7.4 Hz, 1H), 0.97 (t, *J* = 7.4 Hz, 3H) ppm;

^{13}**C NMR** (101 MHz, $CDCl_3$): δ_C 163.4, 149.3, 138.4, 136.6, 128.2, 127.2, 126.7, 121.5, 121.3, 67.9, 67.5, 36.0, 36.0, 33.2, 26.5, 9.3 ppm.

IR (film): ν_{max} 3061, 2971, 2934, 1590, 1569, 1497, 1473, 1434, 1297, 1148, 1066 cm^{-1}.

HRMS (ESI$^+$): m/z calc'd for $C_{18}H_{20}NO$ [M+H]$^+$, 266.153941; found, 266.153815.

8-(*cis*-2-phenyl-1-oxaspiro[2.3]hexan-5-yl)quinoline (32)

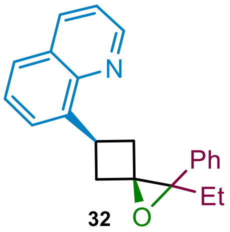

32

Prepared following **General Procedure B**, using 8-quinolinyl trifluoromethanesulfonate (**S32**) (108.1 mg, 0.390 mmol, 1.30 equiv). Biotage Isolera™ flash purification on silica gel (Sfär High Capacity 10 g silica cartridge), eluting with acetone/hexane (1 – 10%, v/v) gave a mixture, which was further purified by flash column chromatography (8 g SiO_2), eluting with EtOAc/hexane (10%, v/v), to obtain epoxide **32** (82.3 mg, 87%) as a pale-yellow oil.

R*f* = 0.20 (5:95 acetone/hexane, UV, cerium molybdate)

R*f* = 0.19 (10:90 EtOAc/hexane, UV, cerium molybdate)

NMR Spectroscopy (*see spectra*):

> **^{1}H NMR** (400 MHz, $CDCl_3$): δ_H 8.80 (dd, *J* = 4.2, 1.8 Hz, 1H), 8.04 (dd, *J* = 8.3, 1.8 Hz, 1H), 7.63 – 7.58 (m, 2H), 7.45 (dd, *J* = 8.2, 7.1 Hz, 1H), 7.31 – 7.26 (m, 5H), 7.21 – 7.17 (m, 1H), 4.27 (apparent p, *J* = 8.9 Hz, 1H), 3.03 (dddd, *J* = 12.8, 8.6, 4.2, 1.3 Hz, 1H), 2.60 (ddd, *J* = 12.7, 9.4, 1.3 Hz, 1H), 2.49 (ddd, *J* = 12.8, 9.4, 1.3 Hz, 1H), 2.34 – 2.24 (m, 2H), 1.55 (dq, *J* = 14.6, 7.4 Hz, 1H), 0.90 (t, *J* = 7.4 Hz, 3H) ppm;
>
> **^{13}C NMR** (101 MHz, $CDCl_3$): δ_C 149.3, 146.7, 143.0, 138.6, 136.4, 128.5, 128.2, 127.1, 126.8, 126.5, 126.3, 126.0, 121.1, 68.3, 68.1, 37.4, 36.3, 27.1, 26.6, 9.3 ppm.

IR (film): ν_{max} 2971, 2934, 1597, 1497, 1465, 1373, 1298, 1179, 1089 cm^{-1}.

HRMS (ESI^+): m/z calc'd for $C_{22}H_{22}NO$ $[M+H]^+$, 316.1696; found, 316.1689.

***cis*-5-(cyclohex-1-en-1-yl)-2-ethyl-2-phenyl-1-oxaspiro[2.3]hexane (33)**

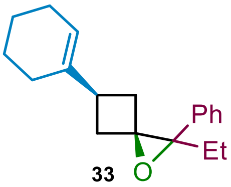

33

Prepared following **General Procedure B**, using 1-cyclohexenyl trifluoromethanesulfonate (68.0 µL, 89.4 mg, 0.388 mmol, 1.29 equiv). Biotage Isolera™ flash purification on silica gel (Sfär High Capacity 10 g silica cartridge), eluting with EtOAc/hexane (0 – 4%, v/v) gave a mixture, which was further purified by flash column chromatography (8 g SiO_2), eluting with toluene/hexane (10%, v/v), to obtain epoxide **33** (51.4 mg, 64%) as a colourless oil.

R*f* = 0.19 (2:98 EtOAc/hexane, UV, cerium molybdate)

R$_f$ = 0.12 (10:90 toluene/hexane, UV, cerium molybdate)

NMR Spectroscopy (*see spectra*):

^{1}H NMR (400 MHz, $CDCl_3$): δ_H 7.36 – 7.31 (m, 2H), 7.29 – 7.23 (m, 3H), 5.45 – 5.42 (m, 1H), 2.53 – 2.44 (m, 2H), 2.41 – 2.33 (m, 1H), 2.23 (dq, J = 14.1, 7.4 Hz, 1H), 2.17 – 2.09 (m, 1H), 2.00 (dtq, J = 8.2, 4.0, 1.9 Hz, 2H), 1.89 (tq, J = 6.3, 1.8 Hz, 2H), 1.84 – 1.78 (m, 1H), 1.64 – 1.49 (m, 5H), 0.93 (t, J = 7.5 Hz, 3H) ppm;

^{13}C NMR (101 MHz, $CDCl_3$): δ_C 139.3, 138.6, 128.1, 127.1, 126.7, 119.9, 68.0, 67.6, 33.9, 33.7, 32.8, 26.6, 26.2, 25.2, 22.9, 22.7, 9.3 ppm.

IR (film): ν_{max} 2969, 2926, 2834, 1496, 1446, 1296, 1133, 1072, 1030 cm^{-1}.

HRMS (ESI$^+$): m/z calc'd for $C_{19}H_{25}O$ [M+H]$^+$, 269.189992; found, 269.190161.

4,4,5,5-tetramethyl-2-(4-(*cis*-2-phenyl-1-oxaspiro[2.3]hexan-5-yl)phenyl)-1,3,2-dioxaborolane (34)

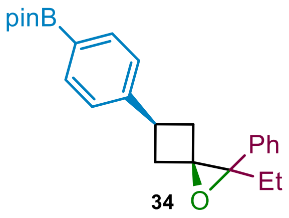

34

Prepared following **General Procedure B**, using 4-(4,4,5,5-tetramethyl-1,3,2-dioxaborolan-2-yl)phenyl trifluoromethanesulfonate **(S34)** (137.3 mg, 0.390 mmol, 1.30 equiv). The reaction was performed with a different catalyst loading: Pd(dba)$_2$ (17.3 mg, 30.1 µmol, 10 mol%) and dippf (15.0 mg, 36.0 µmol, 12 mol%) in THF (1.80 mL) for the second step of the reaction. Biotage Isolera™ flash purification on silica gel (Sfär High Capacity 10 g silica cartridge), eluting with EtOAc/hexane (2%, v/v) gave epoxide **34** (63.5 mg, 54%) as a white crystalline solid.

m.p.: = 113 – 114 °C (hexane)

R$_f$ = 0.23 (2:98 EtOAc/hexane, UV, cerium molybdate)

NMR Spectroscopy (*see spectra*):

^{1}H NMR (400 MHz, $CDCl_3$): δ_H 7.73 – 7.71 (m, 2H), 7.34 – 7.21 (m, 7H), 3.19 (apparent p, *J* = 8.7 Hz, 1H), 2.84 (dddd, *J* = 12.7, 8.5, 4.2, 1.3 Hz, 1H), 2.59 (ddd, *J* = 12.8, 9.1, 1.3 Hz, 1H), 2.37 (ddd, *J* = 12.9, 9.0, 1.3 Hz, 1H), 2.26 (dq, *J* = 14.9, 7.5 Hz, 1H), 2.14 (dddd, *J* = 12.8, 8.5, 4.2, 1.3 Hz, 1H), 1.58 – 1.49 (m, 1H), 1.29 (s, 12H), 0.93 (t, *J* = 7.4 Hz, 3H) ppm;

^{13}C NMR (101 MHz, $CDCl_3$): δ_C 148.2, 138.3, 135.1, 128.3, 127.2, 126.6, 126.1, 83.8, 68.1, 67.4, 37.1, 31.5, 26.5, 25.0, 9.3 ppm. The carbon attached to boron was not observed due to quadrupolar relaxation.

^{11}B NMR (128 MHz, $CDCl_3$): δ_B 32.3 ppm

IR (film): ν_{max} 2974, 2932, 1610, 1398, 1358, 1320, 1270, 1143, 1089 cm^{-1}.

HRMS (ESI$^+$): m/z calc'd for $C_{25}H_{31}BO_3Na$ [M+Na]$^+$, 413.226296; found, 413.226823.

(8*R*,9*S*,13*S*,14*S*)-3-(*cis*-2-ethyl-2-phenyl-1-oxaspiro[2.3]hexan-5-yl)-13-methyl-6,7,8,9,11,12,13,14,15,16-decahydro-17*H*-cyclopenta[a]phenanthren-17-one (35)

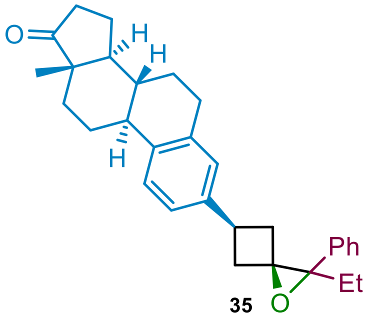

35

Prepared following **General Procedure B**, using 3-(trifluoromethanesulfonyl)estrone (**S35**) (157.0 mg, 0.390 mmol, 1.30 equiv). Biotage Isolera™ flash purification on silica gel (Sfär High Capacity 10 g silica cartridge), eluting with EtOAc/hexane (3 – 30%, v/v) gave epoxide **35** (109.6 mg, 83%) as a colourless oil.

R*f* = 0.20 (15:85 EtOAc/hexane, UV, cerium molybdate)

NMR Spectroscopy (*see spectra*):

> **^{1}H NMR** (400 MHz, $CDCl_3$): δ_H 7.39 – 7.24 (m, 6H), 7.08 – 7.02 (m, 2H), 3.17 (apparent p, *J* = 8.7 Hz, 1H), 2.94 – 2.84 (m, 3H), 2.62 (ddd, *J* = 12.8, 9.0, 1.3 Hz, 1H), 2.51 (dd, *J* = 18.9, 8.4 Hz, 1H), 2.46 – 2.38 (m, 2H), 2.35 – 2.26 (m, 2H), 2.21 – 1.94 (m, 5H), 1.71 – 1.38 (m, 7H), 0.97 (t, *J* = 7.4 Hz, 3H), 0.91 (s, 3H) ppm;

> **^{13}C NMR** (101 MHz, $CDCl_3$): δ_C 220.9, 142.4, 138.4, 137.8, 136.6, 128.2, 127.3, 127.2, 126.6, 125.6, 124.2, 68.1, 67.4, 50.6, 48.1, 44.4, 38.3, 37.3, 37.2, 36.0, 31.7, 30.9, 29.6, 26.7, 26.5, 25.9, 21.7, 14.0, 9.3 ppm.

IR (film): ν_{max} 2967, 2929, 2874, 1737 (C=O), 1499, 1453, 1374, 1297, 1257, 1084, 1055 cm^{-1}.

HRMS (ESI$^+$): m/z calc'd for $C_{31}H_{36}O_2Na$ [M+Na]$^+$, 463.2608; found, 463.2596.

cis-2,5-diphenyl-1-oxaspiro[2.3]hexane (36)

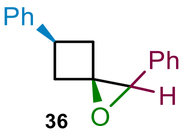

36

tert-Butyl lithium (1.7 M in pentane, 1.60 mL, 2.72 mmol, 1.36 equiv)[A] was added dropwise[B] to a solution of BCB-sulfoxide **10** (519.2 mg, 2.70 mmol, 1.35 equiv) in THF[C] (20.0 mL) at –95 °C (acetone/liquid nitrogen) and allowed to stir for 2 min,[D] before a solution of benzaldehyde (204 µL, 212 mg, 2.00 mmol, 1.00 equiv) in THF[C] (6.00 mL) was added dropwise.[B]. Dry ice was added to the cooling bath and stirring continued for 1 h. After removing the cooling bath toluene[C] (46 mL) and phenyl trifluoromethanesulfonate (421 µL, 588 mg, 2.60 mmol, 1.30 equiv) were added, followed by.a solution[E] of Pd(dba)$_2$ (57.5 mg, 100 µmol, 5.0 mol%) and dippf (50.2 mg, 120 µmol, 6.0 mol%) in toluene[C] (6.00 mL) (pre-mixed under nitrogen for 1 h). The flask was sealed and heated at 60 °C (oil bath) for 18 h.[F] Water was added to quench the reaction, then Et$_2$O was added, and the phases were separated. The aqueous phase was extracted with Et$_2$O (3 x) and the combined organic phases were dried over MgSO$_4$, filtered, and concentrated under reduced pressure. Biotage Isolera™ flash purification on silica gel (Sfär High Capacity 50 g silica cartridge), eluting with EtOAc/hexane (0 – 6%, v/v) gave **36** (279.5 mg, 59%) as a pale-yellow oil which solidified in the freezer.

Notes: see *2.3.4. General Notes*

m.p.: = 44 – 45 °C

R*f* = 0.21 (3:97 EtOAc/hexane, UV, cerium molybdate)

NMR Spectroscopy (*see spectra*):

^{1}H NMR (400 MHz, CDCl$_3$): δ_H 7.40 – 7.20 (m, 10H), 3.99 (s, 1H), 3.26 (apparent p, *J* = 8.8 Hz, 1H), 2.87 (dddd, *J* = 12.5, 8.3, 4.1, 1.2 Hz, 1H), 2.78 (ddd, *J* = 12.4, 9.1, 1.0 Hz, 1H), 2.63 (ddd, *J* = 12.6, 9.0, 0.9 Hz, 1H), 2.44 (dddd, *J* = 12.7, 8.5, 4.1, 1.1 Hz, 1H) ppm;

^{13}C NMR (101 MHz, CDCl$_3$): δ_C 144.6, 136.7, 128.6, 128.4, 128.0, 126.7, 126.4, 126.3, 63.9, 62.4, 39.2, 36.2, 31.1 ppm.

IR (film): ν_{max} 3026, 2972, 2930, 1603, 1495, 1453, 1028 cm^{-1}.

HRMS (ESI$^+$): m/z calc'd for $C_{17}H_{16}ONa$ [M+Na]$^+$, 259.109336; found, 259.110456.

***cis*-2-(naphthalen-1-yl)-5-phenyl-1-oxaspiro[2.3]hexane (37)**

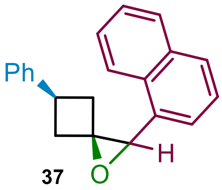

37

Prepared following **General Procedure C**, using 1-napthaldehyde (40.7 µL, 46.9 mg, 0.300 mmol, 1.00 equiv), dissolved in THF (0.900 mL), as starting material. Biotage Isolera™ flash purification on silica gel (Sfär High Capacity 10 g silica cartridge), eluting with EtOAc/hexane (0 – 6%, v/v) gave epoxide **37** (59.1 mg, 69%) as a white crystalline solid.

m.p.: = 82 – 83 °C (hexane)

R_f = 0.16 (3:97 EtOAc/hexane, UV, cerium molybdate)

NMR Spectroscopy (*see spectra*):

> **^{1}H NMR** (400 MHz, $CDCl_3$): δ_H 8.15 – 8.13 (m, 1H), 7.96 – 7.93 (m, 1H), 7.84 – 7.81 (m, 1H), 7.63 (ddd, *J* = 8.4, 6.8, 1.5 Hz, 1H), 7.57 (ddd, *J* = 8.1, 6.8, 1.3 Hz, 1H), 7.52 – 7.48 (m, 1H), 7.41 (dt, *J* = 7.1, 1.1 Hz, 1H), 7.34 – 7.30 (m, 2H), 7.27 – 7.19 (m, 3H), 4.61 (s, 1H), 3.32 (apparent p, *J* = 8.8 Hz, 1H), 3.06 (dddd, *J* = 12.5, 8.2, 4.4, 1.3 Hz, 1H), 2.91 (ddd, *J* = 12.6, 9.2, 1.1 Hz, 1H), 2.51 (dd, *J* = 12.7, 9.2 Hz, 1H), 2.12 (dddd, *J* = 12.7, 8.4, 4.4, 1.3 Hz, 1H) ppm;

> **^{13}C NMR** (101 MHz, $CDCl_3$): δ_C 144.5, 133.4, 132.6, 131.4, 129.1, 128.6, 128.0, 126.6, 126.5, 126.4, 126.0, 125.7, 123.1, 122.7, 63.8, 60.7, 39.3, 36.3, 31.7 ppm.

IR (film): ν_{max} 3057, 2970, 2930, 1597, 1510, 1495, 1309, 1172 cm^{-1}.

HRMS (ESI^+): m/z calc'd for $C_{21}H_{18}ONa$ $[M+Na]^+$, 309.1250; found, 309.1248.

cis-2-(4-chlorophenyl)-5-phenyl-1-oxaspiro[2.3]hexane (38)

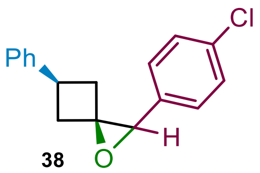

38

Prepared following **General Procedure C**, using 4-chlorobenzaldehyde (42.2 mg, 0.300 mmol, 1.00 equiv), dissolved in THF (0.900 mL), as starting material. Biotage Isolera™ flash purification on silica gel (Sfär High Capacity 10 g silica cartridge), eluting with EtOAc/hexane (0 – 5%, v/v) gave epoxide **38** (61.3 mg, 75%) as a white amorphous solid.

R*f* = 0.19 (3:97 EtOAc/hexane, UV, cerium molybdate)

NMR Spectroscopy (*see spectra*):

^{1}H NMR (400 MHz, $CDCl_3$): δ_H 7.37 – 7.31 (m, 4H), 7.29 – 7.26 (m, 2H), 7.25 – 7.21 (m, 1H), 7.19 – 7.15 (m, 2H), 3.97 (s, 1H), 3.26 (apparent p, *J* = 8.8 Hz, 1H), 2.87 (dddd, *J* = 12.4, 8.3, 4.1, 1.3 Hz, 1H), 2.78 (ddd, *J* = 12.6, 9.2, 1.0 Hz, 1H), 2.61 (ddd, *J* = 12.6, 9.0, 0.9 Hz, 1H), 2.39 (dddd, *J* = 12.7, 8.5, 4.1, 1.1 Hz, 1H) ppm;

^{13}C NMR (101 MHz, $CDCl_3$): δ_C 144.4, 135.3, 133.9, 128.6, 127.6, 126.7, 126.5, 64.0, 61.7, 39.1, 36.1, 31.1 ppm.

IR (film): ν_{max} 3026, 2974, 2931, 1493, 1090, 1014 cm^{-1}.

HRMS (EI^+): m/z calc'd for $C_{17}H_{15}OCl$ $[M]^+$, 270.0806; found, 270.0807.

cis-5-phenyl-1-oxaspiro[2.3]hexan-2-yl)pyridine (39)

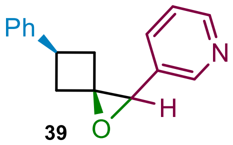

39

Prepared following **General Procedure C**, using 3-pyridinecarboxaldehyde (28.1 µL, 32.1 mg, 0.300 mmol, 1.00 equiv), dissolved in THF (0.900 mL), as starting material. Biotage Isolera™ flash purification on silica gel (Sfär High Capacity 10 g silica cartridge), eluting with acetone/pentane (5 – 25%, v/v) gave a mixture, which was further purified by Biotage Isolera™ (Sfär High Capacity 10 g silica cartridge), eluting with EtOAc/DCM (2 – 20%, v/v), to obtain epoxide **39** (41.6 mg, 58%) as a pale-yellow amorphous solid.

R*f* = 0.21 (20:80 acetone/pentane, UV, cerium molybdate)

R*f* = 0.15 (10:90 EtOAc/DCM, UV, cerium molybdate)

NMR Spectroscopy (*see spectra*):

^{1}**H NMR** (400 MHz, $CDCl_3$): δ_H 8.59 – 8.57 (m, 2H), 7.55 – 7.52 (m, 1H), 7.37 – 7.28 (m, 3H), 7.26 – 7.24 (m, 2H), 7.23 – 7.18 (m, 1H), 4.02 (s, 1H), 3.27 (apparent p, *J* = 8.8 Hz, 1H), 2.88 (dddd, *J* = 12.5, 8.4, 4.0, 1.3 Hz, 1H), 2.79 (ddd, *J* = 12.6, 9.2, 1.0 Hz, 1H), 2.62 (ddd, *J* = 12.7, 9.1, 1.1 Hz, 1H), 2.36 (dddd, *J* = 12.7, 8.6, 4.1, 1.2 Hz, 1H) ppm;

^{13}**C NMR** (101 MHz, $CDCl_3$): δ_C 148.4, 147.4, 144.1, 134.5, 133.1, 128.6, 126.7, 126.5, 123.6, 64.4, 59.9, 39.0, 35.9, 31.0 ppm.

IR (film): ν_{max} 3027, 2973, 2930, 1494, 1307, 1170, 1025 cm^{-1}.

HRMS (ESI$^+$): m/z calc'd for $C_{16}H_{16}NO$ [M+H]$^+$, 238.122641; found, 238.123076.

cis-2-(tert-butyl)-5-phenyl-1-oxaspiro[2.3]hexane (40)

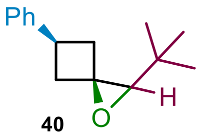

40

Prepared following **General Procedure C**, using pivalaldehyde (25.8 mg, 32.6 µL, 0.300 mmol, 1.00 equiv), dissolved in THF (0.900 mL), as starting material. Biotage Isolera™ flash purification on silica gel (Sfär High Capacity 10 g silica cartridge), eluting with EtOAc/hexane (0 – 4%, v/v) gave epoxide **40** (50.4 mg, 78%) as a colourless oil.

R*f* = 0.18 (2:98 EtOAc/hexane, UV, cerium molybdate)

NMR Spectroscopy (*see spectra*):

^{1}**H NMR** (400 MHz, $CDCl_3$): δ_H 7.36 – 7.30 (m, 4H), 7.24 – 7.19 (m, 1H), 3.35 (apparent p, *J* = 8.6 Hz, 1H), 2.97 (dddd, *J* = 13.0, 8.6, 3.8, 1.3 Hz, 1H), 2.76 (dddd, *J* = 12.5, 8.7, 3.8, 1.3 Hz, 1H), 2.69 (ddd, *J* = 13.0, 8.7, 0.7 Hz, 1H), 2.68 (s, 1H), 2.55 (ddd, *J* = 12.7, 8.7, 0.7 Hz, 1H), 1.00 (s, 9H) ppm;

^{13}**C NMR** (101 MHz, $CDCl_3$): δ_C 145.0, 128.6, 126.7, 126.3, 69.8, 61.2, 40.3, 38.2, 32.0, 31.9, 26.5 ppm.

IR (film): ν_{max} 2956, 2867, 1495, 1452, 1391, 1363, 1073, 1030 cm^{-1}.

HRMS (ESI$^+$): m/z calc'd for $C_{15}H_{20}ONa$ [M+Na]$^+$, 239.140636; found, 239.141213.

cis-2-phenethyl-5-phenyl-1-oxaspiro[2.3]hexane (41)

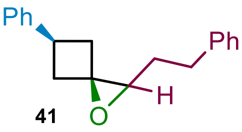

Prepared following **General Procedure C**, using hydrocinnamaldehyde (39.5 mg, 39.5 µL 0.300 mmol, 1.00 equiv), dissolved in THF (0.900 mL), as starting material. Biotage Isolera™ flash purification on silica gel (Sfär High Capacity 10 g silica cartridge), eluting with EtOAc/hexane (1 – 10%, v/v) gave epoxide **41** (52.4 mg, 66%) as a colourless oil.

R*f* = 0.17 (5:95 EtOAc/hexane, UV, cerium molybdate)

NMR Spectroscopy (*see spectra*):

> **^{1}H NMR** (400 MHz, $CDCl_3$): δ_H 7.34 – 7.29 (m, 4H), 7.26 – 7.18 (m, 6H), 3.05 – 2.96 (m, 2H), 2.91 (ddd, *J* = 13.8, 8.3, 5.5 Hz, 1H), 2.78 (dt, *J* = 13.8, 8.1 Hz, 1H), 2.62 – 2.58 (m, 2H), 2.46 – 2.43 (m, 2H), 1.89 (ddt, *J* = 13.8, 8.1, 5.8 Hz, 1H), 1.75 (dtd, *J* = 14.2, 8.2, 6.1 Hz, 1H) ppm;
>
> **^{13}C NMR** (101 MHz, $CDCl_3$): δ_C 144.6, 141.4, 128.7, 128.6, 126.7, 126.3, 126.3, 61.3, 61.2, 38.9, 36.4, 32.6, 32.2, 31.4 ppm.

IR (film): ν_{max} 3026, 2970, 2930, 1603, 1495, 1453, 1171, 1030 cm^{-1}.

HRMS (ESI$^+$): m/z calc'd for $C_{19}H_{20}ONa$ [M+Na]$^+$, 287.140636; found, 287.139887.

cis-2,5-diphenyl-1-tosyl-1-azaspiro[2.3]hexane (42)

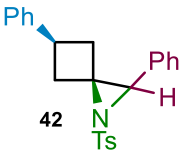

Prepared following **General Procedure C**, using *N*-Benzylidene-4-methylbenzensulfonamide (77.8 mg, 0.300 mmol, 1.00 equiv), dissolved in THF (0.900 mL), as starting material. Biotage Isolera™ flash purification on silica gel (Sfär 25 g silica cartridge), eluting with EtOAc/hexane (2 – 20%, v/v) gave aziridine **42** (87.1 mg, 75%) as a pale-yellow crystalline solid.

m.p.: = 123 – 124 °C (EtOAc/hexane)

R*f* = 0.20 (10:90 EtOAc/hexane, UV, cerium molybdate)

NMR Spectroscopy (*see spectra*):

> **^{1}H NMR** (400 MHz, $CDCl_3$): δ_H 7.89 – 7.87 (m, 2H), 7.34 – 7.27 (m, 7H), 7.22 – 7.17 (m, 5H), 4.05 (s, 1H), 3.41 (apparent p, *J* = 8.7 Hz, 1H), 3.31 (dd, *J* = 12.5, 9.8 Hz, 1H), 3.07 (dddd, *J* = 12.5, 8.1, 4.4, 1.3 Hz, 1H), 2.55 (ddd, *J* = 12.5, 9.2, 1.2 Hz, 1H), 2.42 (s, 3H), 2.25 (dddd, *J* = 12.7, 8.3, 4.4, 1.1 Hz, 1H) ppm;

^{13}C NMR (101 MHz, $CDCl_3$): δ_C 144.3, 144.0, 137.0, 134.2, 129.8, 128.6, 128.6, 128.1, 127.7, 127.0, 126.6, 126.5, 52.6, 51.1, 37.3, 35.6, 34.0, 21.8 ppm.

IR (film): ν_{max} 3028, 2981, 2973, 1599, 1496, 1454, 1405, 1321, 1154, 1091 cm^{-1}.

HRMS (ESI$^+$): m/z calc'd for $C_{24}H_{24}NO_2S$ [M+H]$^+$, 390.152226; found, 390.152032.

cis-2-(4-chlorophenyl)-5-phenyl-1-tosyl-1-azaspiro[2.3]hexane (43)

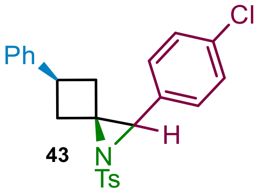

43

Prepared following **General Procedure C**, using *N*-(4-chlorobenzylidene)-4-methylbenzenesulfonamide (**S43**) (88.1 mg, 0.300 mmol, 1.00 equiv), dissolved in THF (1.80 mL), as starting material. Prior to addition of triflate and catalyst solution, toluene (8 mL) was added to the reaction mixture. Biotage Isolera™ flash purification on silica gel (Sfär High Capacity 10 g silica cartridge), eluting with EtOAc/hexane (3 – 30%, v/v) gave aziridine **43** (101.5 mg, 80%) as a yellow crystalline solid.

m.p.: = 108 – 109 °C (DCM/hexane)

R_f = 0.26 (15:85 EtOAc/hexane, UV, cerium molybdate)

NMR Spectroscopy (*see spectra*):

^{1}H NMR (400 MHz, $CDCl_3$): δ_H 7.87 – 7.85 (m, 2H), 7.32 – 7.27 (m, 6H), 7.23 – 7.17 (m, 3H), 7.13 – 7.10 (m, 2H), 4.00 (s, 1H), 3.40 (apparent p, *J* = 8.7 Hz, 1H), 3.29 (dd, *J* = 12.6, 9.3 Hz, 1H), 3.06 (dddd, *J* = 12.6, 8.2, 4.3, 1.3 Hz, 1H), 2.54 (ddd, *J* = 12.5, 9.2, 1.2 Hz, 1H), 2.42 (s, 3H), 2.22 (dddd, *J* = 12.7, 8.4, 4.6, 1.1 Hz, 1H) ppm;

^{13}C NMR (101 MHz, $CDCl_3$): δ_C 144.5, 143.8, 136.8, 134.0, 132.7, 129.8, 128.8, 128.6, 128.4, 127.6, 126.6, 126.5, 52.8, 50.3, 37.2, 35.5, 34.0, 21.8 ppm.

IR (film): ν_{max} 3027, 2981, 2938, 1599, 1494, 1423, 1324, 1155, 1090 cm^{-1}.

HRMS (ESI$^+$): m/z calc'd for $C_{24}H_{22}ClNO_2SNa$ [M+Na]$^+$, 446.091827; found, 446.091187.

cis-2-(4-nitrophenyl)-5-phenyl-1-tosyl-1-azaspiro[2.3]hexane (44)

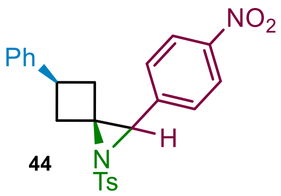

44

Prepared following **General Procedure C**, using 4-methyl-*N*-(4-nitrobenzylidene)benzenesulfonamide (**S44**) (91.3 mg, 0.300 mmol, 1.00 equiv), dissolved in THF (3.00 mL), as starting material. Prior to addition of triflate and catalyst solution, toluene (10 mL) was added to the black reaction mixture. Biotage Isolera™ flash purification on silica gel (Sfär 25 g silica cartridge), eluting with EtOAc/hexane (5 – 40%, v/v) gave aziridine **44** (80.6 mg, 62%) as a pale-yellow oil.

R_f = 0.23 (20:80 EtOAc/hexane, UV, cerium molybdate)

NMR Spectroscopy (*see spectra*):

^{1}H NMR (400 MHz, $CDCl_3$): δ_H 8.19 – 8.16 (m, 2H), 7.89 – 7.86 (m, 2H), 7.37 – 7.28 (m, 6H), 7.24 – 7.16 (m, 3H), 4.10 (s, 1H), 3.42 (apparent p, *J* = 8.7 Hz, 1H), 3.32 (dd, *J* = 12.8, 9.8 Hz, 1H), 3.10 (tdd, *J* = 8.3, 4.0, 1.3 Hz, 1H), 2.58 (ddd, *J* = 12.6, 9.2, 1.2 Hz, 1H), 2.43 (s, 3H), 2.17 (dddd, *J* = 12.8, 8.5, 4.4, 1.2 Hz, 1H) ppm;

^{13}C NMR (101 MHz, $CDCl_3$): δ_C 147.8, 144.8, 143.5, 141.7, 136.4, 129.9, 128.7, 127.9, 127.7, 126.7, 126.5, 123.9, 53.4, 49.8, 37.2, 35.5, 34.0, 21.8 ppm.

IR (film): ν_{max} 3028, 2937, 1600, 1519, 1495, 1345, 1320, 1156, 1091 cm^{-1}.

HRMS (ESI$^+$): m/z calc'd for $C_{24}H_{22}N_2O_4SNa$ $[M+Na]^+$, 457.119249; found, 457.119192.

cis-2-(4-methoxyphenyl)-5-phenyl-1-tosyl-1-azaspiro[2.3]hexane (45)

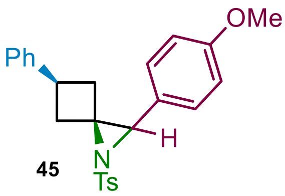

45

Prepared following **General Procedure C**, using *N*-Benzylidene-4-methylbenzensulfonamide (**S45**) (86.8 mg, 0.300 mmol, 1.00 equiv), dissolved in THF (0.900 mL), as starting material. Biotage Isolera™ flash purification on silica gel (Sfär 25 g silica cartridge), eluting with EtOAc/hexane (5 – 40%, v/v) gave aziridine **45** (115.0 mg, 91%) as a pale-yellow oil.

R_f = 0.26 (20:80 EtOAc/hexane, UV, cerium molybdate)

NMR Spectroscopy (*see spectra*):

^{1}H NMR (400 MHz, $CDCl_3$): δ_H 7.89 – 7.86 (m, 2H), 7.32 – 7.27 (m, 4H), 7.22 – 7.18 (m, 3H), 7.12 – 7.08 (m, 2H), 6.87 – 6.83 (m, 2H), 4.01 (s, 1H), 3.78 (s, 3H), 3.40 (apparent p, *J* = 8.7 Hz, 1H), 3.29 (dd, *J* = 12.7, 9.5 Hz, 1H), 3.05 (dddd, *J* = 12.6, 8.2, 4.4, 1.3 Hz, 1H), 2.55 (ddd, *J* = 12.5, 9.2, 1.2 Hz, 1H), 2.41 (s, 3H), 2.29 (dddd, *J* = 12.5, 8.3, 4.3, 1.1 Hz, 1H) ppm;

^{13}C NMR (101 MHz, $CDCl_3$): δ_C 159.6, 144.2, 144.0, 137.1, 129.8, 128.6, 128.2, 127.6, 126.6, 126.5, 126.1, 114.0, 55.4, 52.5, 50.9, 37.2, 35.6, 34.0, 21.7 ppm.

IR (film): ν_{max} 2935, 2837, 1613, 1515, 1320, 1248, 1154, 1091, 1031 cm^{-1}.

HRMS (ESI^+): m/z calc'd for $C_{25}H_{25}NO_3SNa$ $[M+Na]^+$, 442.144735; found, 442.144963.

cis-5-phenyl-2-(pyridin-3-yl)-1-tosyl-1-azaspiro[2.3]hexane (46)

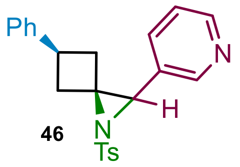

46

Prepared following **General Procedure C**, using 4-methyl-*N*-(pyridin-3-ylmethylene)benzenesulfonamide (**S46**) (78.1 mg, 0.300 mmol, 1.00 equiv), dissolved in THF (0.900 mL), as starting material. Biotage Isolera™ flash purification on silica gel (Sfär 25 g silica cartridge), eluting with EtOAc/hexane (7 – 50%, v/v) gave aziridine **46** (93.0 mg, 79%) as a pale-yellow amorphous solid.

R_f = 0.22 (30:70 EtOAc/hexane, UV, cerium molybdate)

NMR Spectroscopy (*see spectra*):

^{1}H NMR (400 MHz, $CDCl_3$): δ_H 8.53 – 8.49 (m, 2H), 7.88 – 7.86 (m, 2H), 7.44 – 7.42 (m, 1H), 7.32 – 7.28 (m, 4H), 7.25 – 7.18 (m, 4H), 4.04 (s, 1H), 3.43 (apparent p, *J* = 8.8 Hz, 1H), 3.32 (dd, *J* = 12.7, 9.4 Hz, 1H), 3.09 (dddd, *J* = 12.7, 8.2, 4.3, 1.3 Hz, 1H), 2.57 (ddd, *J* = 12.5, 9.2, 1.2 Hz, 1H), 2.42 (s, 3H), 2.23 (dddd, *J* = 12.8, 8.5, 4.3, 1.2 Hz, 1H) ppm;

^{13}C NMR (101 MHz, $CDCl_3$): δ_C 149.5, 148.9, 144.6, 143.6, 136.6, 134.4, 130.1, 129.9, 128.6, 127.7, 126.6, 126.5, 123.4, 52.8, 48.6, 37.1, 35.4, 33.9, 21.7 ppm.

IR (film): ν_{max} 3028, 2982, 2938, 1495, 1426, 1323, 1304, 1156, 1091 cm^{-1}

HRMS (ESI^+): m/z calc'd for $C_{23}H_{23}N_2O_2S$ $[M+H]^+$,391.1475; found, 391.1473.

cis-2-(tert-butyl)-5-phenyl-1-tosyl-1-azaspiro[2.3]hexane (47)

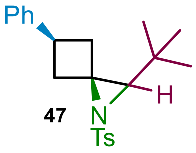

47

Prepared following **General Procedure C**, using *N*-(2,2-dimethylpropylidene)-4-methylbenzenesulfonamide (**S47**) (71.8 mg, 0.300 mmol, 1.00 equiv), dissolved in THF (0.900 mL), as starting material. Biotage Isolera™ flash purification on silica gel (Sfär 25 g silica cartridge), eluting with EtOAc/hexane (2 – 16%, v/v) gave aziridine **47** (98.0 mg, 88%) as a pale-yellow crystalline solid.

m.p.: = 84 – 85 °C (DCM)

R_f = 0.20 (8:92 EtOAc/hexane, UV, cerium molybdate)

NMR Spectroscopy (*see spectra*):

> **^{1}H NMR** (400 MHz, $CDCl_3$): δ_H 7.89 – 7.86 (m, 2H), 7.33 – 7.28 (m, 4H), 7.22 – 7.18 (m, 3H), 3.45 (apparent p, *J* = 9.0 Hz, 1H), 3.21 (ddd, *J* = 11.4, 9.2, 1.7 Hz, 1H), 2.91 – 2.86 (m, 1H), 2.85 – 2.80 (m, 1H), 2.72 (ddd, *J* = 11.9, 9.6, 1.7 Hz, 1H), 2.68 (s, 1H), 2.45 (s, 3H), 0.85 (s, 9H) ppm;
>
> **^{13}C NMR** (101 MHz, $CDCl_3$): δ_C 144.1, 144.1, 137.2, 129.6, 128.6, 128.1, 126.6, 126.5, 58.1, 50.1, 38.3, 37.3, 34.2, 31.5, 27.3, 21.8 ppm.

IR (film): ν_{max} 2955, 1599, 1495, 1422, 1315, 1155, 1092 cm^{-1}.

HRMS (ESI$^+$): m/z calc'd for $C_{22}H_{28}NO_2S$ [M+H]$^+$, 370.1835; found, 370.1830.

2.5. Derivatisation Reactions

cis-1-(azido(phenyl)methyl)-3-phenylcyclobutan-1-ol (48)[11]

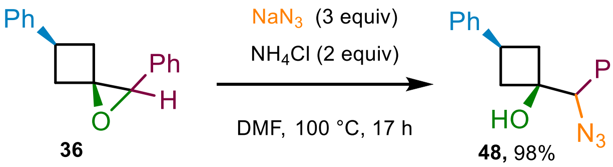

A flame dried microwave vial was charged with epoxide **36** (23.6 mg, 0.100 mmol, 1.00 equiv), NH_4Cl (10.7 mg, 0.200 mmol, 2.00 equiv), NaN_3 (39.6 mg, 0.300 mmol, 3.00 equiv) and DMF (1.00 mL). The vial was sealed and heated to 100 °C for 17 h. After cooling to room temperature, the resulting yellow suspension was quenched by addition of a saturated aqueous solution of $NaHCO_3$, then Et_2O was added, and the phases were separated. The aqueous phase was extracted with DCM (3 ×) and the combined organic phases were dried over $MgSO_4$, filtered, and concentrated under reduced pressure. Biotage Isolera^{TM} flash purification on silica gel (Sfär High Capacity 10 g silica cartridge), eluting with EtOAc/hexane (3 – 30%, v/v) gave alcohol **48** (27.3 mg, 98%) as a colourless oil.

R*f* = 0.26 (15:85 EtOAc/hexane, UV, cerium molybdate)

NMR Spectroscopy (*see spectra*):

> **^{1}H NMR** (400 MHz, $CDCl_3$): δ_H 7.53 – 7.49 (m, 2H), 7.47 – 7.39 (m, 3H), 7.33 – 7.28 (m, 2H), 7.22 – 7.17 (m, 3H), 4.74 (s, 1H), 2.96 – 2.82 (m, 2H), 2.65 – 2.58 (m, 1H), 2.36 (br s, 1H), 2.26 (ddd, *J* = 11.5, 9.1, 1.2 Hz, 1H), 2.16 (dd, *J* = 12.3, 8.9 Hz, 1H) ppm;
>
> **^{13}C NMR** (101 MHz, $CDCl_3$): δ_C 144.7, 135.5, 128.9, 128.8, 128.7, 128.5, 126.7, 126.3, 72.8, 72.0, 41.7, 40.1, 29.7 ppm.

IR (film): ν_{max} 3424, 3028, 2978, 2937, 2102 (N=N=N), 1494, 1454, 1248, 1097 cm^{-1}.

HRMS (APCI$^+$): m/z calc'd for $C_{17}H_{17}NO$ $[M+H–N_2]^+$, 252.1383; found, 252.1379.

cis-3-phenyl-1-(phenyl(1_H_-pyrazol-1-yl)methyl)cyclobutan-1-ol (49)[12]

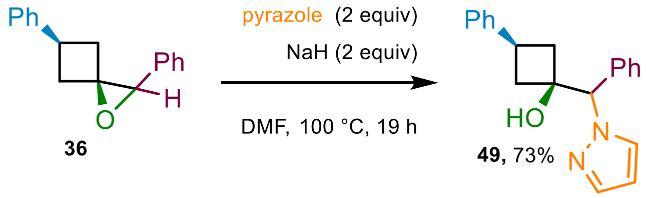

A flame dried microwave vial was charged with pyrazole (13.6 mg, 0.200 mmol, 2.00 equiv) and DMF (1.00 mL), before NaH (60% dispersion in mineral oil, 8.0 mg, 0.20 mmol, 2.0 equiv) was added at room temperature.[A] After 10 min the gas formation had ceased and epoxide **36** (23.6 mg, 0.100 mmol, 1.00 equiv) was added. The vial was sealed and heated to 100 °C for 19 h. After cooling to room temperature, the resulting orange suspension was quenched by addition of water, then EtOAc was added, and the phases were separated. The aqueous phase was extracted with EtOAc (3 ×), and the combined organic phases were dried over $MgSO_4$, filtered, and concentrated under reduced pressure. Biotage Isolera™ flash purification on silica gel (Sfär High Capacity 10 g silica cartridge), eluting with EtOAc/hexane (3 – 30%, v/v) gave alcohol **49** (22.1 mg, 73%) as white amorphous solid.

Notes: (**A**) DMF in combination with NaH forms a potential safety hazard,[13] which was remedied by performing the reaction in a microwave vial.

R*f* = 0.27 (85:15 EtOAc/hexane, UV, cerium molybdate)

NMR Spectroscopy (*see spectra*):

^{1}**H NMR** (400 MHz, $CDCl_3$): δ_H 7.59 – 7.59 (m, 1H), 7.54 (dd, *J* = 2.3, 0.7 Hz, 1H), 7.45 – 7.42 (m, 2H), 7.37 – 7.25 (m, 7H), 7.21 – 7.17 (m, 1H), 6.31 (t, *J* = 2.1 Hz, 1H), 5.65 (s, 1H), 5.43 (s, 1H), 3.22 (apparent p, *J* = 9.1 Hz, 1H), 2.71 (dddd, *J* = 12.3, 8.4, 4.9, 0.8 Hz, 1H), 2.48 (dddd, *J* = 12.1, 8.5, 4.9, 0.8 Hz, 1H), 2.36 – 2.26 (m, 2H) ppm;

^{13}**C NMR** (101 MHz, $CDCl_3$): δ_C 144.8, 139.1, 137.0, 131.0, 128.6, 128.5, 128.4, 128.3,126.8, 126.2, 105.6, 73.3, 70.2, 43.0, 40.2, 30.8 ppm.

IR (film): ν_{max} 3358, 3027, 2977, 2935, 1495, 1455, 1497, 1288, 1245, 1092 cm^{-1}.

HRMS (ESI^+): m/z calc'd for $C_{20}H_{21}N_2O$ $[M+H]^+$, 305.1648; found, 305.1644.

cis-3-phenyl-1-(phenyl(piperidin-1-yl)methyl)cyclobutan-1-ol (50)[14]

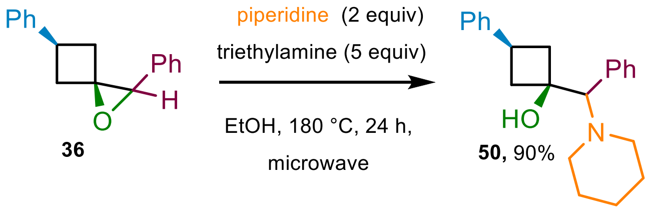

A flame dried microwave vial was charged with epoxide **36** (23.6 mg, 0.100 mmol, 1.00 equiv) and ethanol (1.00 mL), before piperidine (20.0 µL, 17.2 mg, 0.202 mmol, 2.02 equiv) and triethylamine (70.0 µL, 50.8 mg, 0.502 mmol, 5.02 equiv) were added. The vial was sealed and heated to 180 °C for 24 h under microwave irradiation. After cooling to room temperature, the resulting orange solution was concentrated under reduced pressure. Biotage Isolera™ flash purification on silica gel (Sfär High Capacity 10 g silica cartridge), eluting with EtOAc/hexane (12 – 80%, v/v) gave alcohol **50** (29.0 mg, 90%) as a pale-yellow oil.

R*f* = 0.32 (50:50 EtOAc/hexane, UV, cerium molybdate)

NMR Spectroscopy (*see spectra*):

> ^{1}**H NMR** (400 MHz, $CDCl_3$): δ_H 7.38 – 7.24 (m, 7H), 7.21 – 7.19 (m, 2H), 7.17 – 7.12 (m, 1H), 3.47 (s, 1H), 2.89 – 2.76 (m, 2H), 2.59 – 2.53 (m, 2H), 2.46 – 2.39 (m, 3H), 2.25 – 2.15 (m, 2H), 1.60 – 1.54 (m, 4H), 1.47 – 1.41 (m, 2H) ppm; The proton attached to oxygen was not visible.
>
> ^{13}**C NMR** (101 MHz, $CDCl_3$): δ_C 145.5, 139.4, 129.3, 128.4, 128.3, 127.6, 126.8, 125.9, 76.8, 71.5, 53.4, 47.2, 42.5, 30.2, 26.5, 24.5 ppm.

IR (film): ν_{max} 3401, 3026, 2931, 2853, 2804, 1494, 1453, 1384, 1307, 1246, 1082 cm^{-1}.

HRMS (ESI$^+$): m/z calc'd for $C_{22}H_{28}NO$ [M+H]$^+$, 322.2165; found, 322.2157.

cis-1-(phenoxy(phenyl)methyl)-3-phenylcyclobutan-1-ol (51)[12]

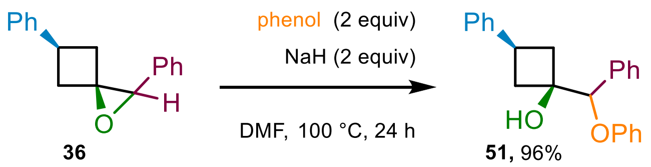

A flame dried microwave vial was charged with phenol (18.8 mg, 0.200 mmol, 2.00 equiv) and DMF (1.00 mL), before NaH (60% dispersion in mineral oil, 8.0 mg, 0.20 mmol, 2.0 equiv) was added at room temperature.[A] After 10 min the gas formation had ceased and epoxide **36** (23.6 mg, 0.100 mmol, 1.00 equiv) was added. The vial was sealed and heated to 100 °C for 24 h. After cooling to room temperature, the resulting orange suspension was quenched by addition of water, then EtOAc was added, and the phases were separated. The aqueous phase was extracted with EtOAc (3 ×), and the combined organic phases were dried over $MgSO_4$, filtered, and concentrated under reduced pressure. Biotage Isolera™ flash purification on silica gel (Sfär High Capacity 10 g silica cartridge), eluting with EtOAc/hexane (3 – 30%, v/v) gave alcohol **51** (31.6 mg, 96%) as a colourless oil.

Notes: (**A**) DMF in combination with NaH forms a potential safety hazard,[13] which was remedied by performing the reaction in a microwave vial.

R*f* = 0.24 (15:85 EtOAc/hexane, UV, cerium molybdate)

NMR Spectroscopy (*see spectra*):

> **^{1}H NMR** (400 MHz, $CDCl_3$): δ_H 7.54 – 7.51 (m, 2H), 7.42 – 7.28 (m, 5H), 7.25 – 7.17 (m, 5H), 6.94 – 6.90 (m, 3H), 5.22 (s, 1H), 2.96 – 2.81 (m, 3H), 2.64 (s, 1H), 2.37 – 2.31 (m, 1H), 2.30 – 2.22 (m, 1H) ppm;
>
> **^{13}C NMR** (101 MHz, $CDCl_3$): δ_C 158.1, 145.2, 136.9, 129.6, 128.6, 128.4, 128.4, 127.7, 126.7, 126.1, 121.3, 116.1, 83.9, 72.9, 40.6, 39.9, 30.0 ppm.

IR (film): ν_{max} 3429, 3027, 2937, 2979, 1598, 1492, 1234 (C-O), 1029 cm^{-1}.

HRMS (Nanospray^{+}): m/z calc'd for $C_{23}H_{22}O_2Na$ $[M+Na]^{+}$, 353.1517; found, 353.1517.

cis-3-phenyl-1-(phenyl(phenylthio)methyl)cyclobutan-1-ol (52)[15]

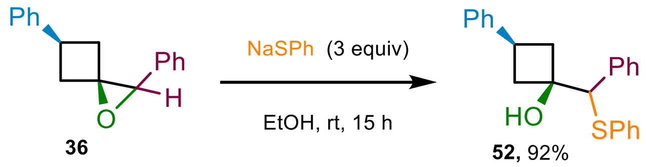

A flame dried Schlenk flask was charged with sodium thiophenolate (39.6 mg, 0.300 mmol, 3.00 equiv) and ethanol (1.00 mL) under nitrogen at room temperature, before epoxide **36** (23.6 mg, 0.100 mmol, 1.00 equiv) was added. After complete addition, the resulting colourless suspension was stirred at room temperature for 15 h. A saturated aqueous solution of $NaHCO_3$ was added to quench the reaction, then Et_2O was added, and the phases were separated. The aqueous phase was extracted with Et_2O (3 ×) and the combined organic phases were dried over $MgSO_4$, filtered, and concentrated under reduced pressure. Biotage Isolera™ flash purification on silica gel (Sfär High Capacity 10 g silica cartridge), eluting with EtOAc/hexane (3 – 30%, v/v) gave alcohol **52** (31.9 mg, 92%) as a yellow crystalline solid.

m.p.: = 92 – 93 °C (DCM)

R*f* = 0.27 (15:85 EtOAc/hexane, UV, cerium molybdate)

NMR Spectroscopy (*see spectra*):

^{1}**H NMR** (400 MHz, $CDCl_3$): δ_H 7.56 – 7.53 (m, 2H), 7.37 – 7.32 (m, 4H), 7.31 – 7.27 (m, 3H), 7.24 – 7.16 (m, 6H), 4.46 (s, 1H), 2.97 (apparent p, *J* = 8.8 Hz, 1H), 2.92 – 2.86 (m, 1H), 2.70 (s, 1H), 2.65 (dddd, *J* = 12.2, 8.5, 5.0, 0.7 Hz, 1H), 2.34 (dd, *J* = 11.8, 9.0 Hz, 1H), 2.17 (dd, *J* = 12.2, 9.2 Hz, 1H) ppm;

^{13}**C NMR** (101 MHz, $CDCl_3$): δ_C 144.9, 139.1, 135.6, 131.8, 129.4, 129.0, 128.5, 128.5, 127.8, 127.2, 126.7, 126.2, 73.6, 63.5, 43.4, 41.8, 30.0. ppm.

IR (film): ν_{max} 3451, 3058, 3025, 2976, 2934, 1583, 1494, 1451, 1235, 1085 cm^{-1}.

HRMS (ESI^+): m/z calc'd for $C_{23}H_{22}NaOS$ $[M+Na]^+$, 369.128357; found, 369.129254.

2.6. Unsuccessful Substrates

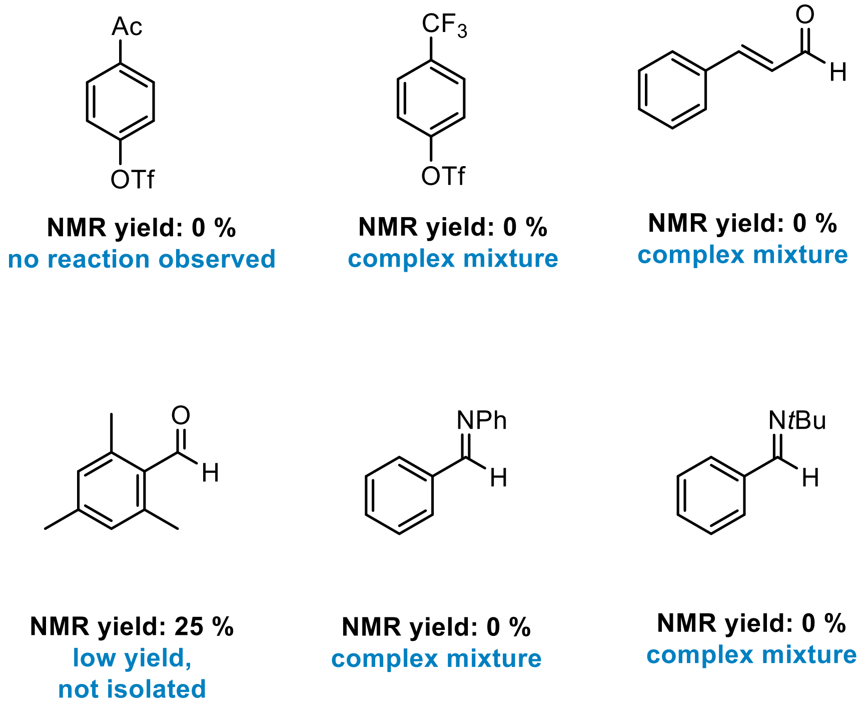

Figure 2. Unsuccessful Substrates

Reactions performed according to general procedures on a 0.3 mmol scale. NMR yield was determined by ^{1}H NMR analysis using dibromomethane as an internal standard.

3. X-RAY CRYSTALLOGRAPHIC ANALYSIS

3.1. 26 (CCDC number: 2115071) and 43 (CCDC number: 2115072)

X-ray diffraction experiments on **26** were carried out at 100(2) K on a Bruker APEX II diffractometer using Mo-K_α radiation (λ = 0.71073 Å) and a CCD area detector, while **43** was carried out at 100(2) K on a Bruker D8 Venture diffractometer using Mo-K_α (λ = 0.71073 Å) and a CPAD detector. Intensities were integrated in SAINT[16] and absorption corrections based on equivalent reflections were applied using SADABS.[17] Structures were solved using ShelXT[18] all of the structures were refined by full matrix least squares against F^2 in ShelXL[19] using Olex2.[20] All of the non-hydrogen atoms were refined anisotropically. While all the hydrogen atoms were located geometrically and refined using a riding model. **26** is in a chiral space group but the absolute structure was not determined. The Flack parameter is ambiguous and has been removed from the CIF. In the case of **43** the molecule displayed disorder in the methyl group, it was modelled in two positions with a refined occupancy ratio of 0.52:0.48(4). SADI and SIMU were used to maintain sensible geometries and thermal parameters. The crystal structure and refinement data are given in Table 2. Crystallographic data for compounds **26** and **43** has been deposited with the Cambridge Crystallographic Data Centre as supplementary publication CCDC 2115071-2115072. Copies of the data can be obtained free of charge on application to CCDC, 12 Union Road, Cambridge CB2 1EZ, UK [fax(+44) 1223 336033, e-mail: deposit@ccdc.cam.ac.uk].

**Compound**	**26**	**43**
CCDC number	2115071	2115072
Empirical formula	$C_{17}H_{16}NOCl$	$C_{24}H_{22}ClNO_2S$
Formula weight	285.76	423.93
Temperature/K	99.89	100.0
Crystal system	monoclinic	monoclinic
Space group	$P2_1$	$P2_1/c$
a/Å	7.8896(2)	21.9014(10)
b/Å	5.67280(10)	12.1496(6)
c/Å	15.8881(5)	7.7355(4)
α/°	90	90
β/°	100.080(2)	94.301(2)
γ/°	90	90
Volume/Å^3	700.11(3)	2052.57(17)
Z	2	4
ρ_{calc}g/cm^3	1.356	1.372
μ/mm^{-1}	0.267	0.309
F(000)	300.0	888.0
Crystal size/mm^3	0.4 × 0.19 × 0.12	0.36 × 0.308 × 0.08
Radiation	MoKα (λ = 0.71073)	MoKα (λ = 0.71073)
2Θ range for data collection/°	5.208 to 54.196	3.836 to 55.802
Index ranges	-10 ≤ h ≤ 10<br>-7 ≤ k ≤ 7<br>-17 ≤ l ≤ 20	-28 ≤ h ≤ 28<br>-15 ≤ k ≤ 15<br>-10 ≤ l ≤ 10
Reflections collected	9113	51797
Independent reflections	3087 [R_{int} = 0.0194, R_{sigma} = 0.0223]	4885 [R_{int} = 0.0463, R_{sigma} = 0.0273]
Data/restraints/parameters	3087/1/182	4885/7/274
Goodness-of-fit on F^2	1.045	1.027
Final R indexes [I>=2σ (I)]	R_1 = 0.0279<br>wR_2 = 0.0688	R_1 = 0.0373<br>wR_2 = 0.0820
Final R indexes [all data]	R_1 = 0.0296<br>wR_2 = 0.0698	R_1 = 0.0472<br>wR_2 = 0.0858
Largest diff. peak/hole / e Å^{-3}	0.22/-0.18	0.57/-0.38

Table 2: Crystal data and structure refinement for 26 and 43

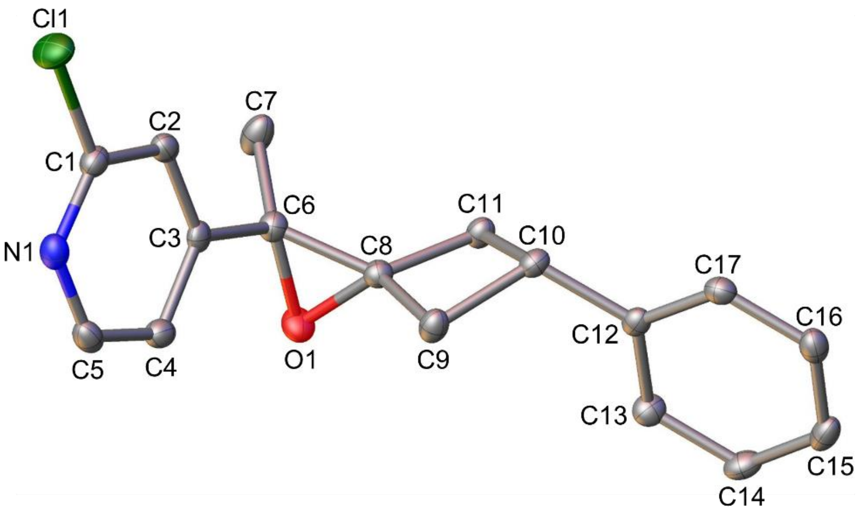

Figure 3. Crystal structure of 26 with the anisotropic displacement parameters depicted at the 50% probability level and hydrogens omitted for clarity.

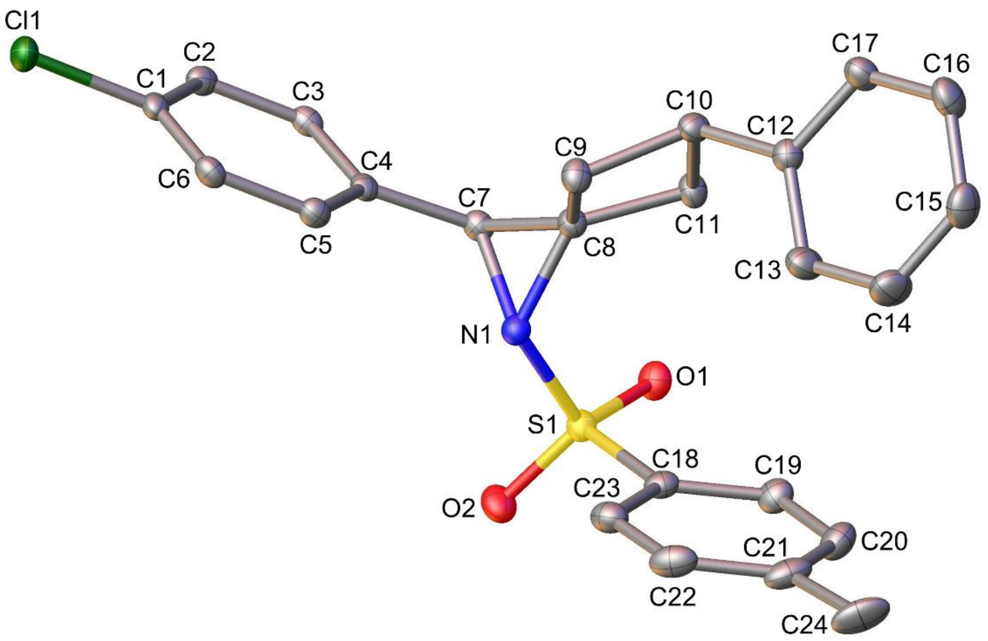

Figure 4. Crystal structure of 43 with the anisotropic displacement parameters depicted at the 50% probability level. Disorder and hydrogens omitted for clarity.

4. SPECTROSCOPIC DATA

crude ^{1}H NMR (400 MHz, $CDCl_3$) of **12** (*see procedure*)

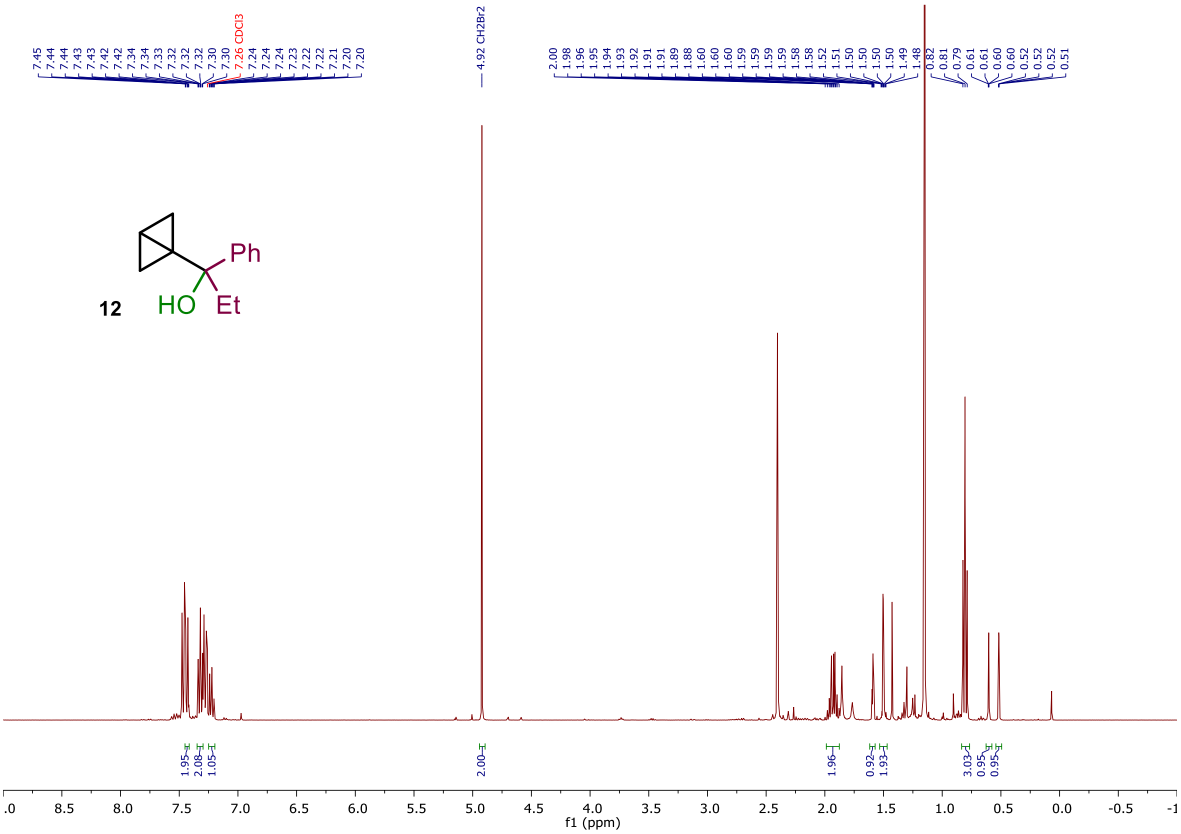

12

^{1}H NMR (400 MHz, $CDCl_3$) of **13** (*see procedure*)

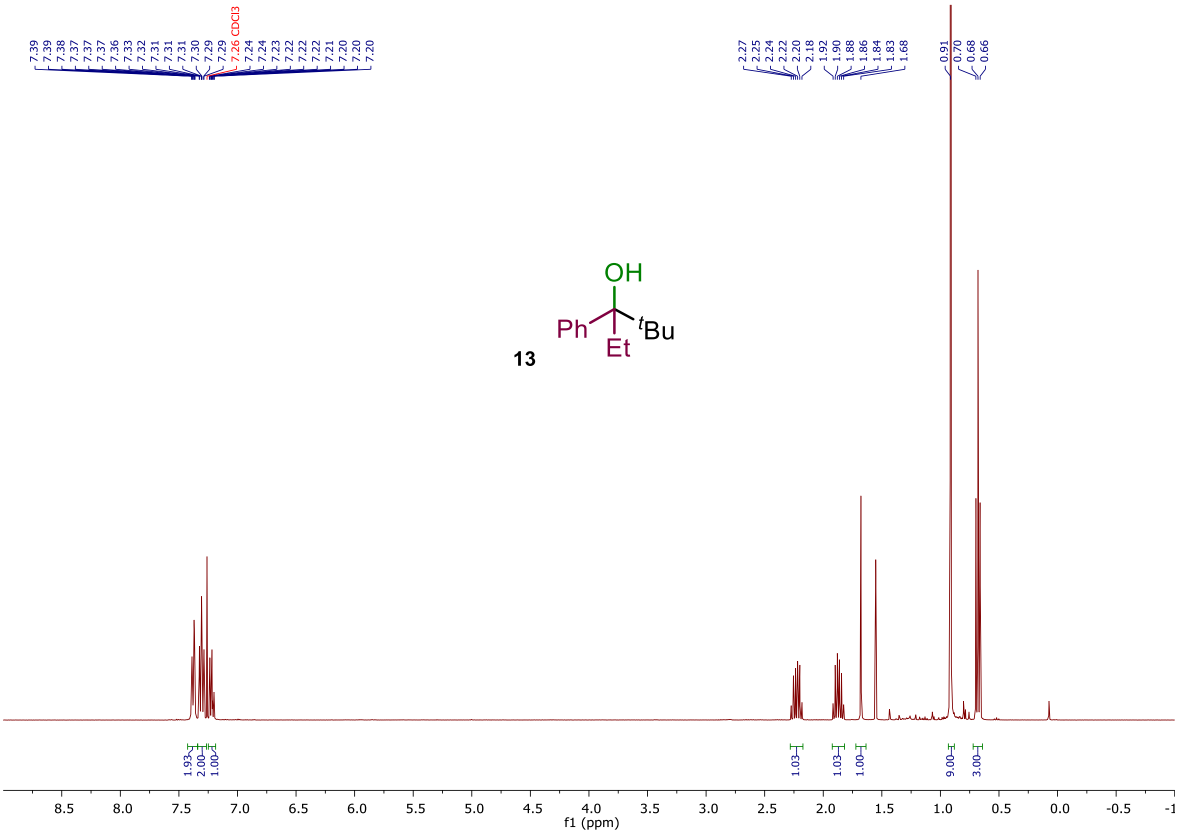

^{13}C NMR (101 MHz, $CDCl_3$) of **13**

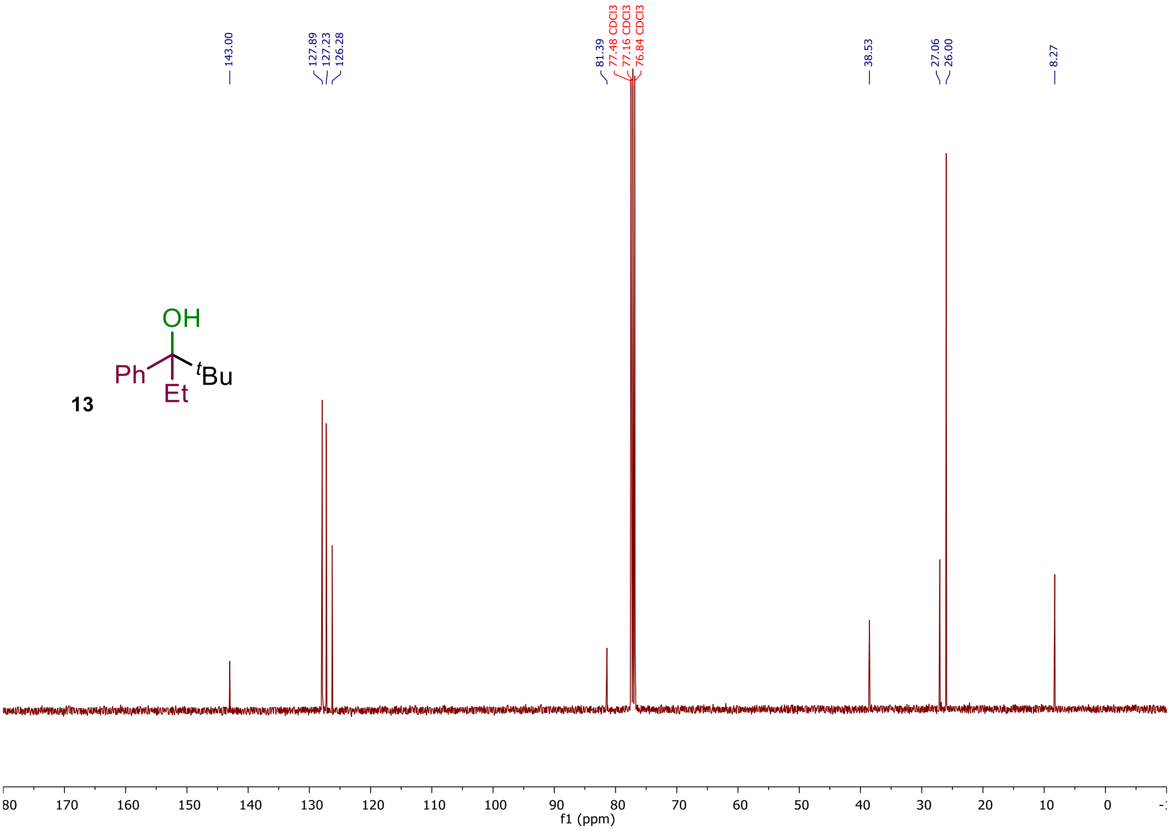

^1H NMR (400 MHz, $CDCl_3$) of **14** (*see procedure*)

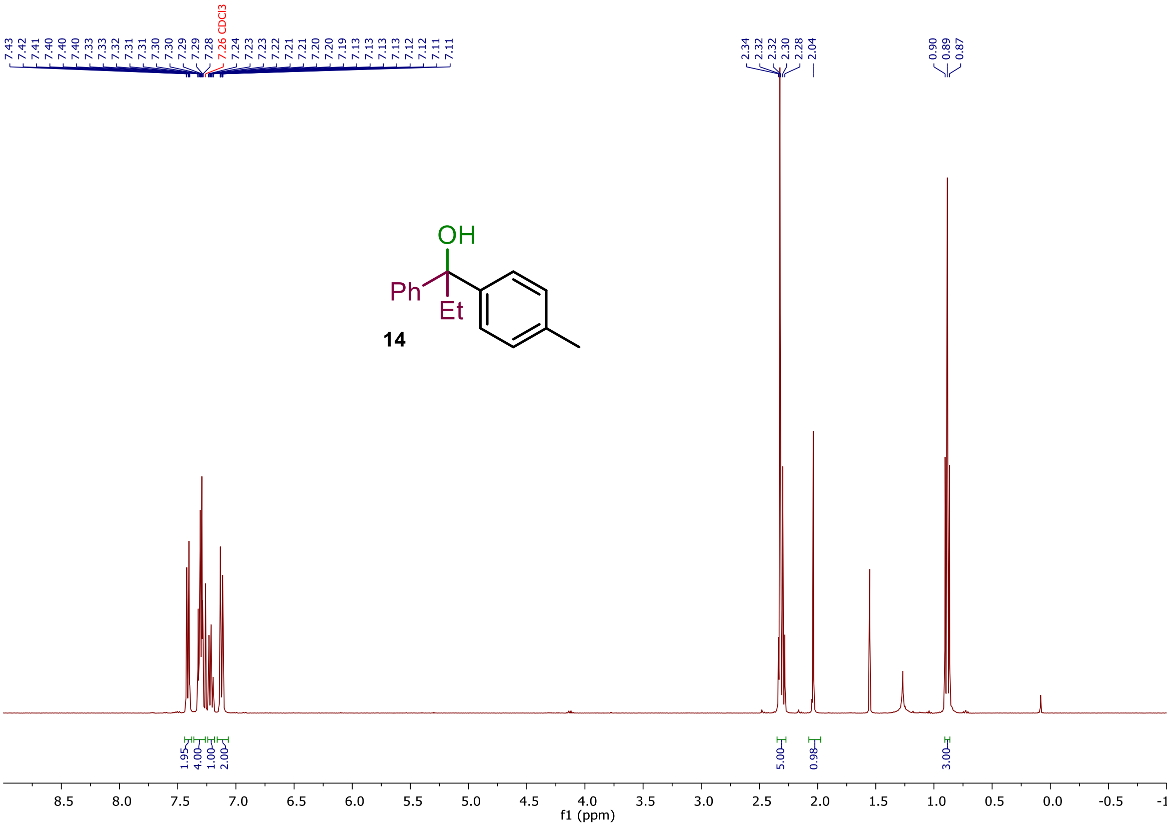

^{13}C NMR (101 MHz, $CDCl_3$) of **14**

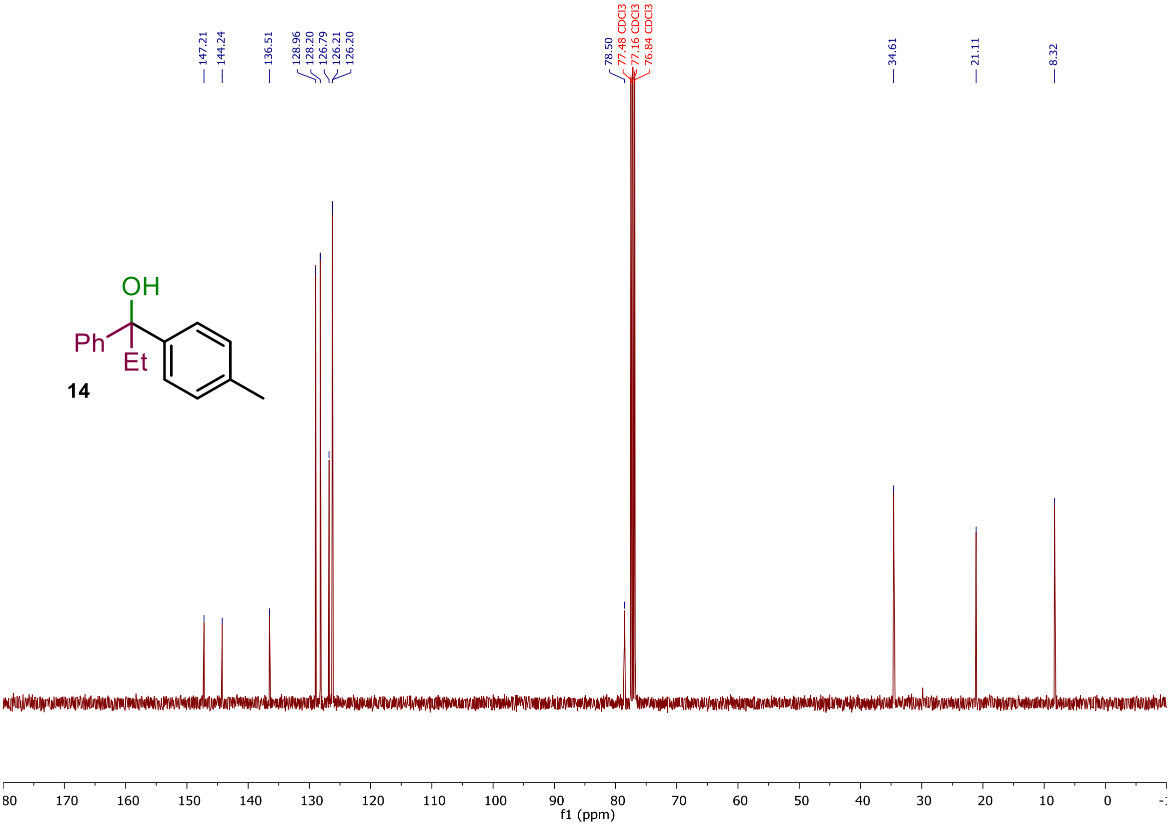

^{1}H NMR (400 MHz, $CDCl_3$) of **15** (*see procedure*)

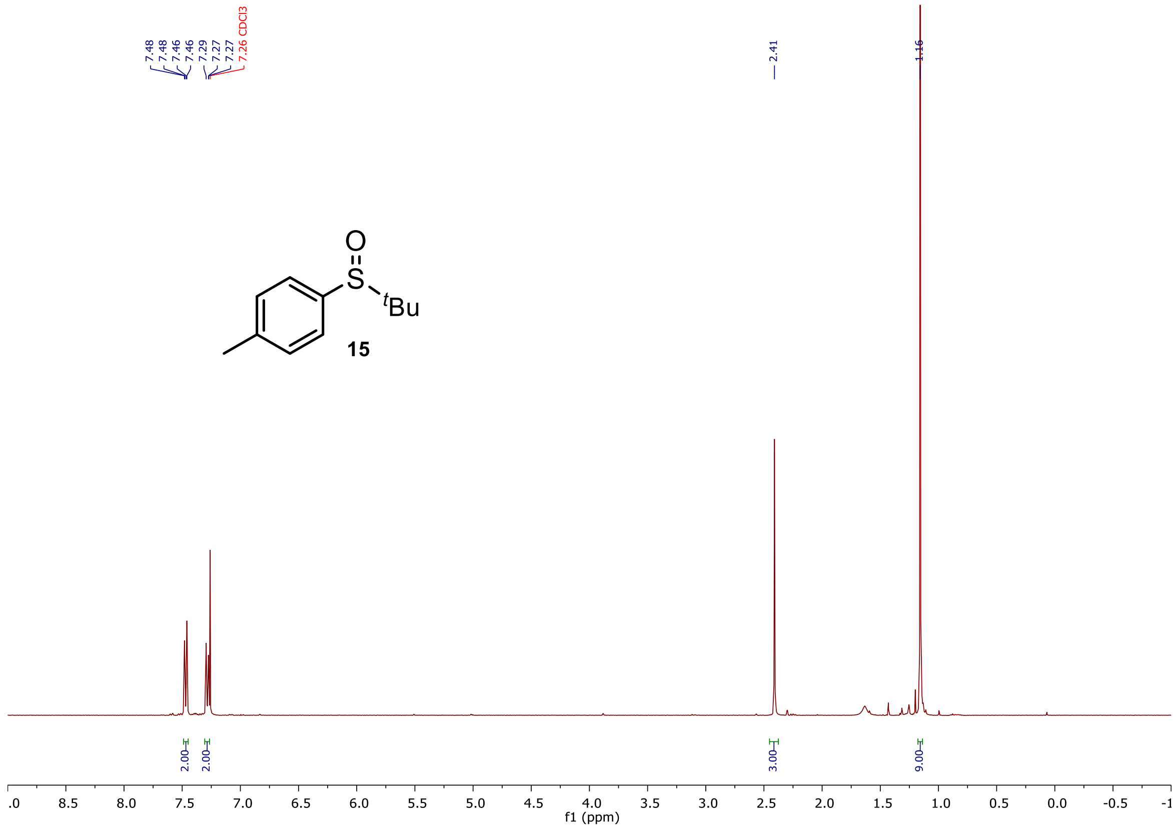

^{13}C NMR (101 MHz, $CDCl_3$) of **15**

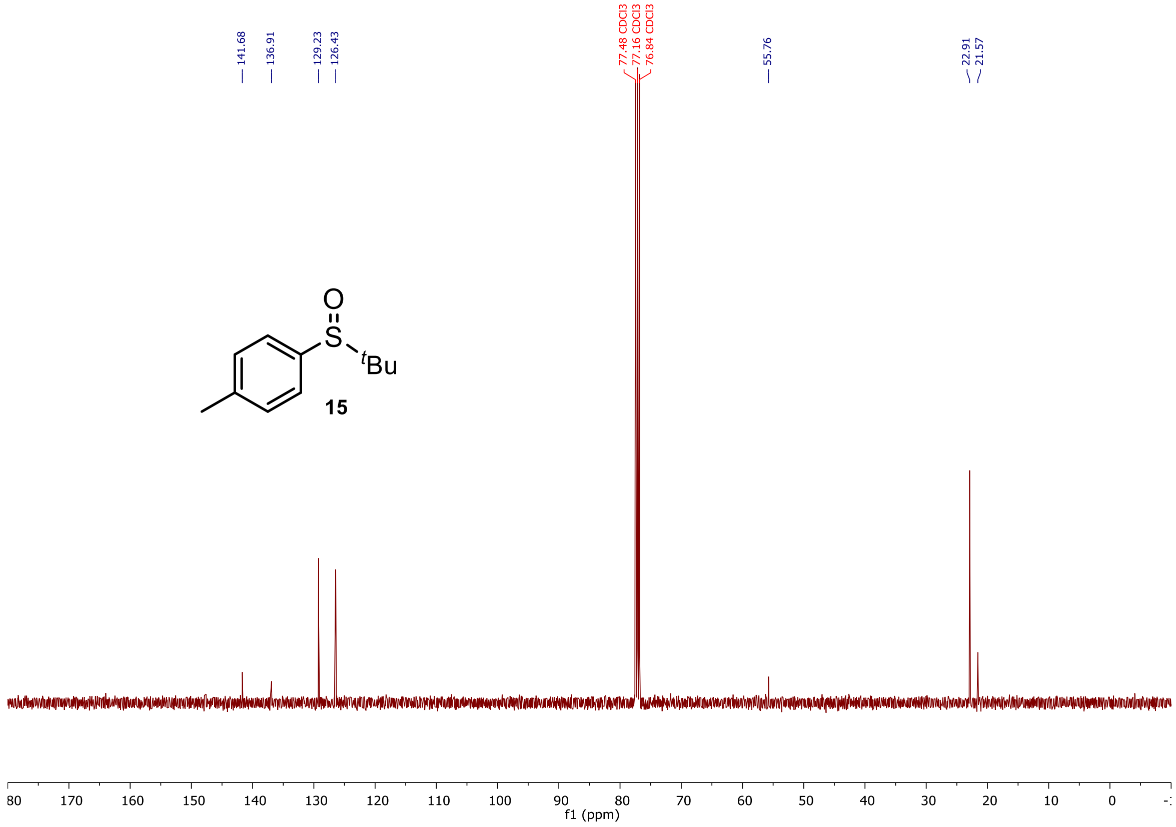

^{1}H NMR (400 MHz, CDCl3) of **18** (*see procedure*)

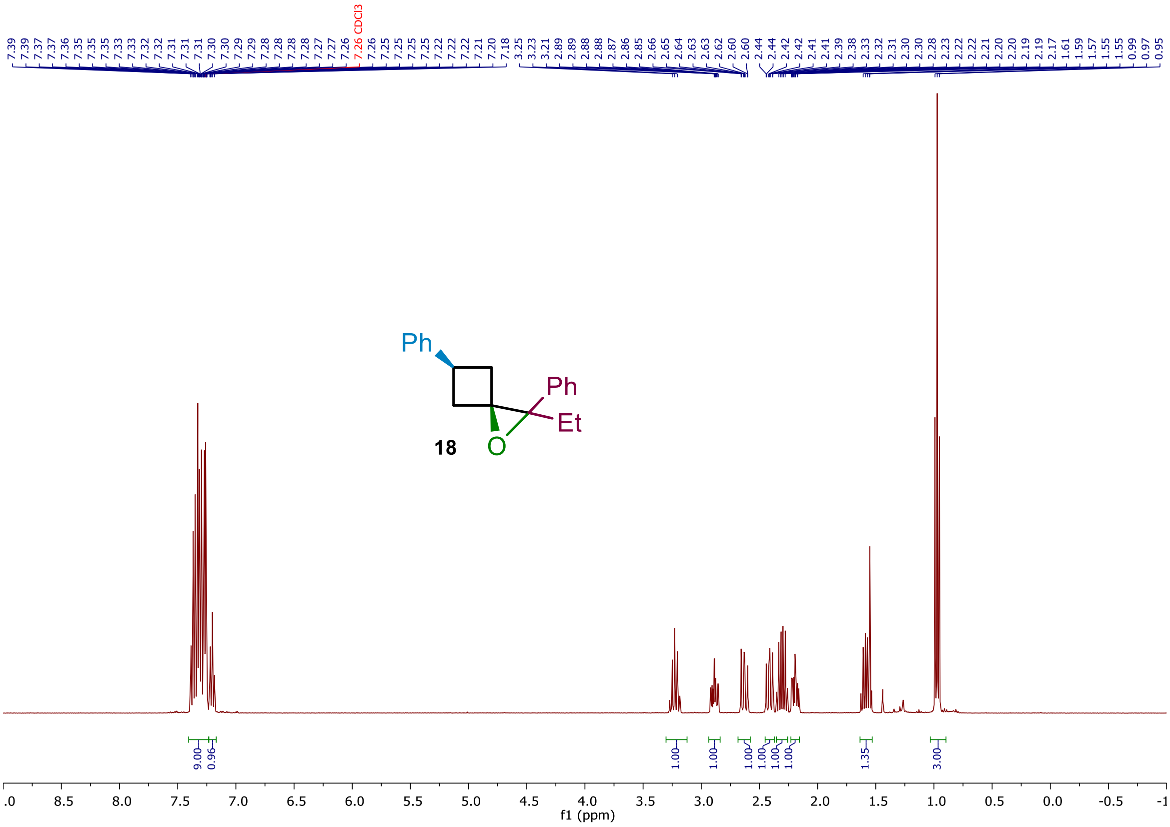

^{13}C NMR (101 MHz, CDCl3) of **18**

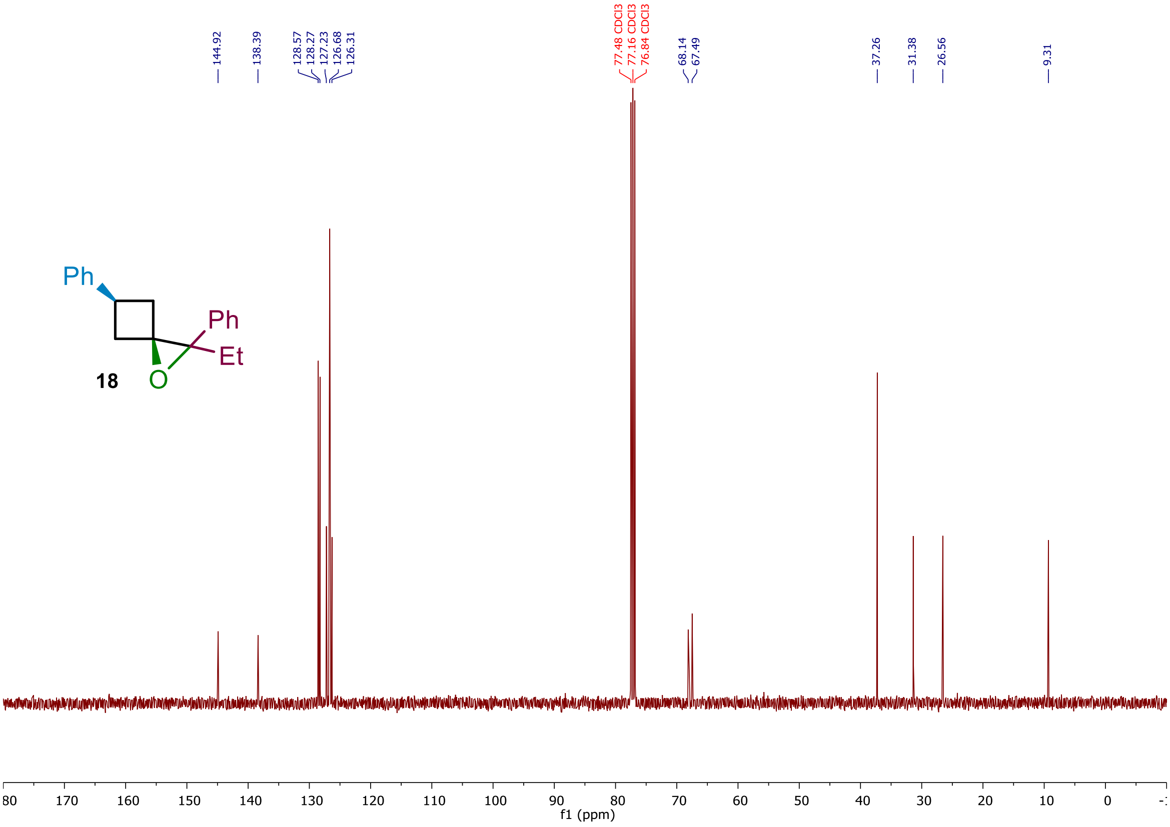

crude ^{1}H NMR (400 MHz, $CDCl_3$) of **22** (*see procedure*)

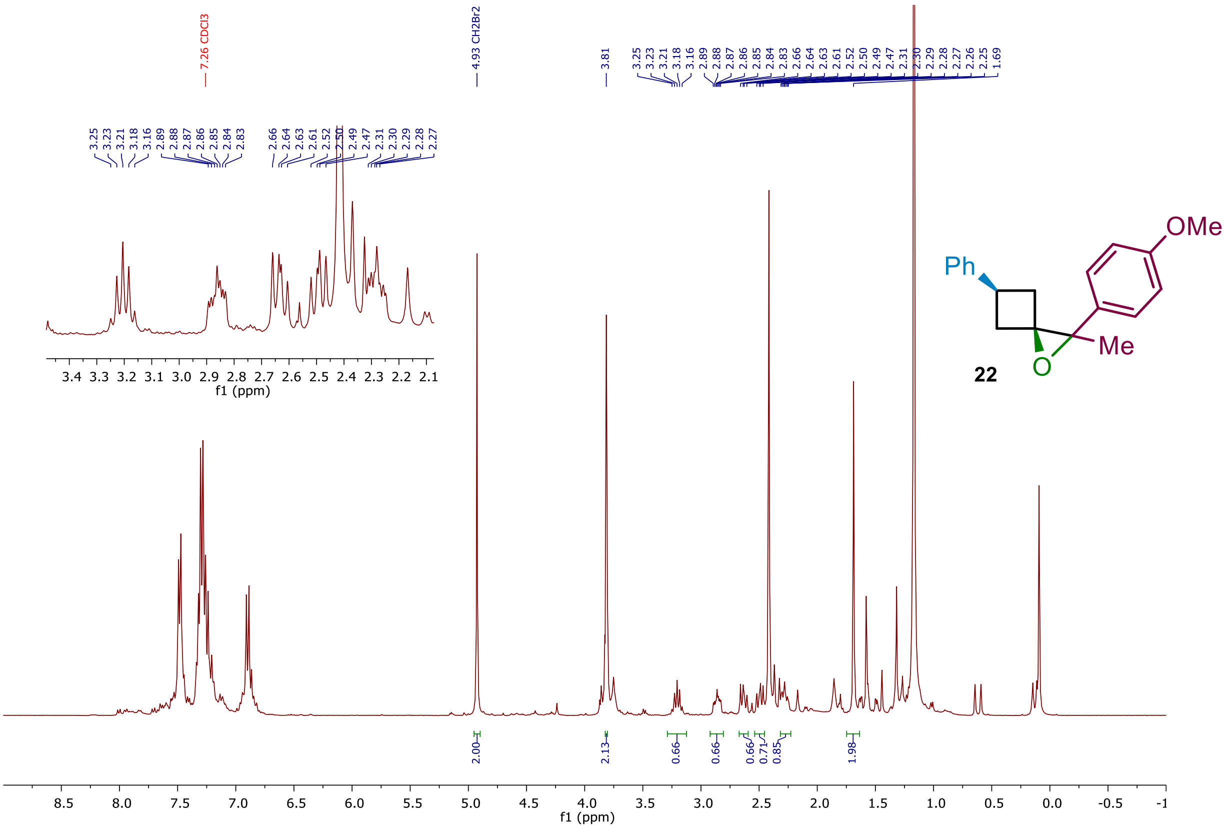

^{1}H NMR (500 MHz, $CDCl_3$) of **23** (*see procedure*)

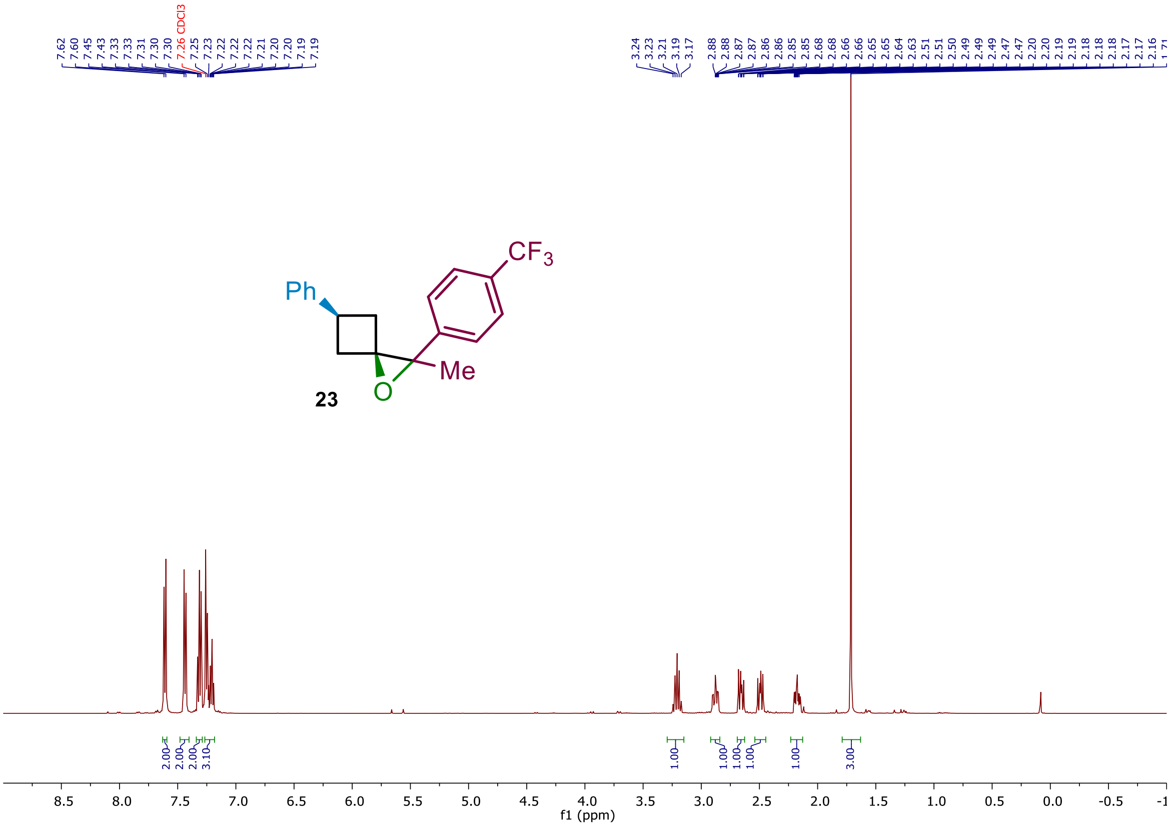

^{13}C NMR (126 MHz, $CDCl_3$) of **23**

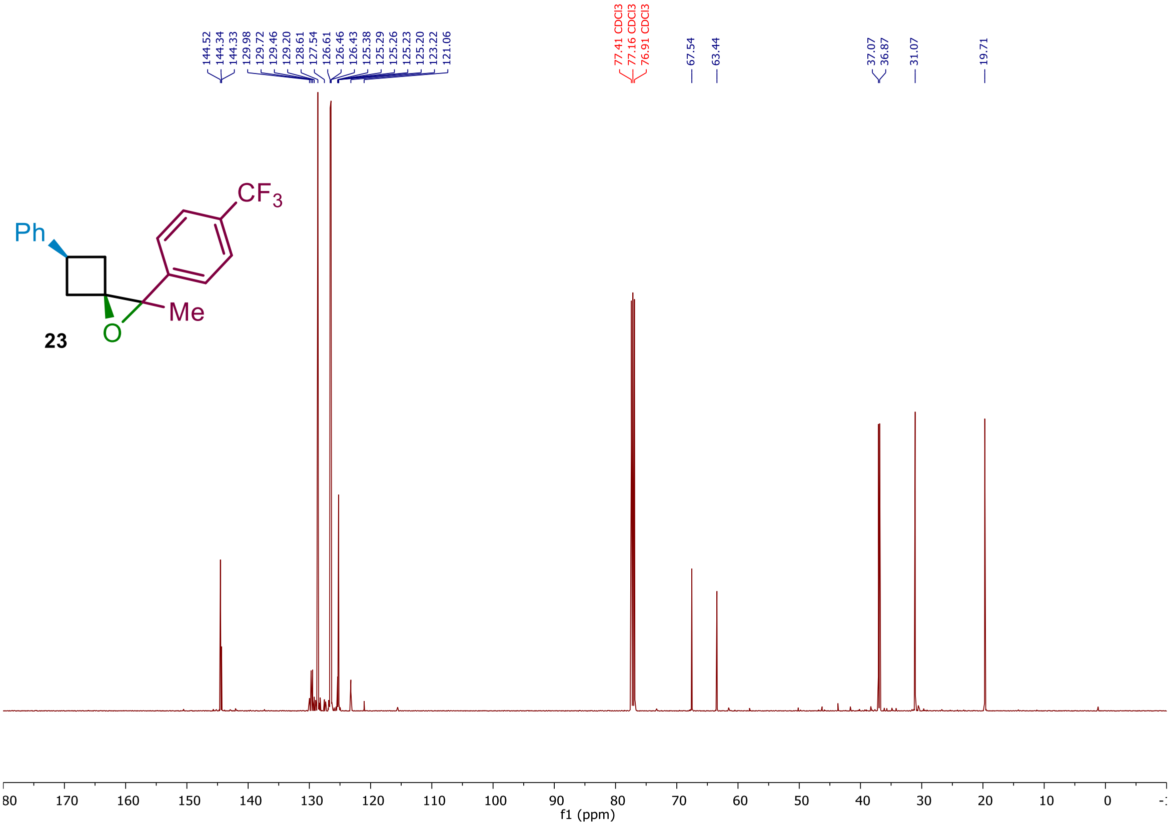

^{19}F NMR (377 MHz, $CDCl_3$) of **23**

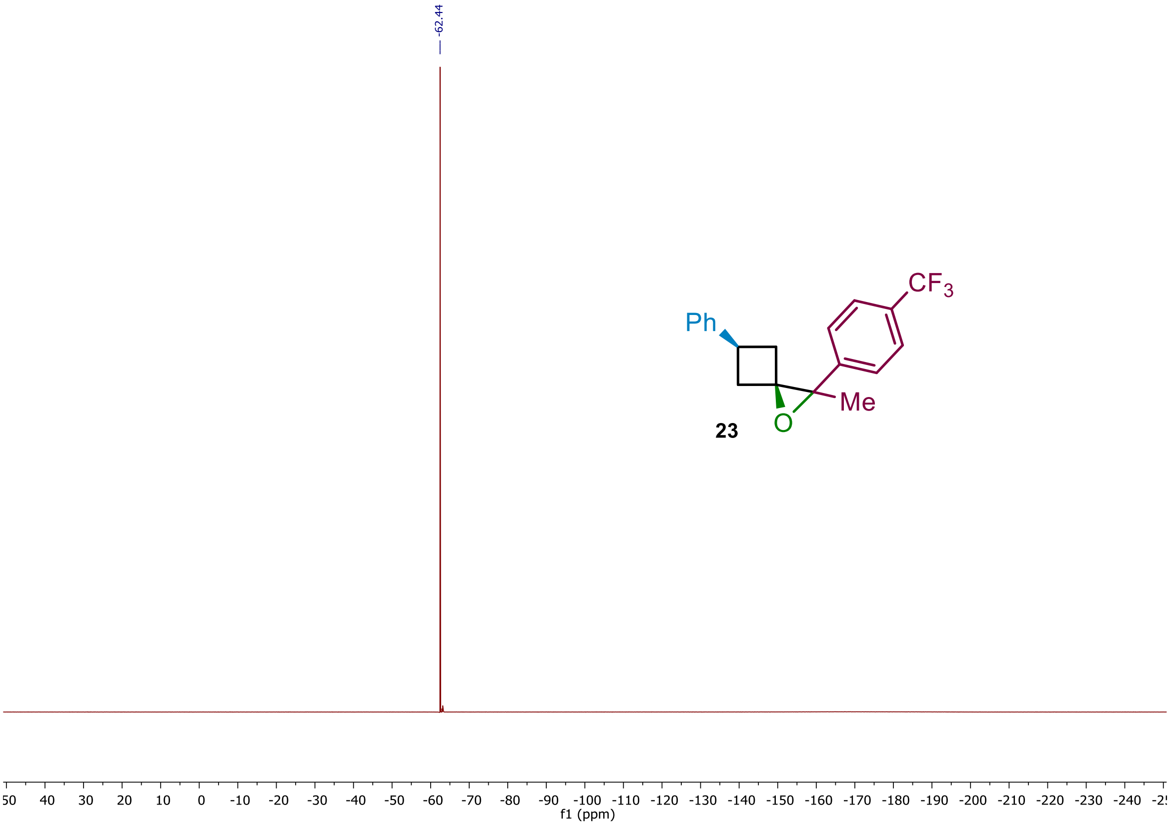

^{1}H NMR (500 MHz, $CDCl_3$) of **24** (*see procedure*)

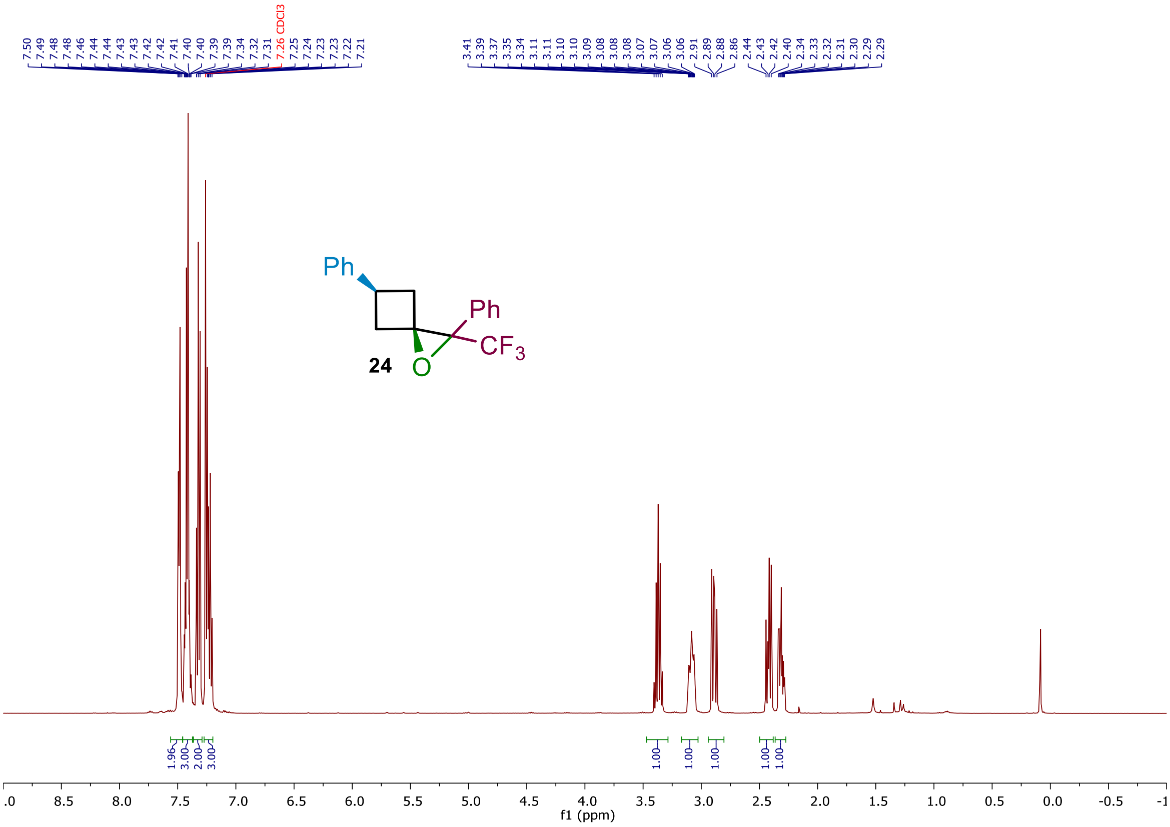

^{13}C NMR (126 MHz, $CDCl_3$) of **24**

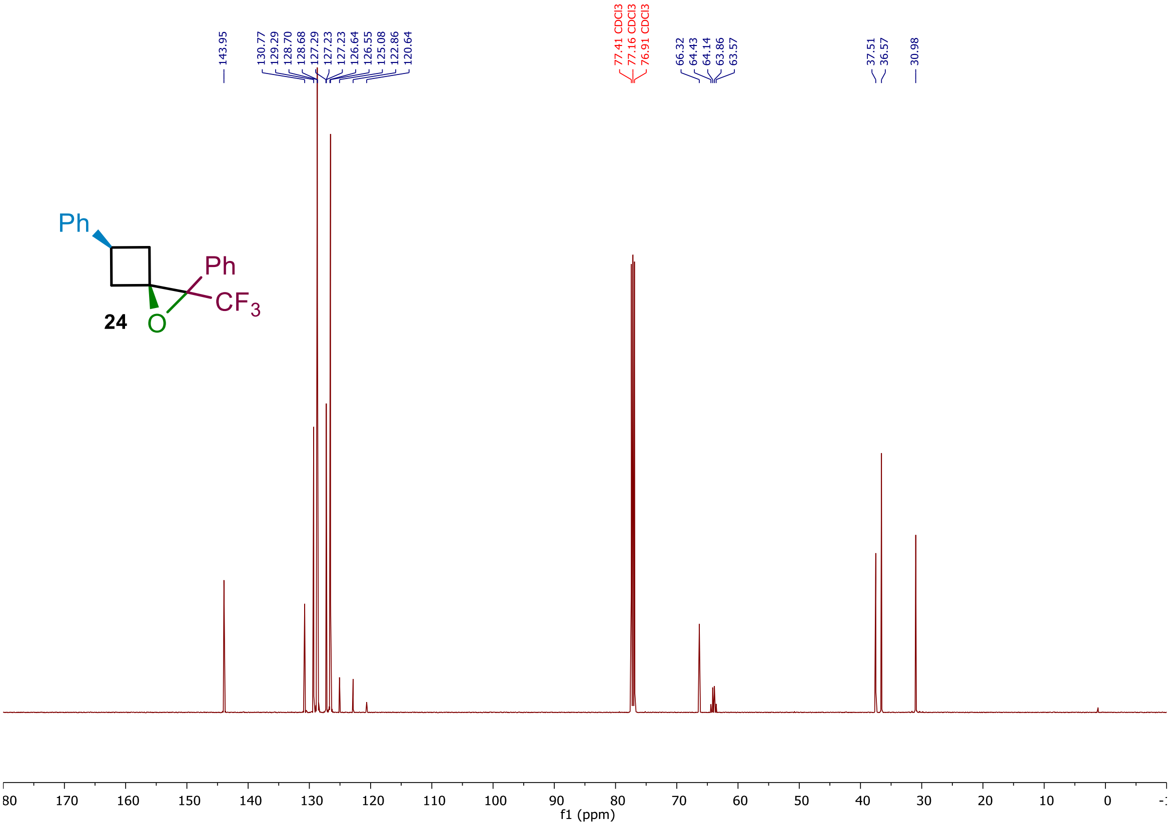

^{19}F NMR (377 MHz, $CDCl_3$) of **24**

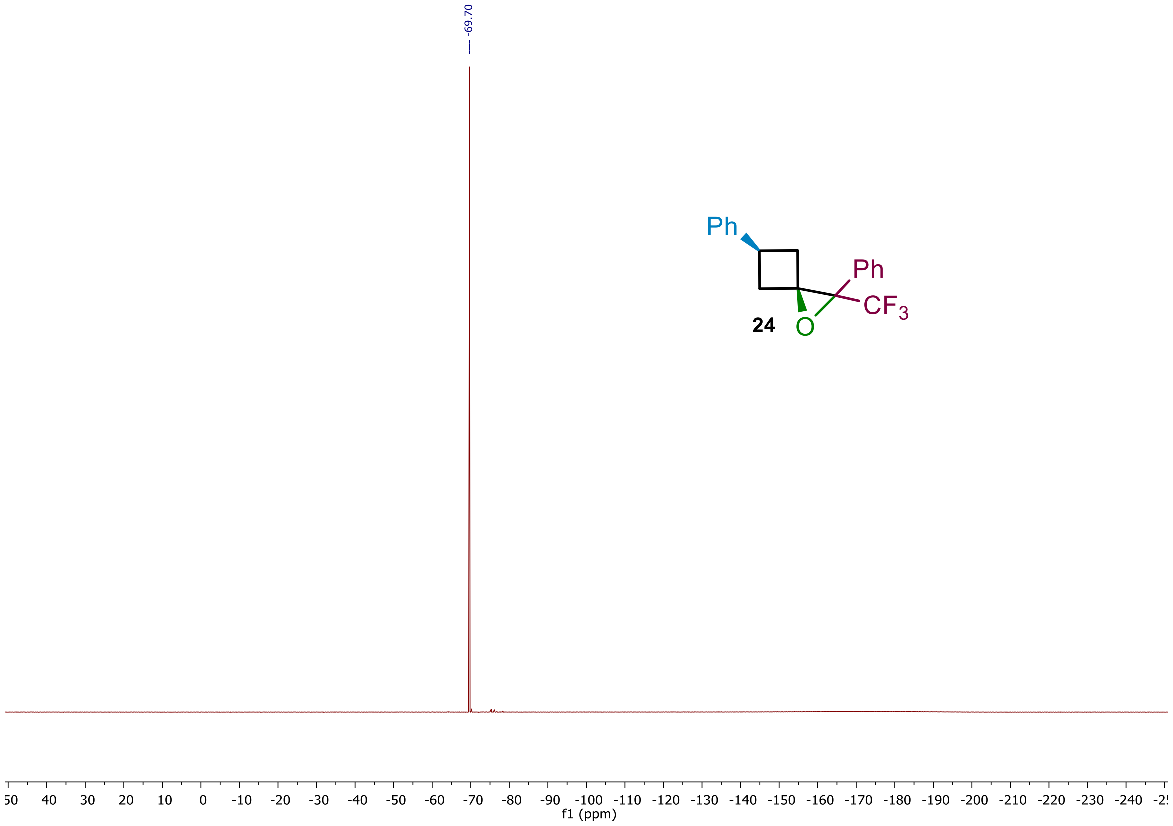

^{1}H NMR (400 MHz, $CDCl_3$) of **25** (*see procedure*)

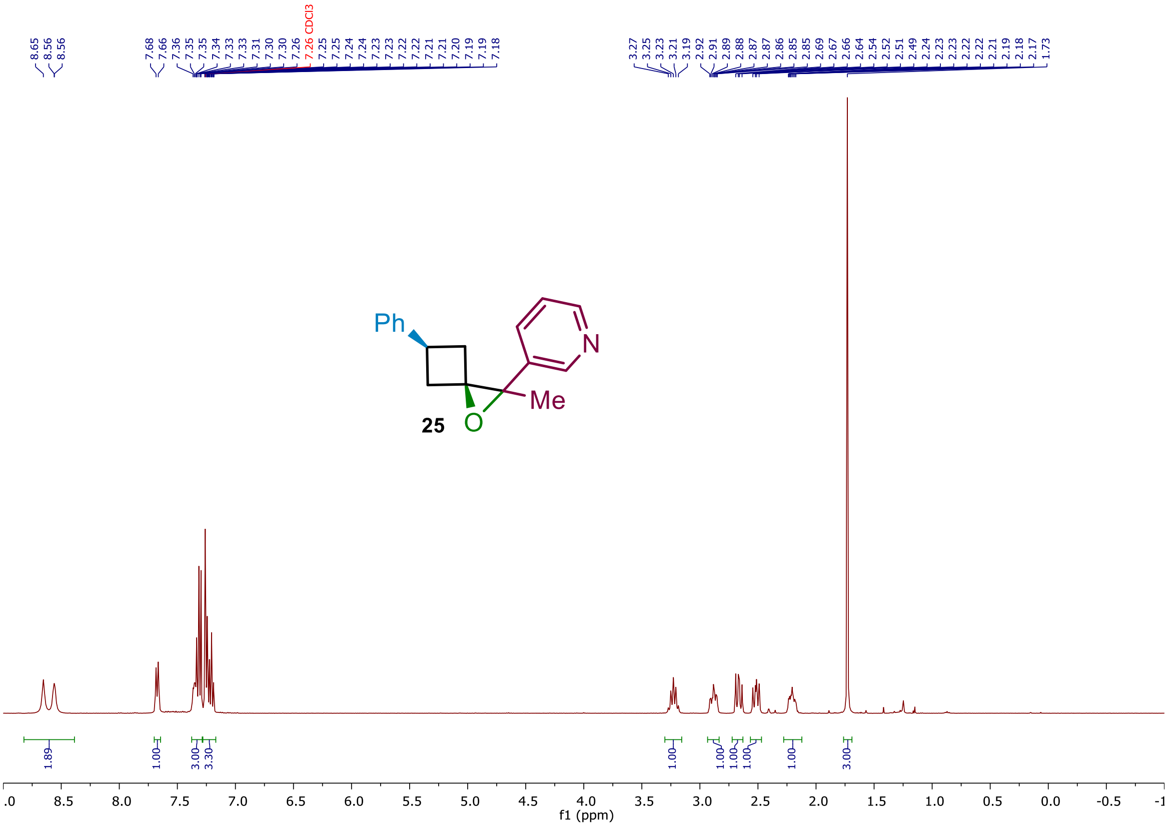

^{13}C NMR (101 MHz, $CDCl_3$) of **25**

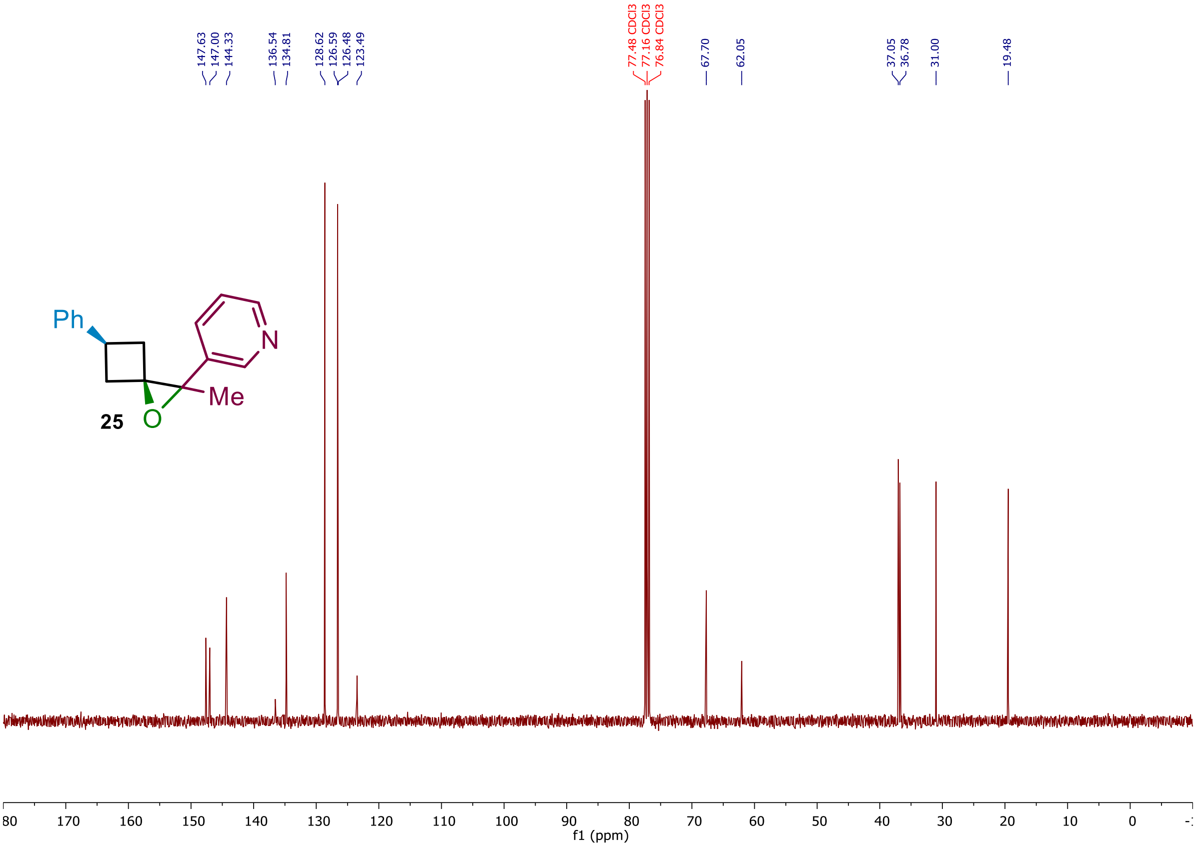

^{1}H NMR (400 MHz, $CDCl_3$) of **26** (*see procedure*)

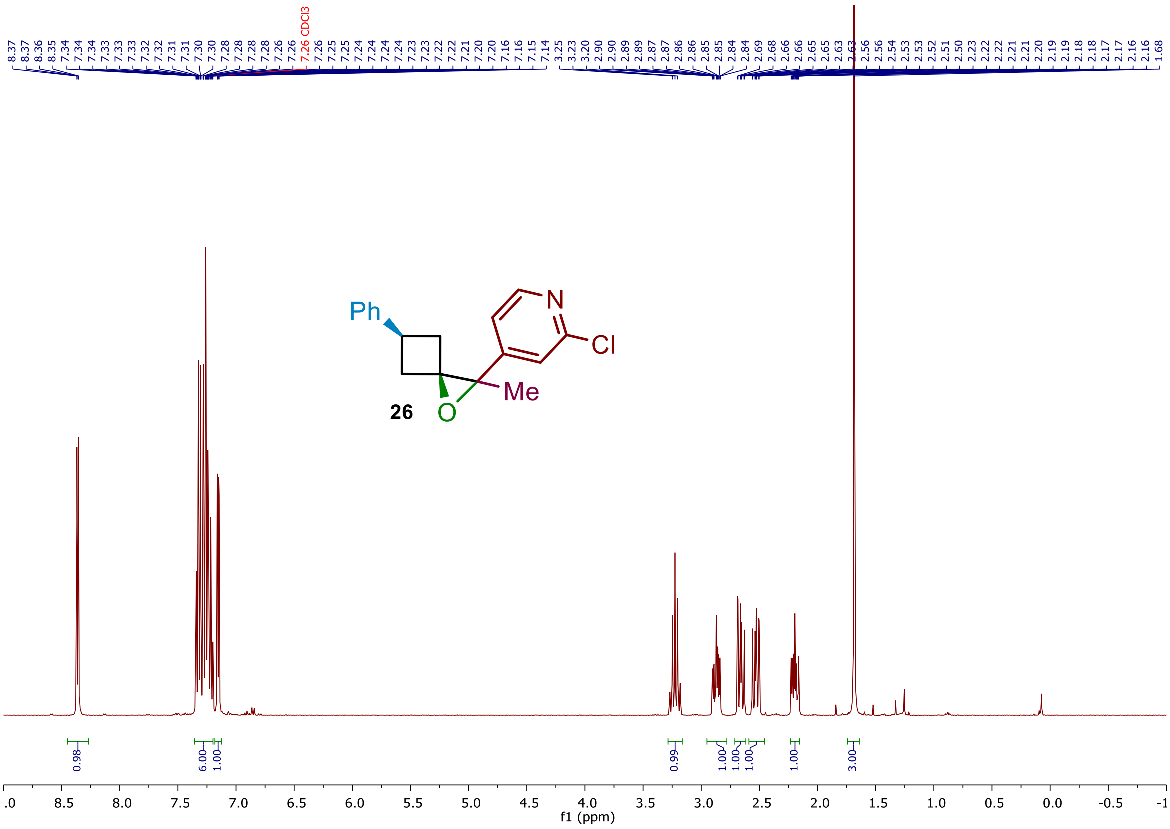

^{13}C NMR (101 MHz, $CDCl_3$) of **26**

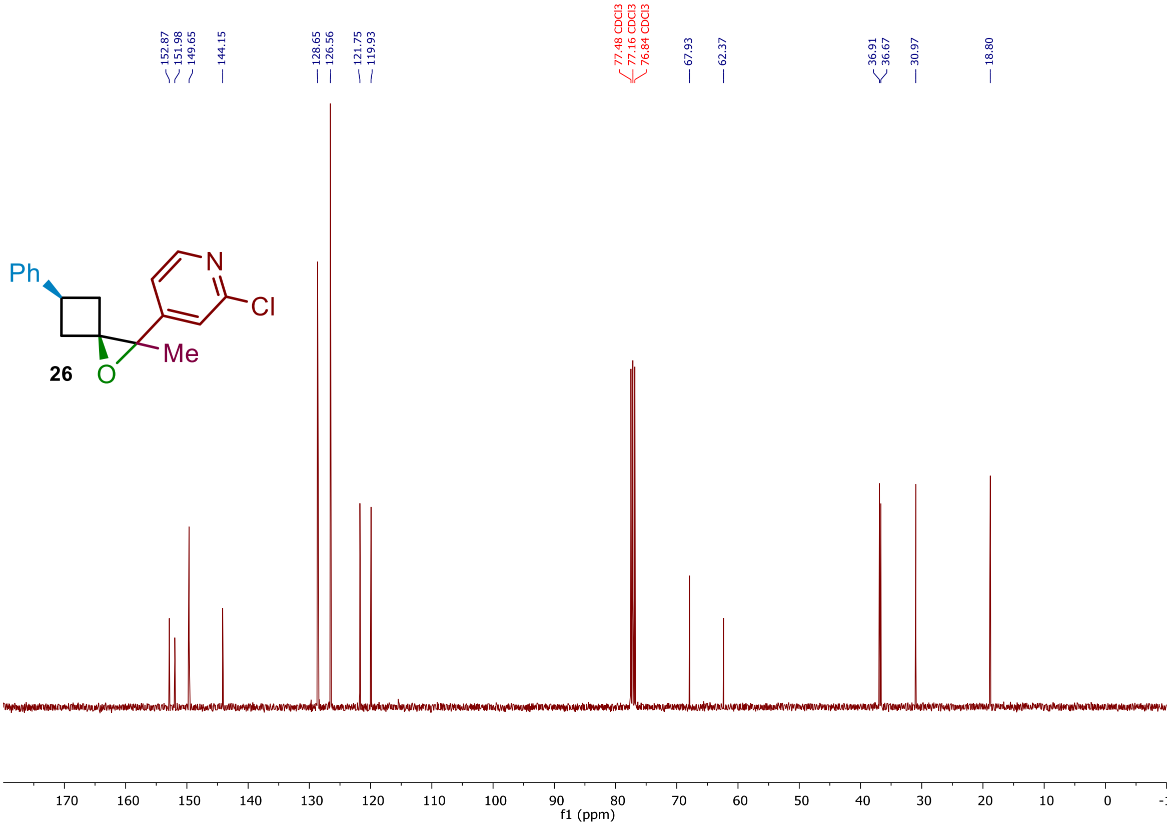

^{1}H NMR (400 MHz, $CDCl_3$) of **27** (*see procedure*)

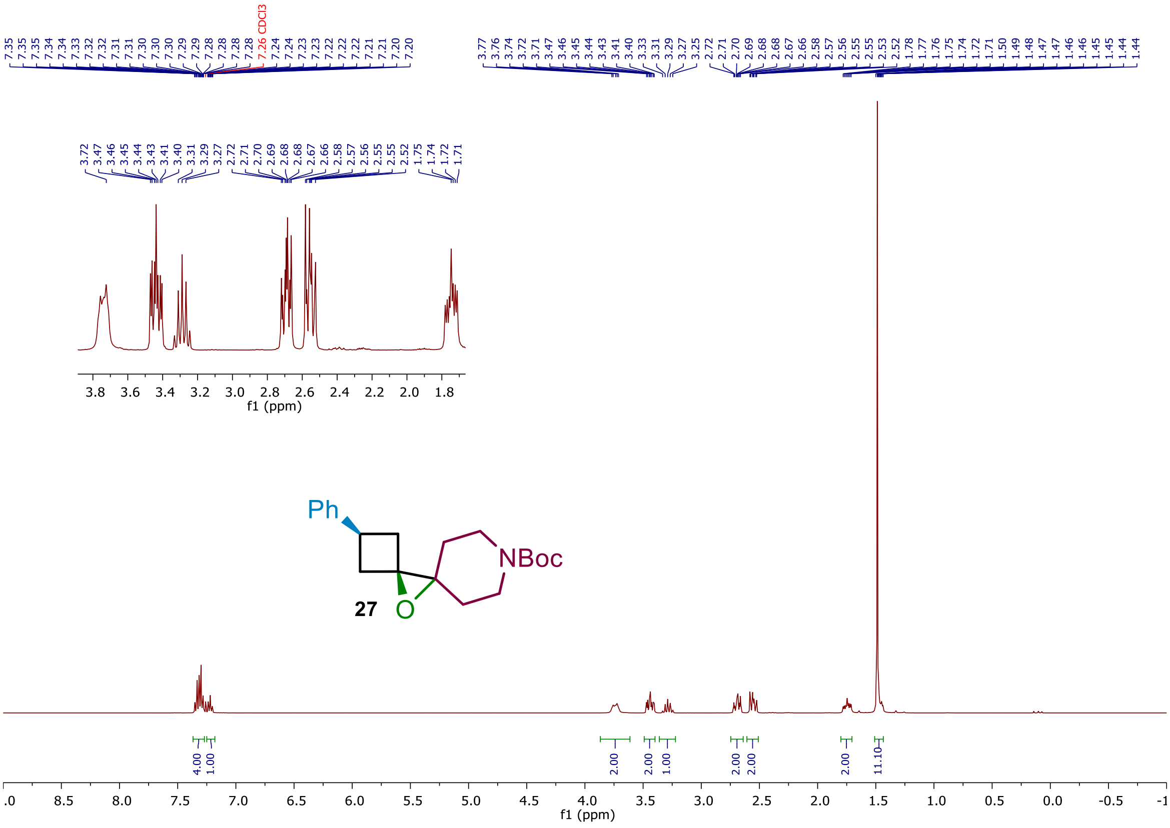

^{13}C NMR (101 MHz, $CDCl_3$) of **27**

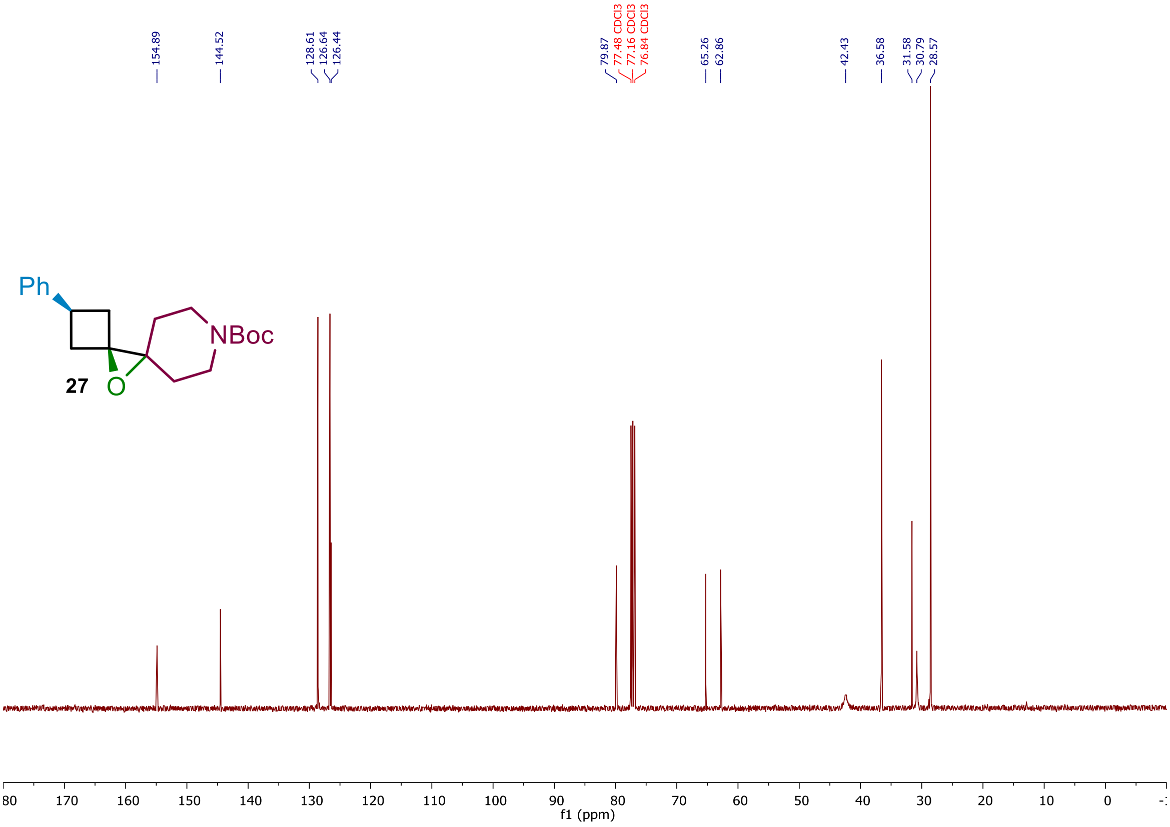

^{1}H NMR (400 MHz, $CDCl_3$) of **28** (*see procedure*)

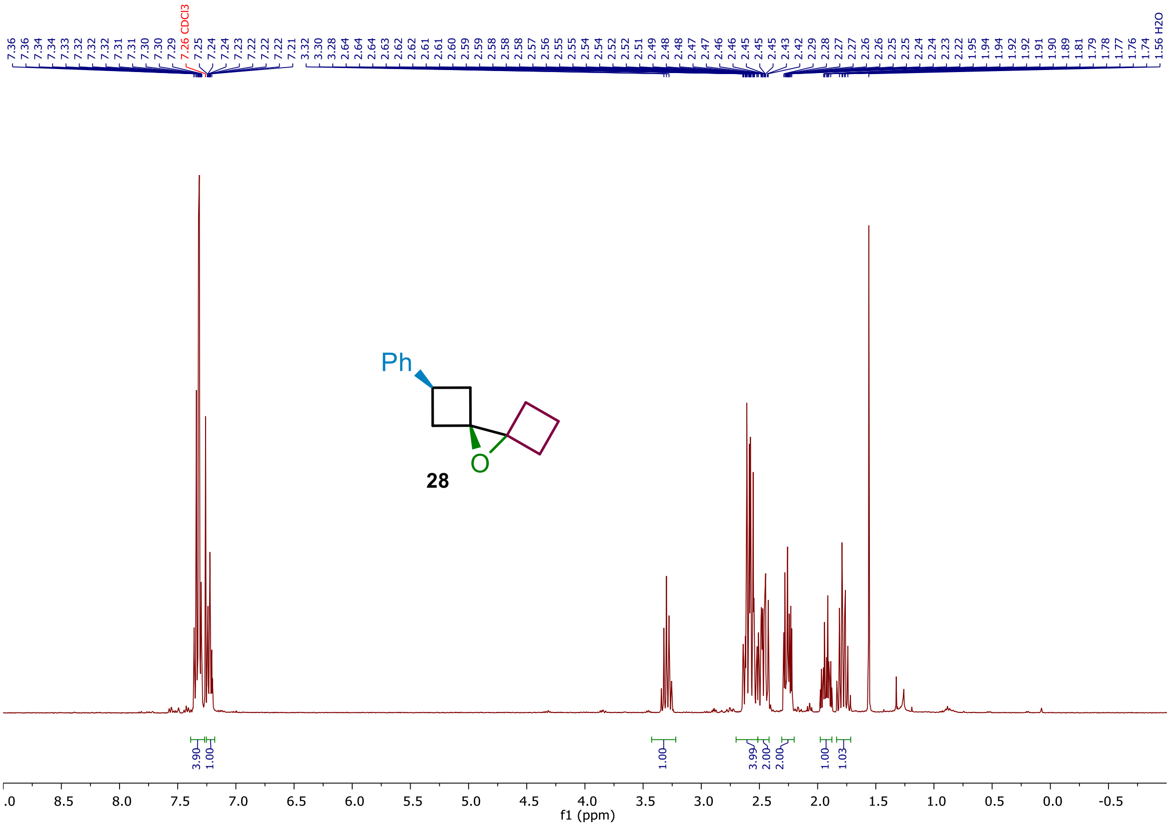

^{13}C NMR (101 MHz, $CDCl_3$) of **28**

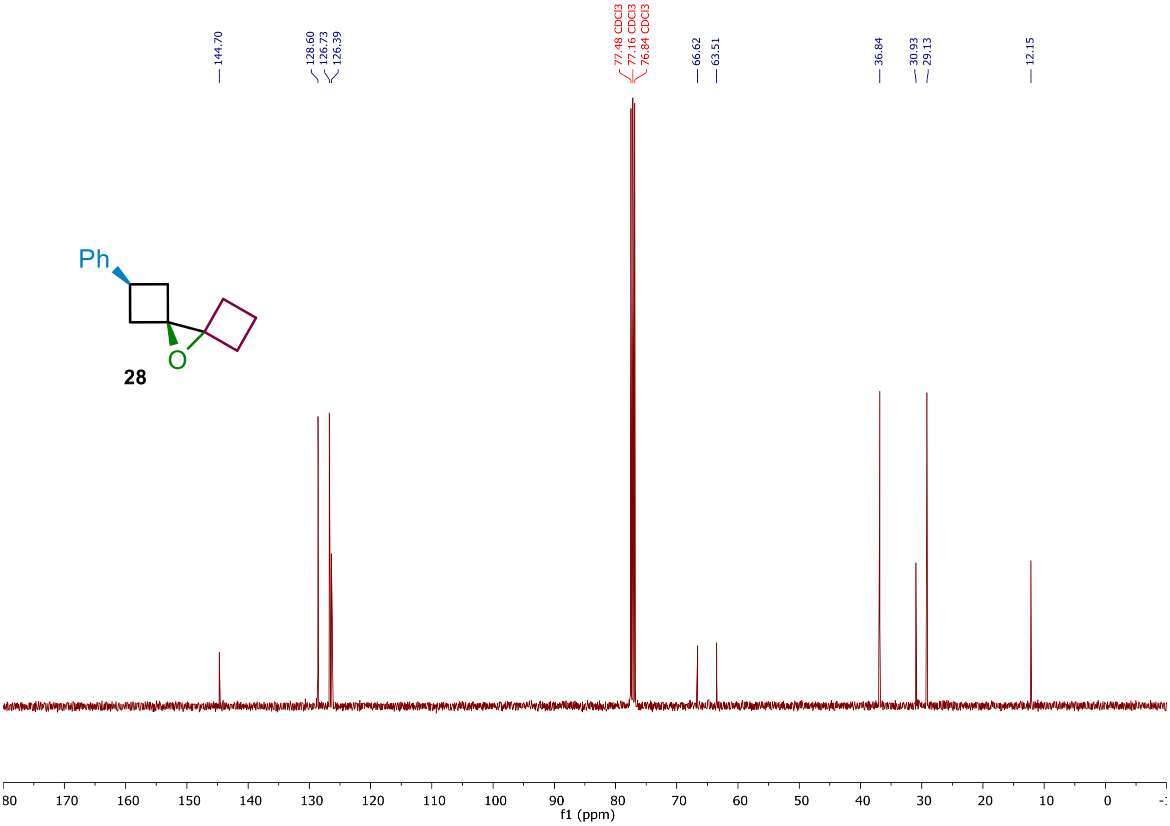

^{1}H NMR (400 MHz, $CDCl_3$) of **29** (*see procedure*)

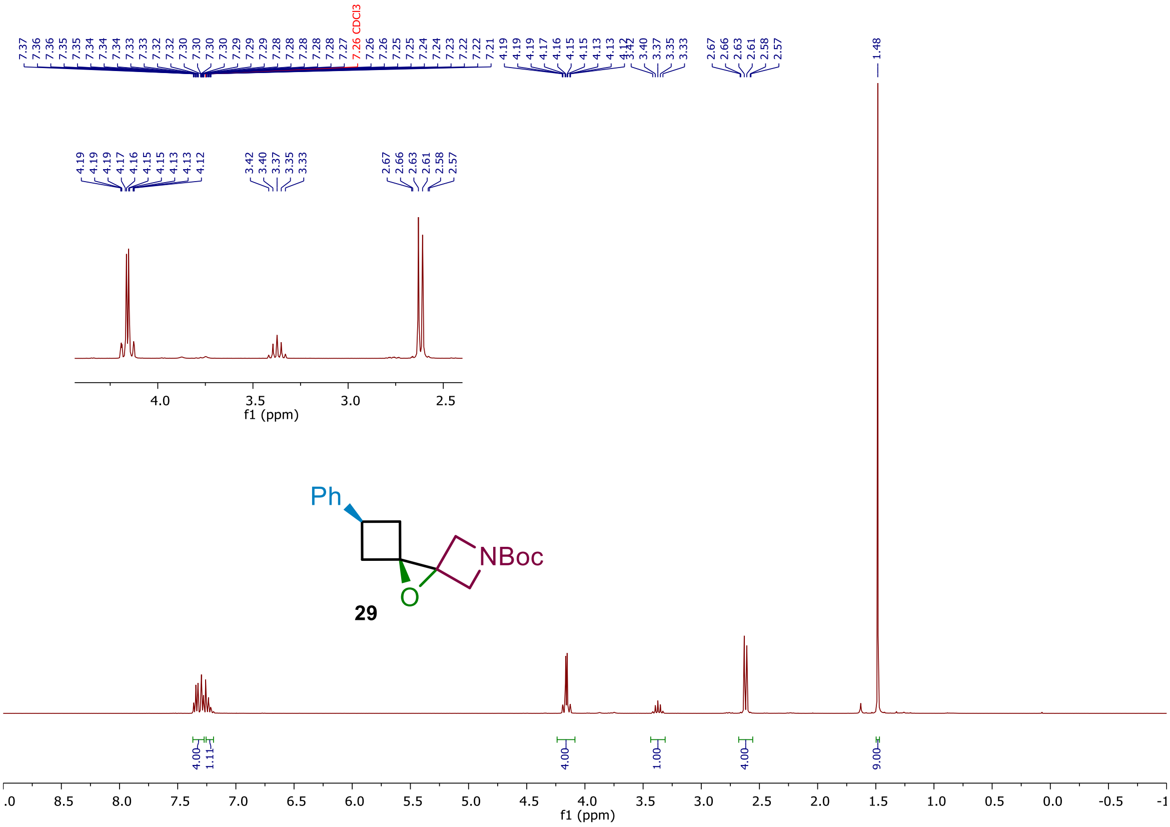

^{13}C NMR (101 MHz, $CDCl_3$) of **29**

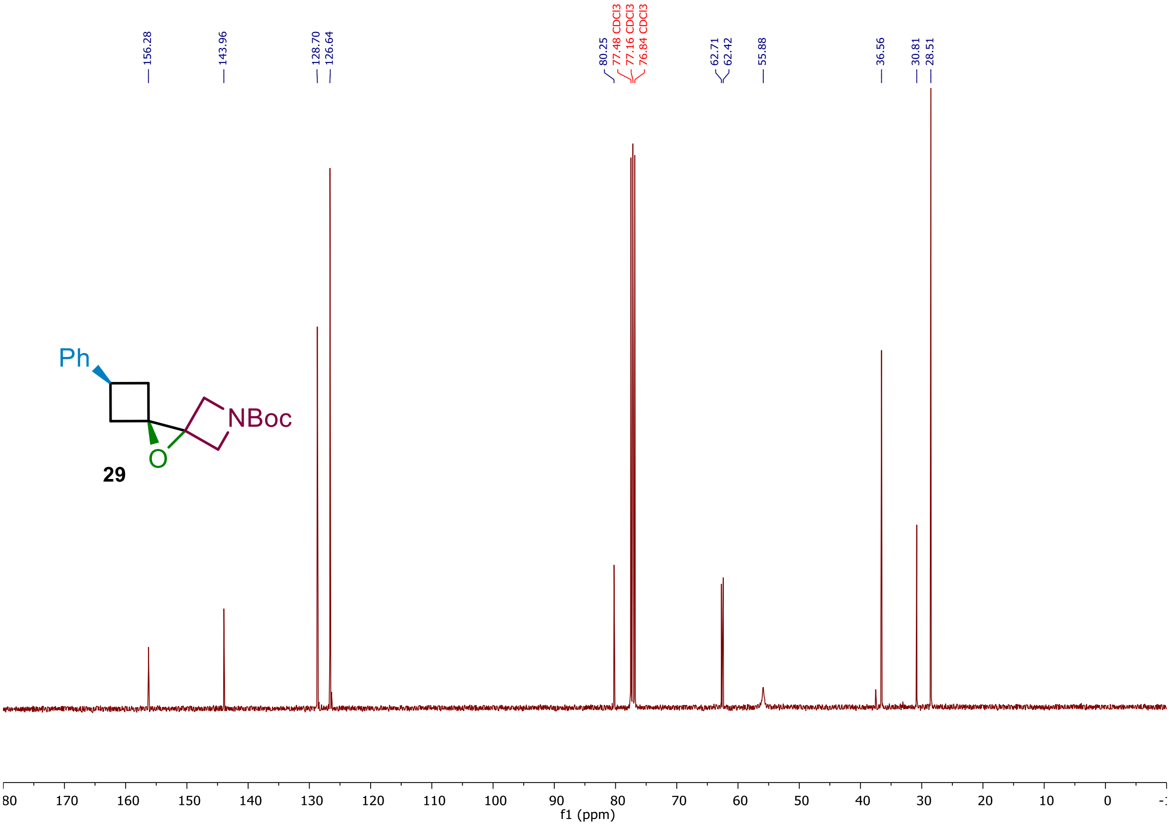

^{1}H NMR (400 MHz, CDCl$_3$) of **30** (*see procedure*)

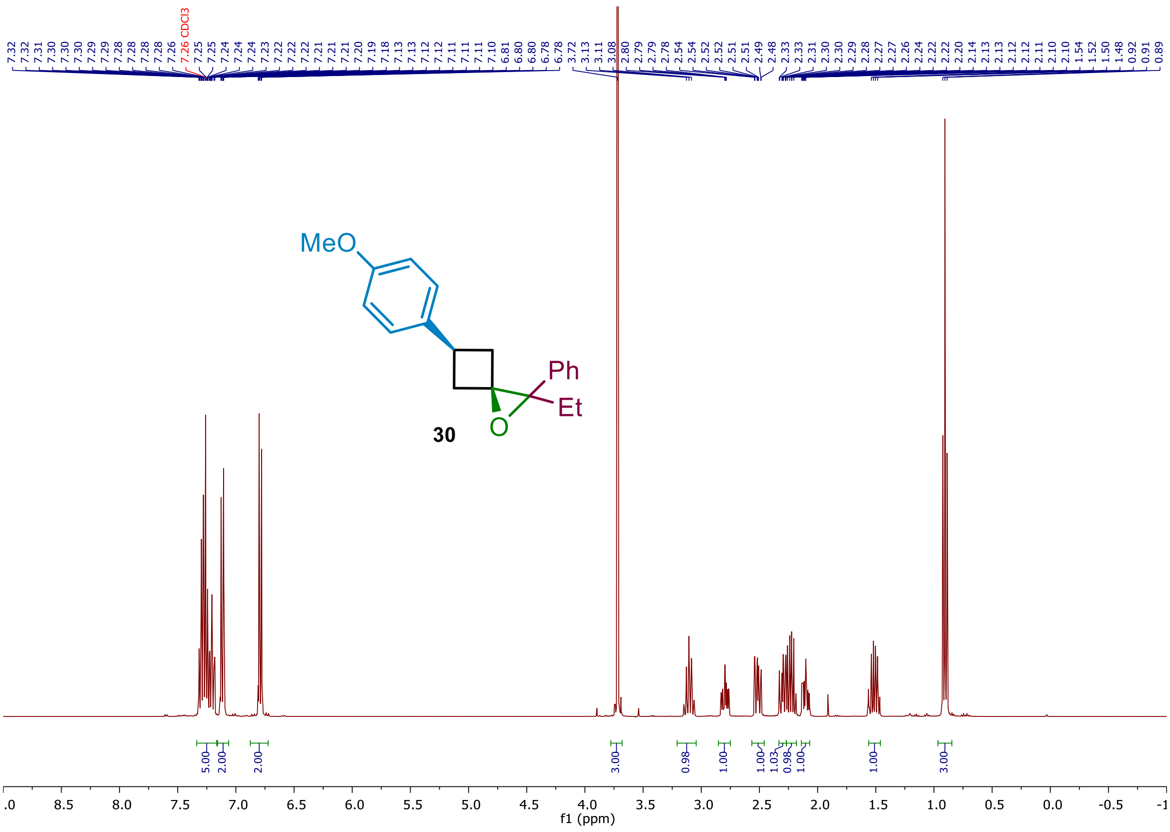

^{13}C NMR (101 MHz, CDCl$_3$) of **30**

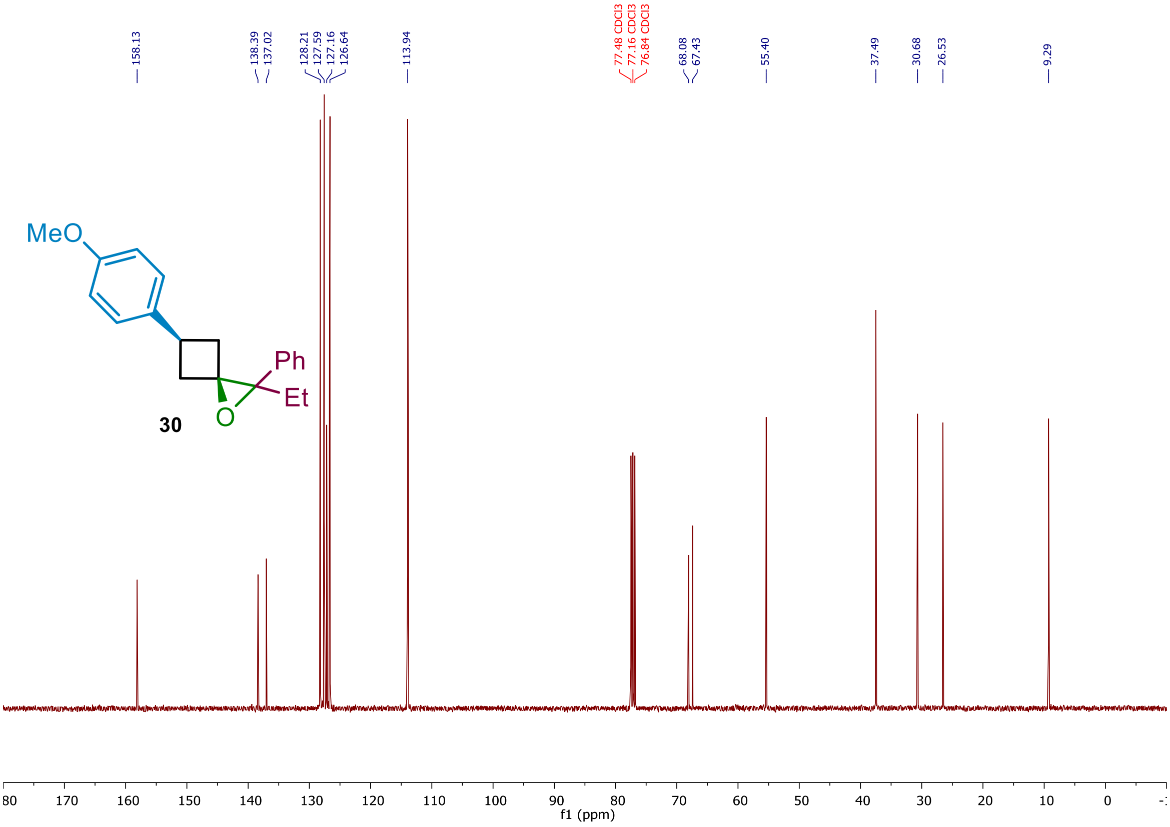

^{1}H NMR (400 MHz, $CDCl_3$) of **31** (*see procedure*)

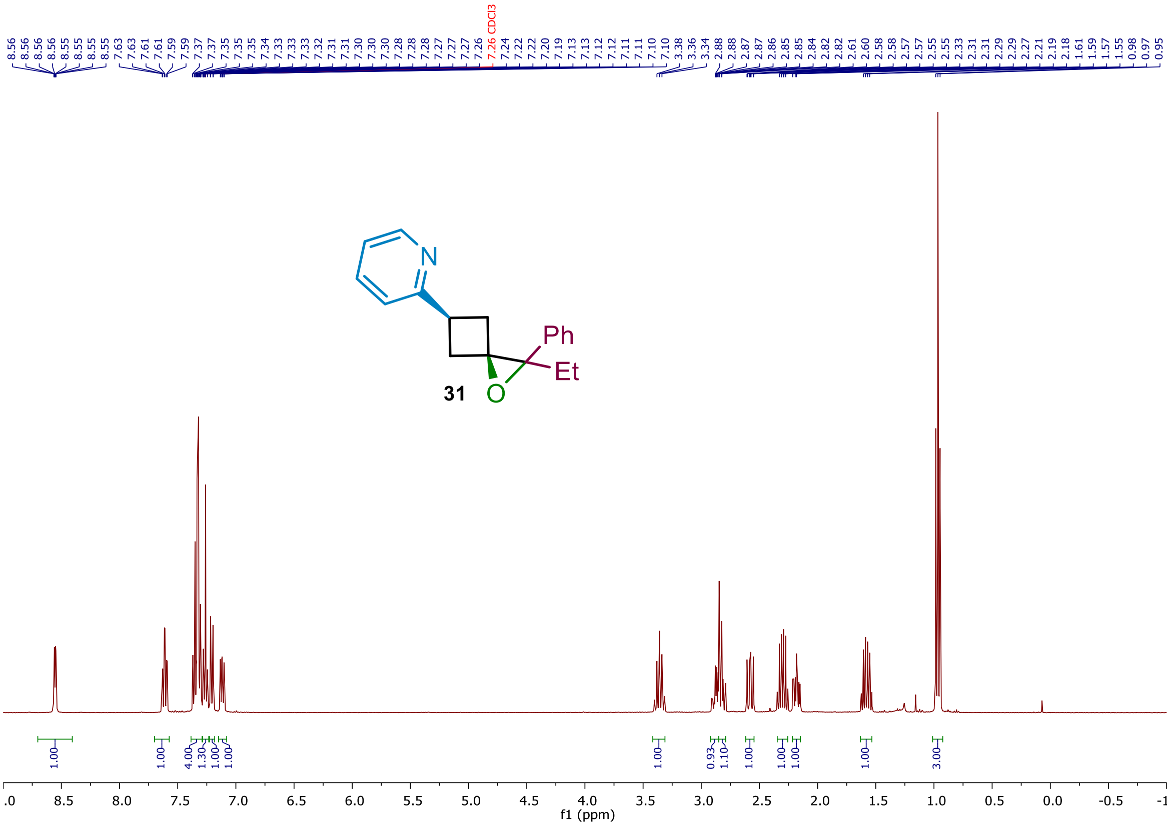

^{13}C NMR (101 MHz, $CDCl_3$) of **31**

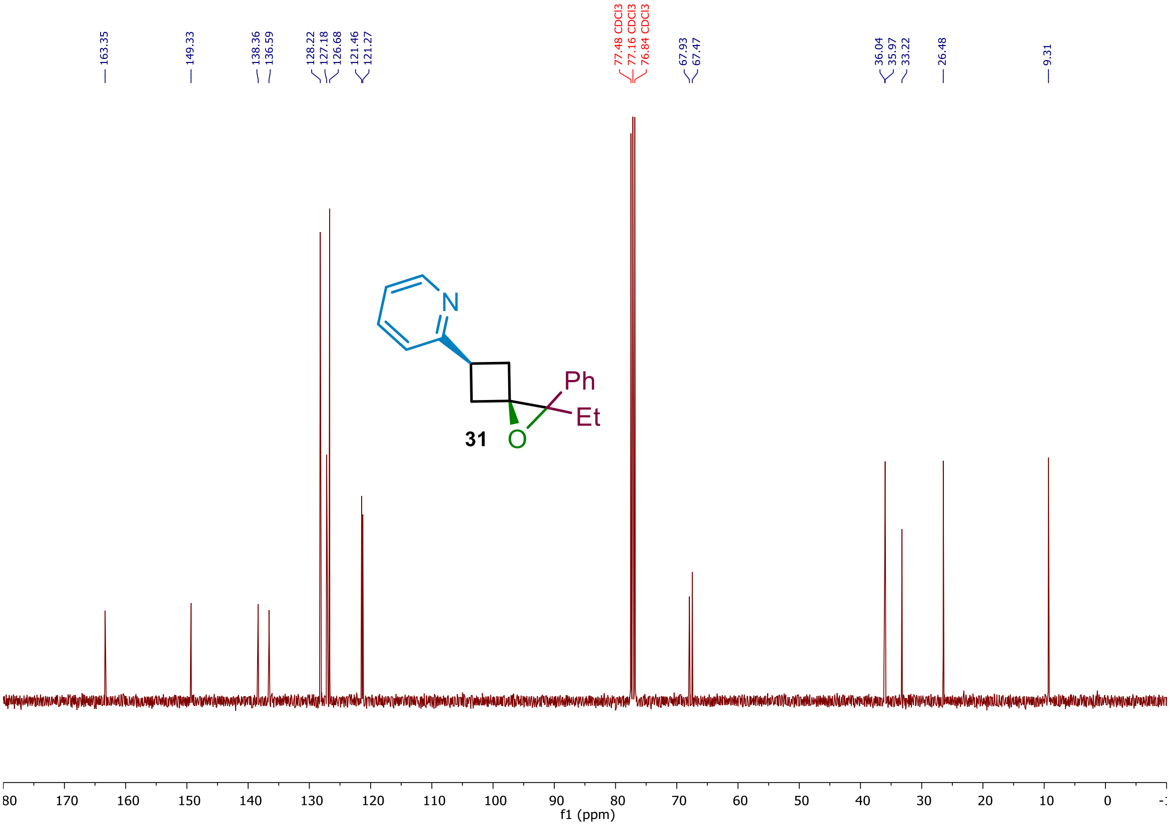

^{1}H NMR (400 MHz, $CDCl_3$) of **32** (*see procedure*)

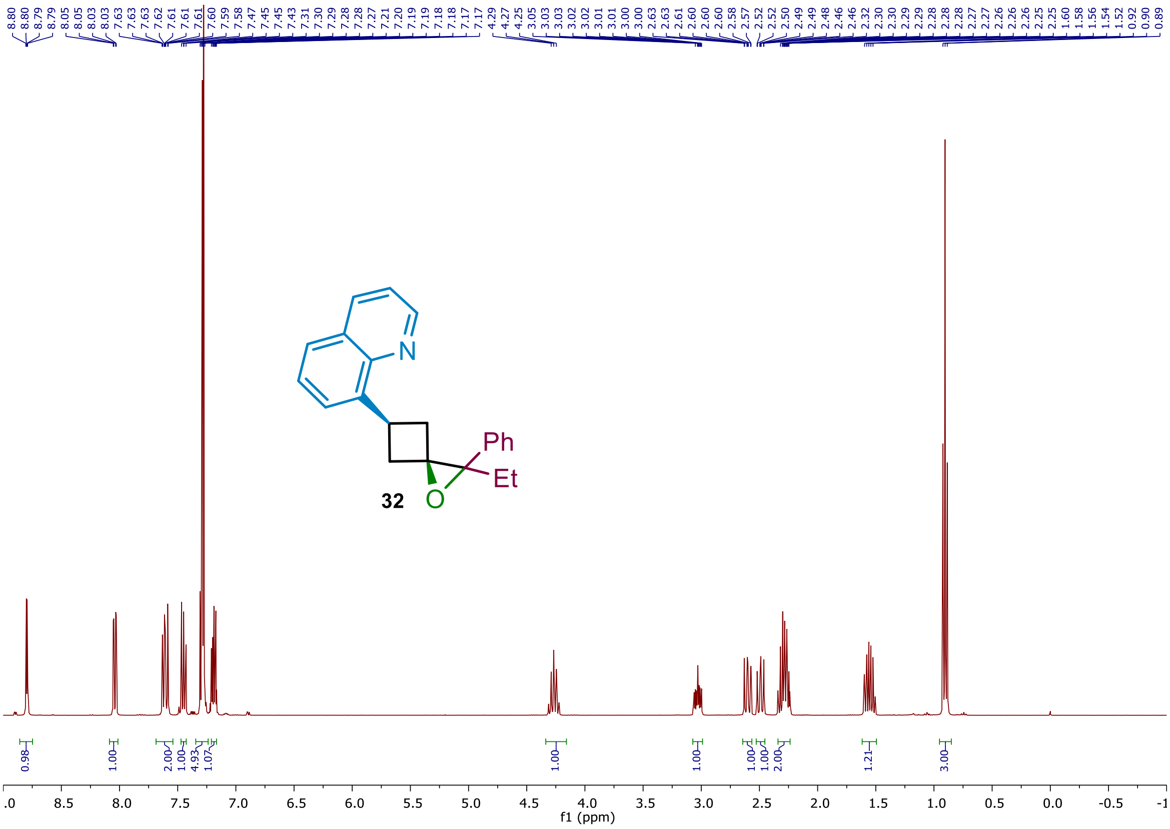

^{13}C NMR (101 MHz, $CDCl_3$) of **32**

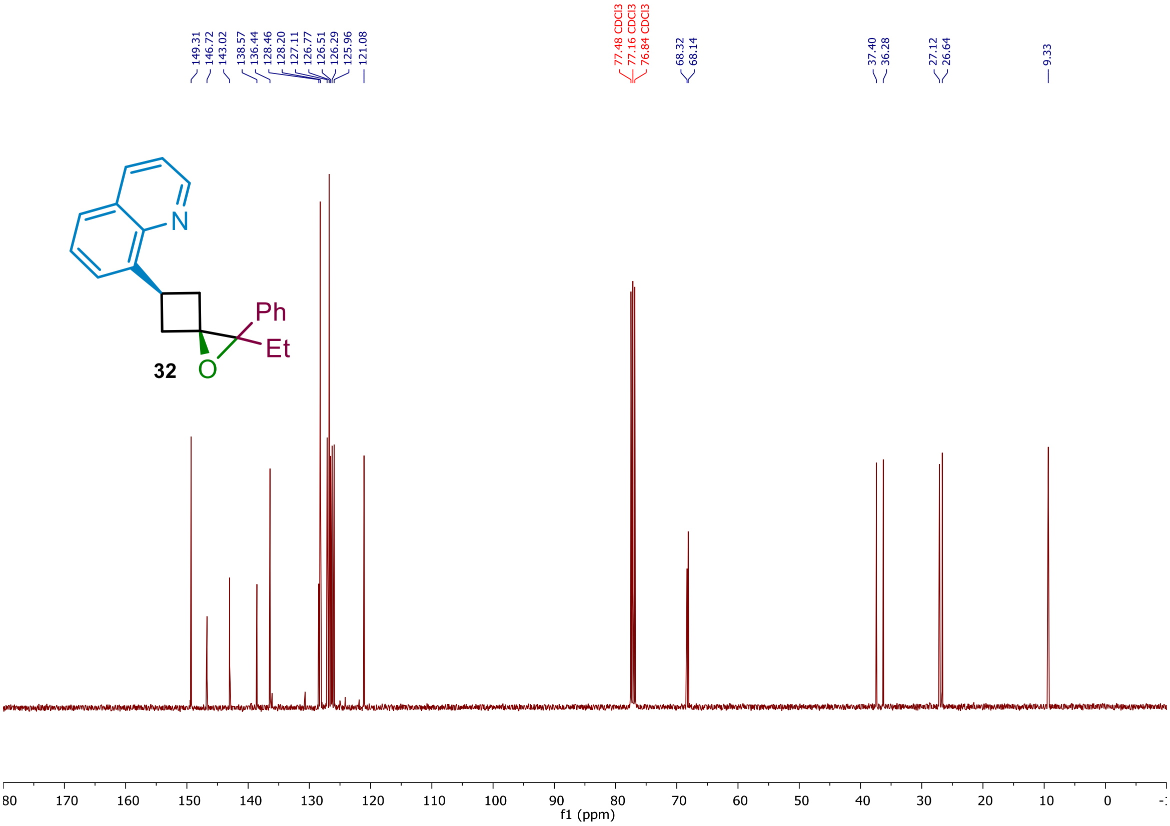

^{1}H NMR (400 MHz, $CDCl_3$) of **33** (*see procedure*)

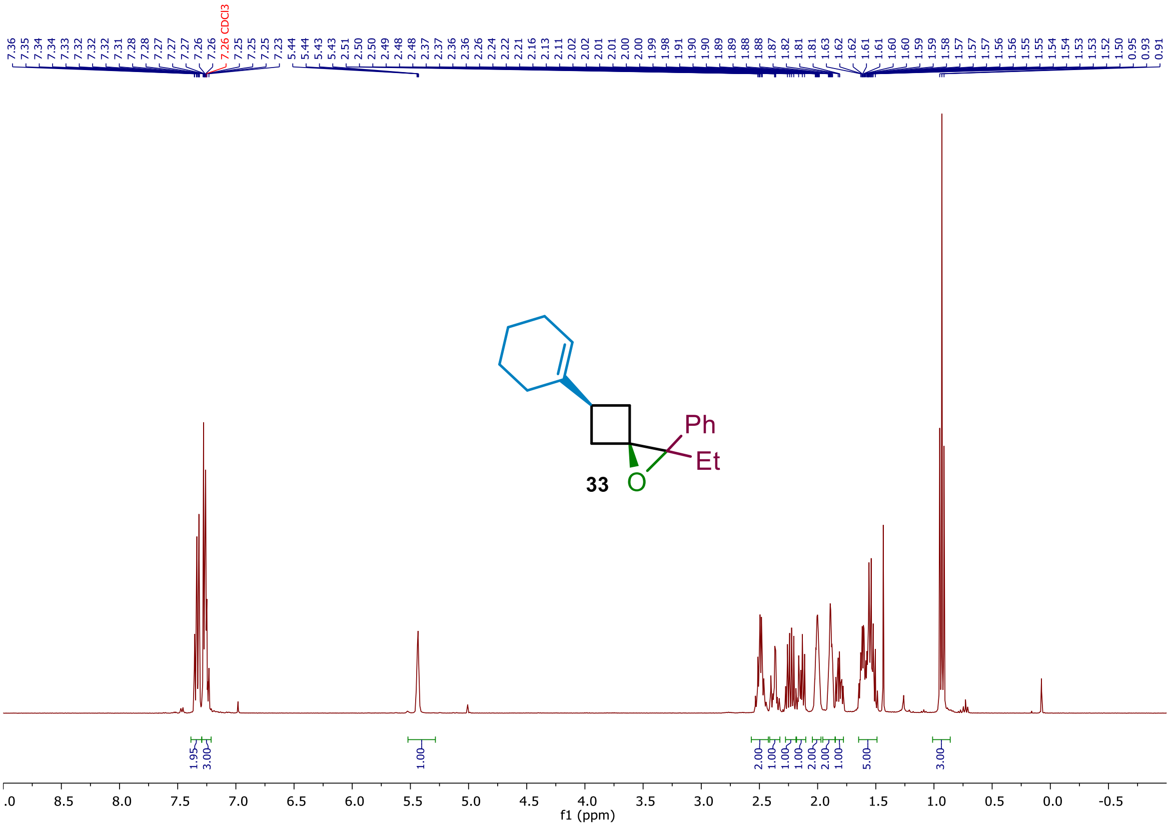

^{13}C NMR (101 MHz, $CDCl_3$) of **33**

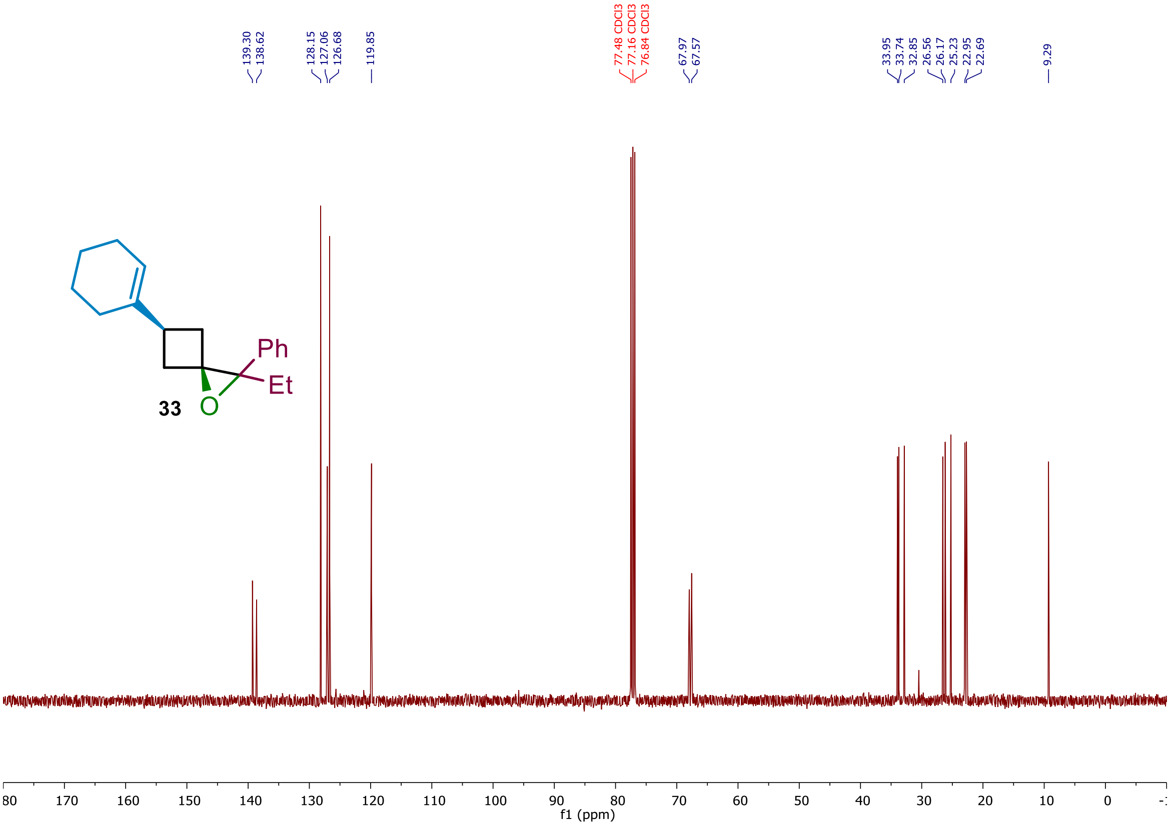

^{1}H NMR (400 MHz, $CDCl_3$) of **34** (*see procedure*)

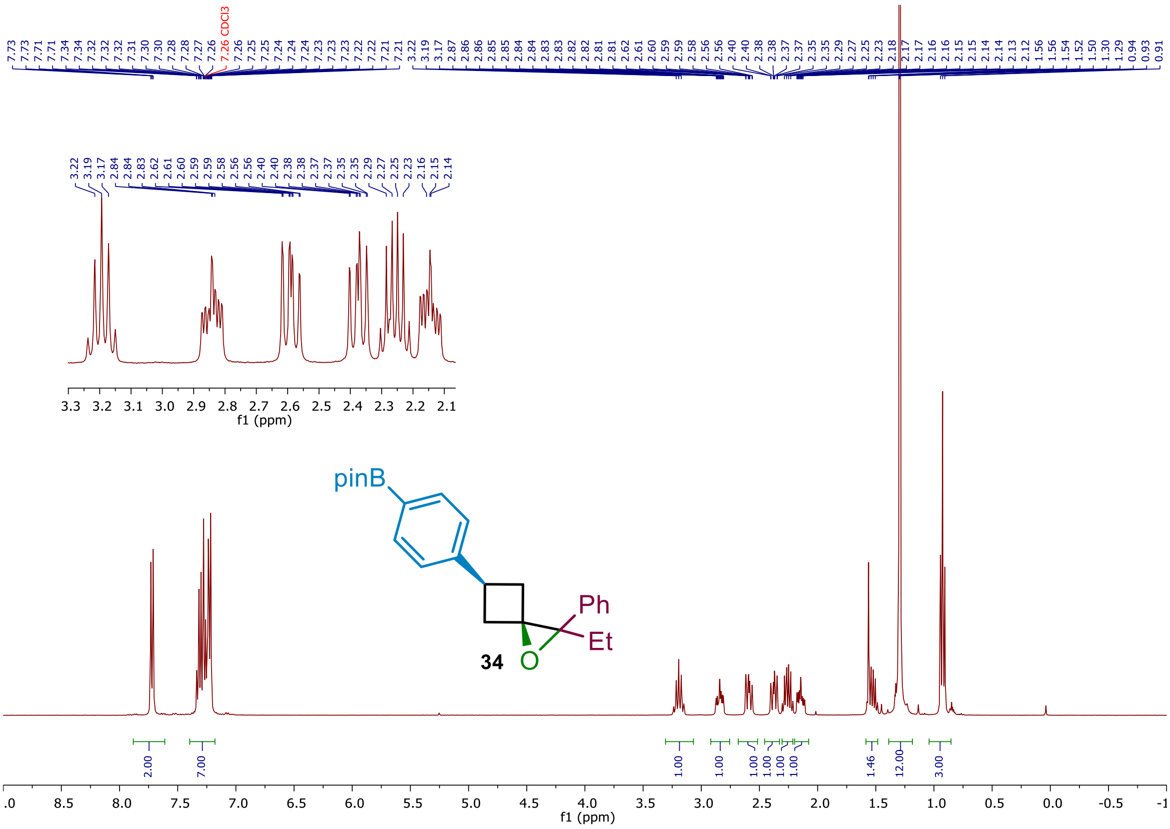

^{13}C NMR (101 MHz, $CDCl_3$) of **34**

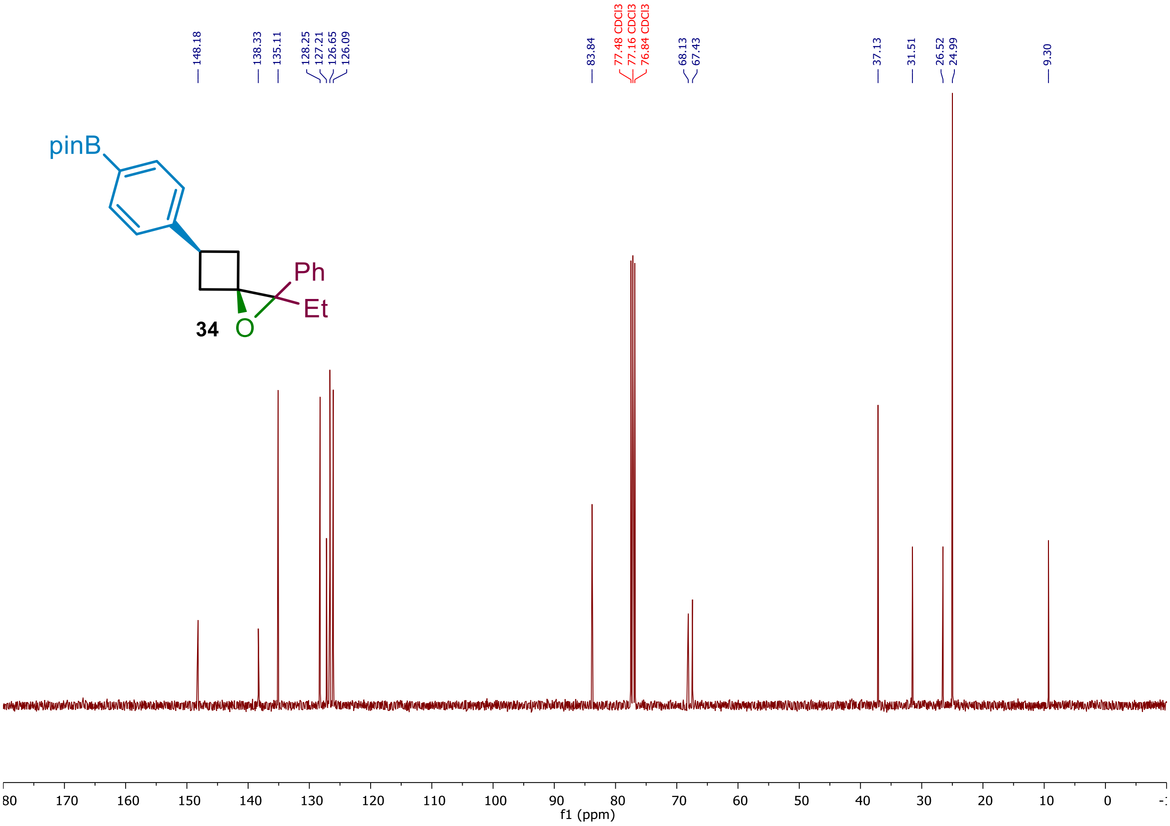

^{11}B NMR (128 MHz, $CDCl_3$) of **34**

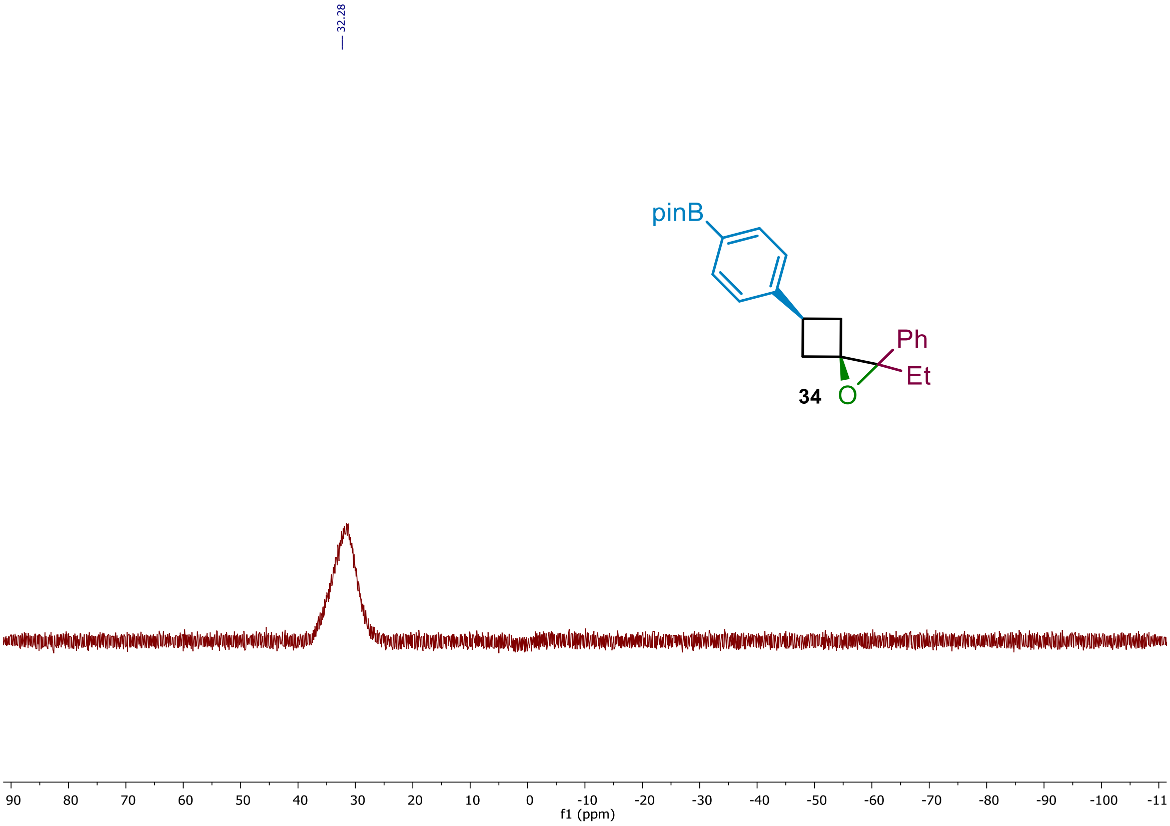

^{1}H NMR (400 MHz, $CDCl_3$) of **35** (*see procedure*)

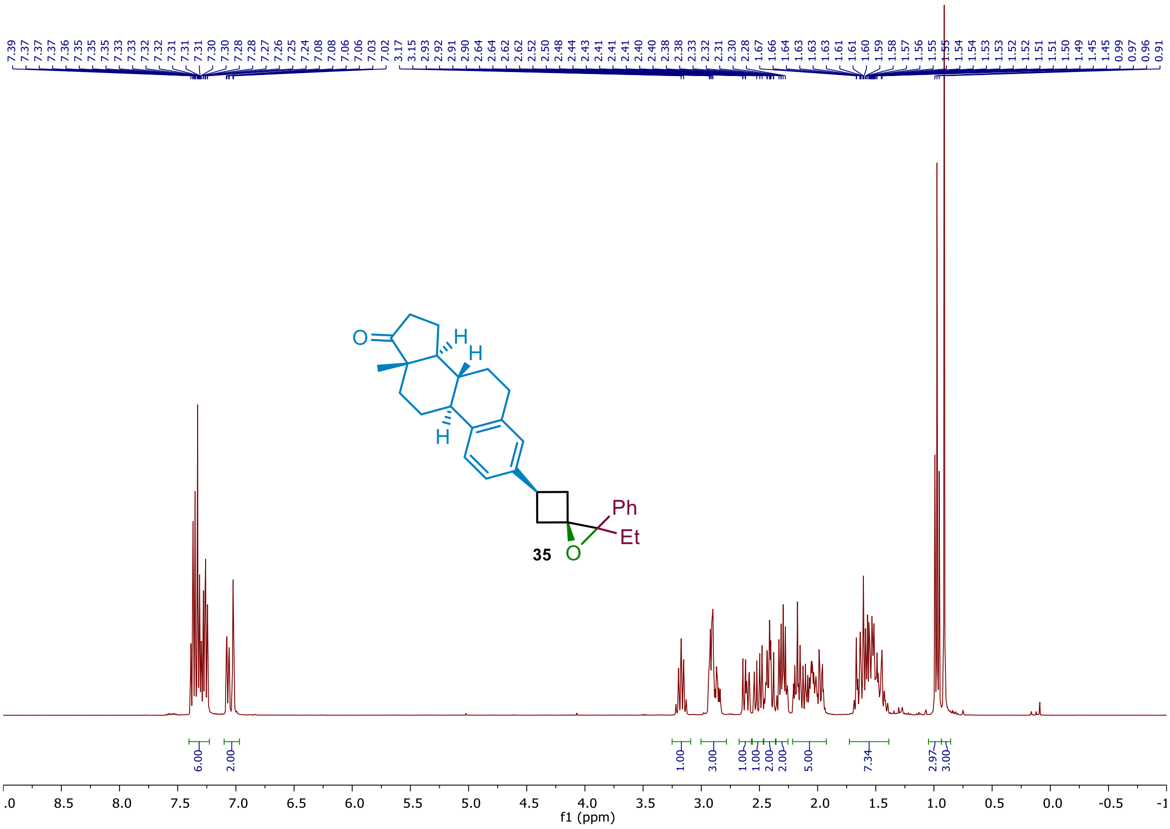

^{13}C NMR (101 MHz, $CDCl_3$) of **35**

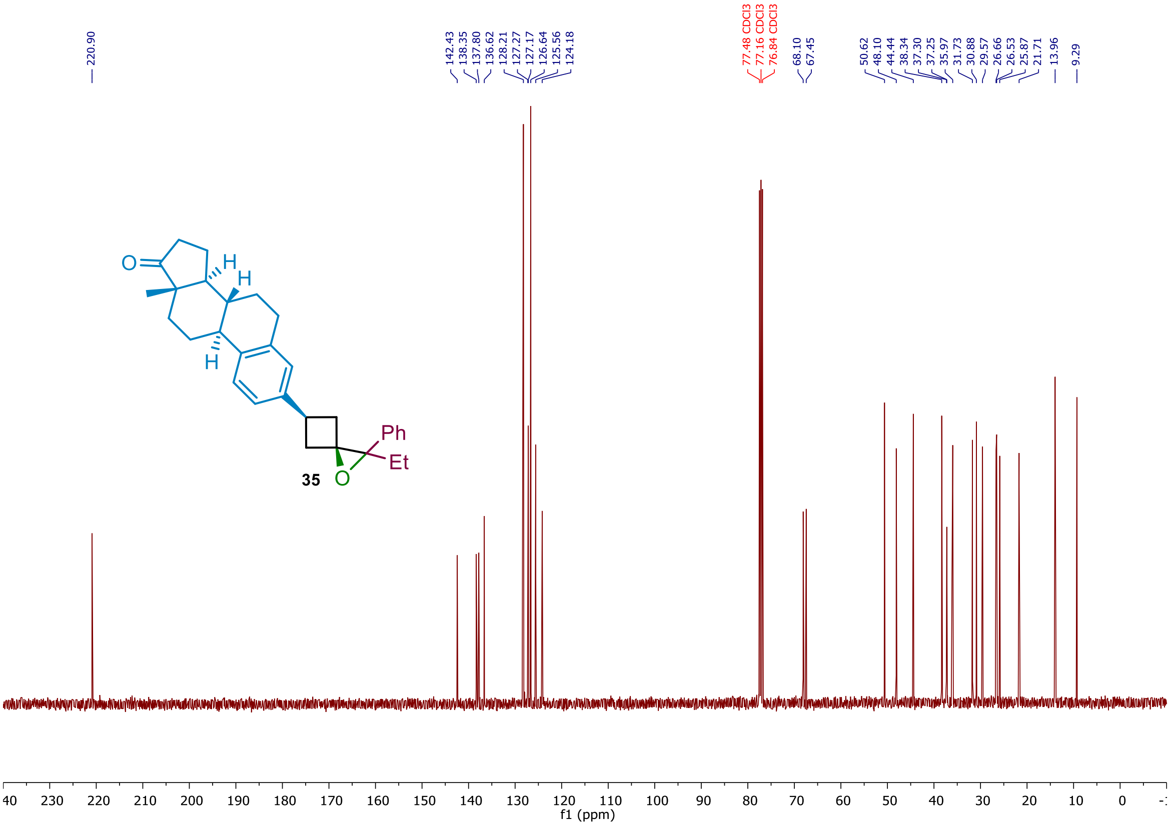

^{1}H NMR (400 MHz, $CDCl_3$) of **36** (*see procedure*)

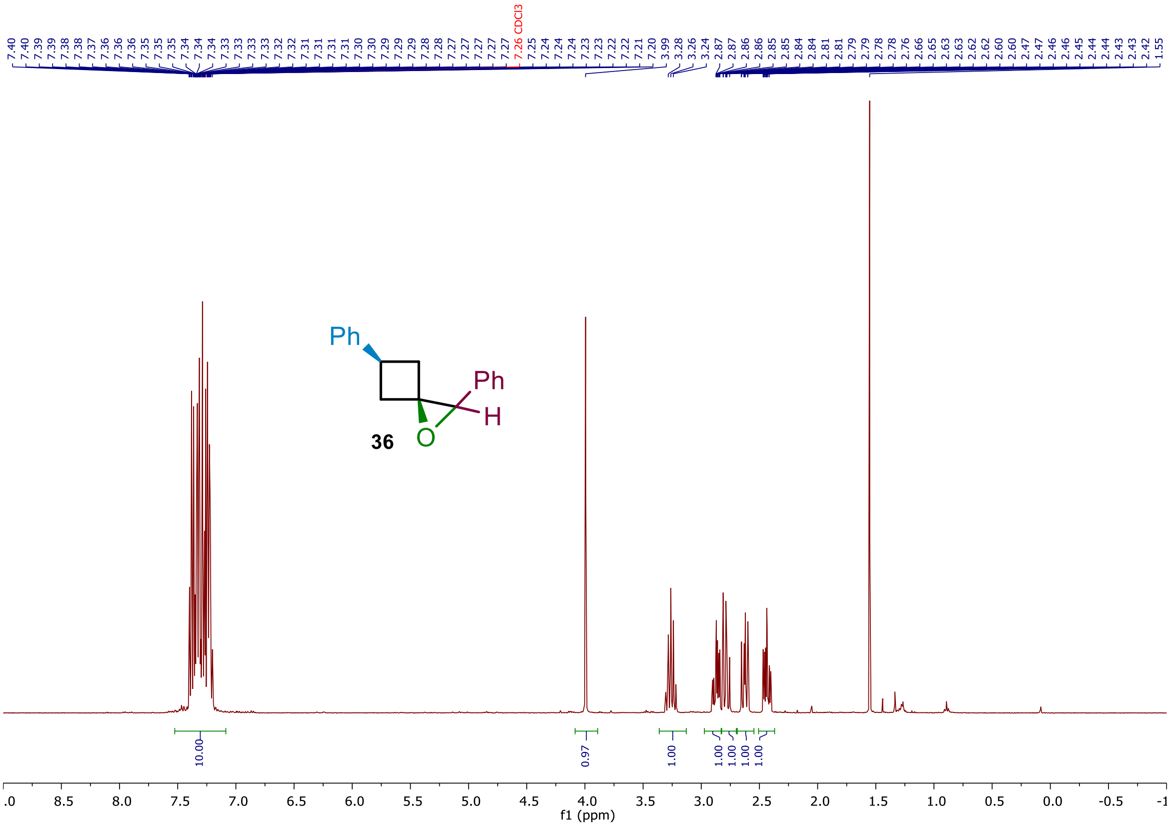

^{13}C NMR (101 MHz, $CDCl_3$) of **36**

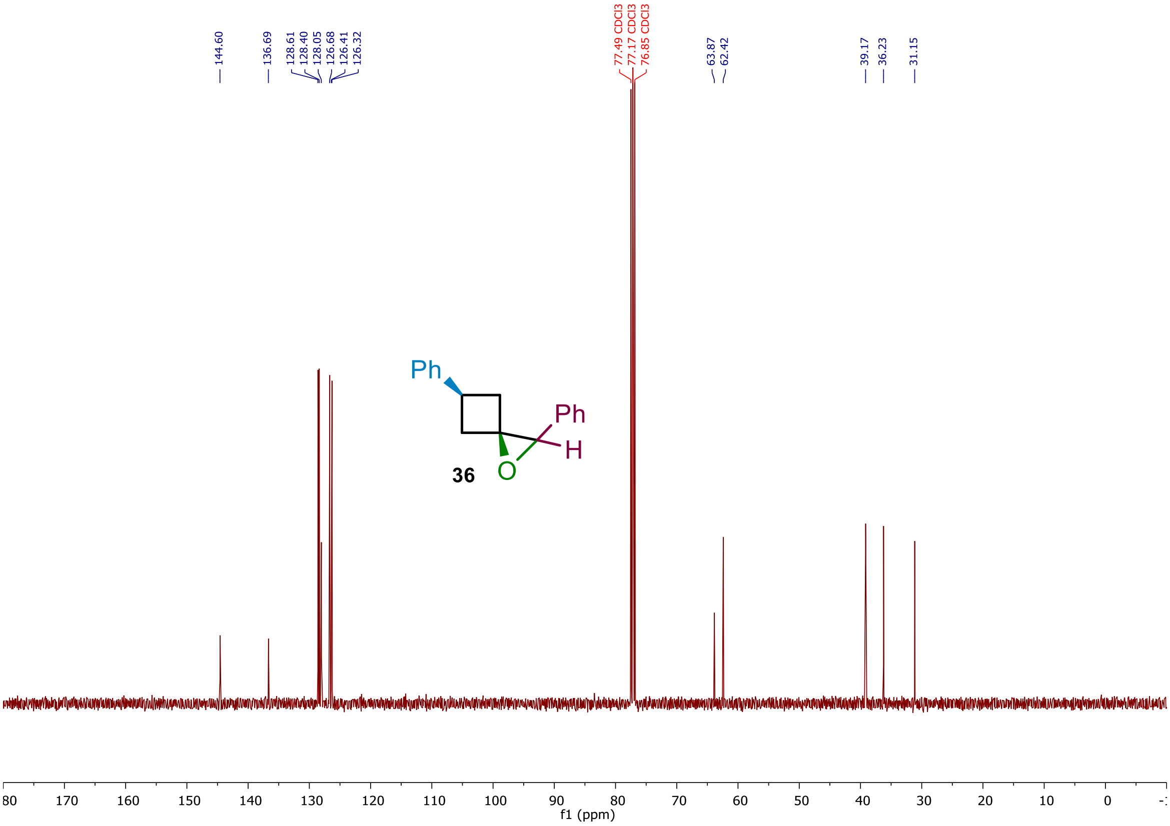

^{1}H NMR (400 MHz, $CDCl_3$) of **37** (*see procedure*)

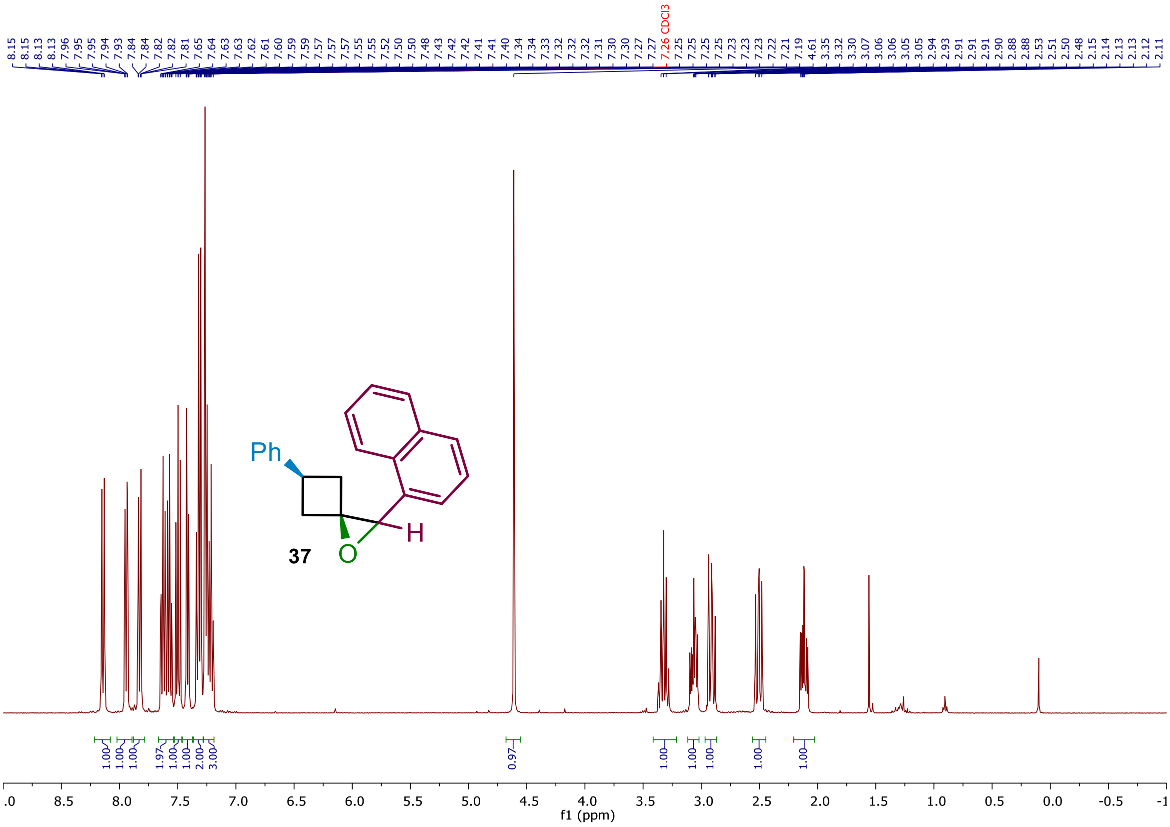

^{13}C NMR (101 MHz, $CDCl_3$) of **37**

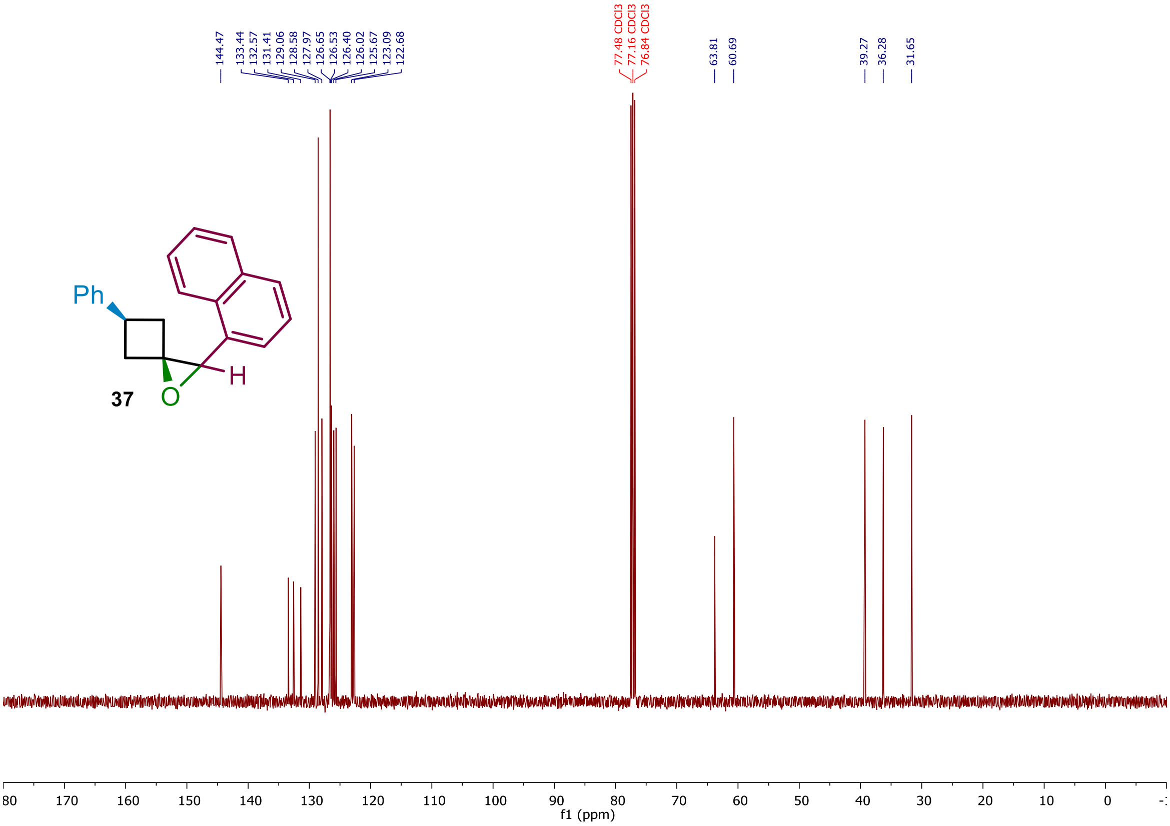

^{1}H NMR (400 MHz, $CDCl_3$) of **38** (*see procedure*)

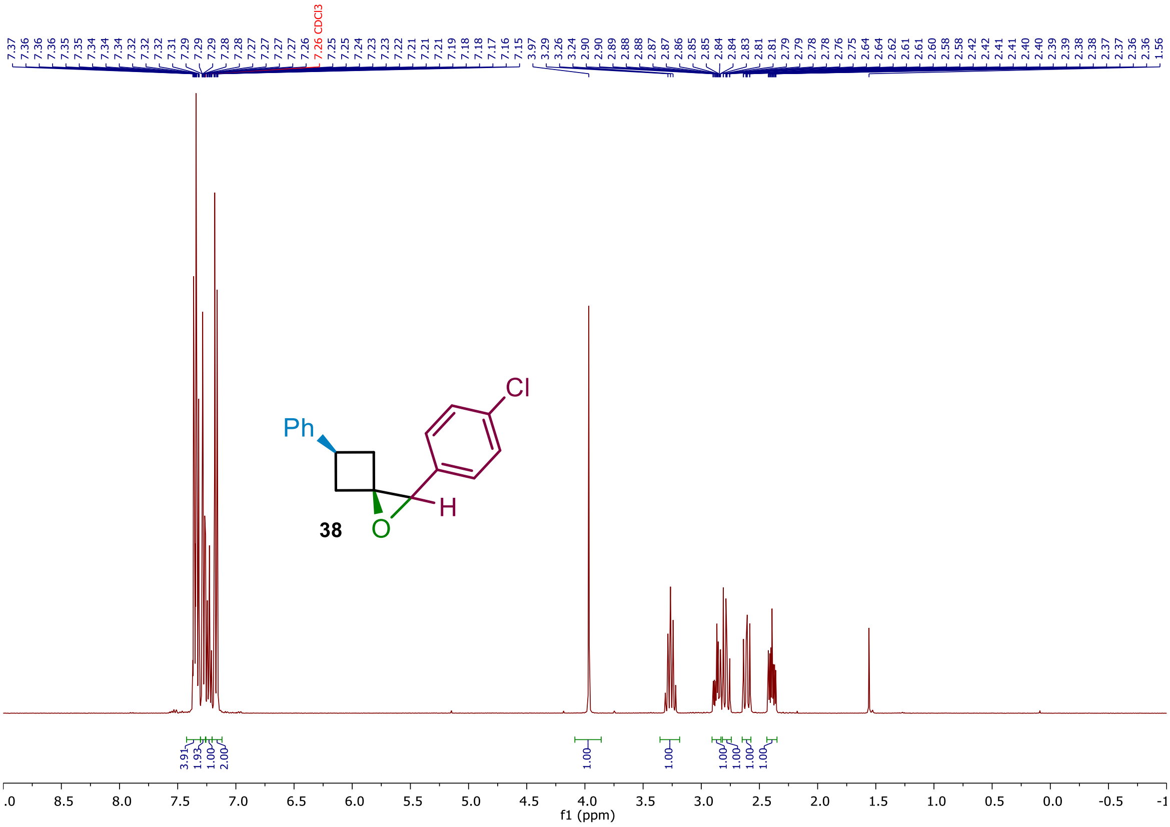

^{13}C NMR (101 MHz, $CDCl_3$) of **38**

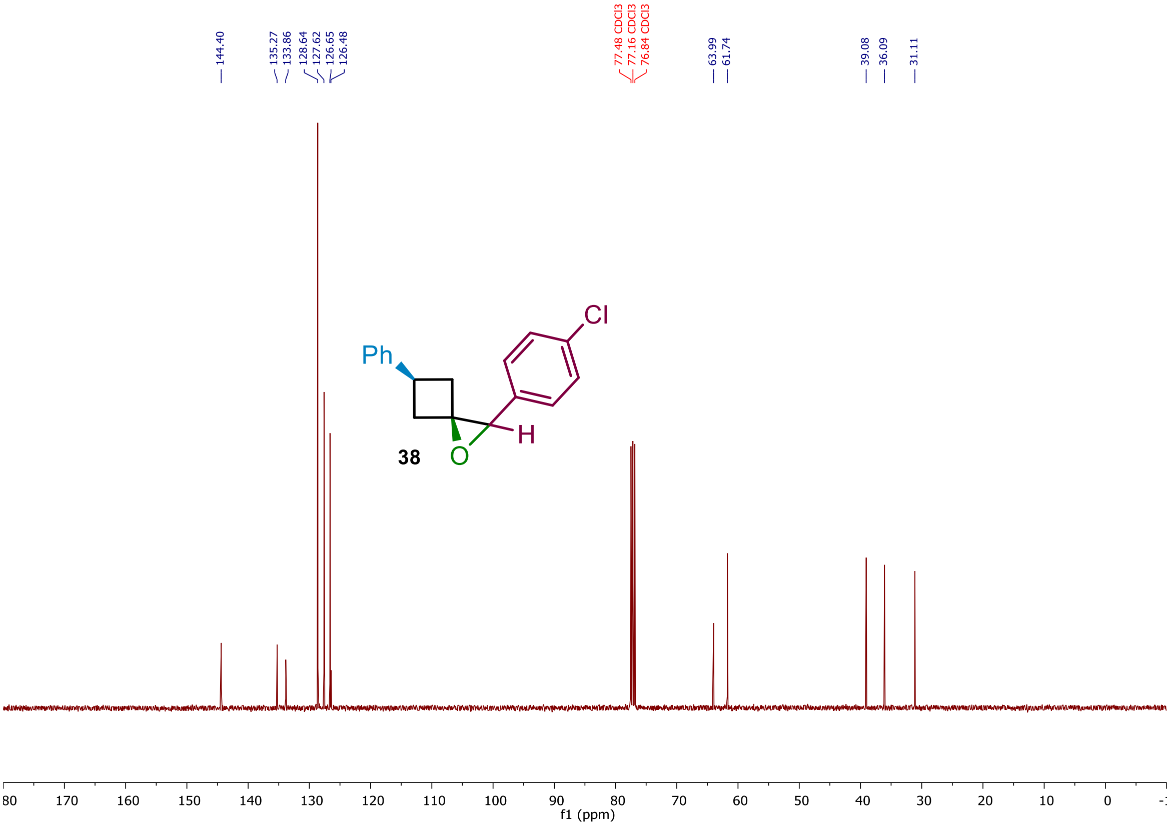

^1H NMR (400 MHz, $CDCl_3$) of **39** (*see procedure*)

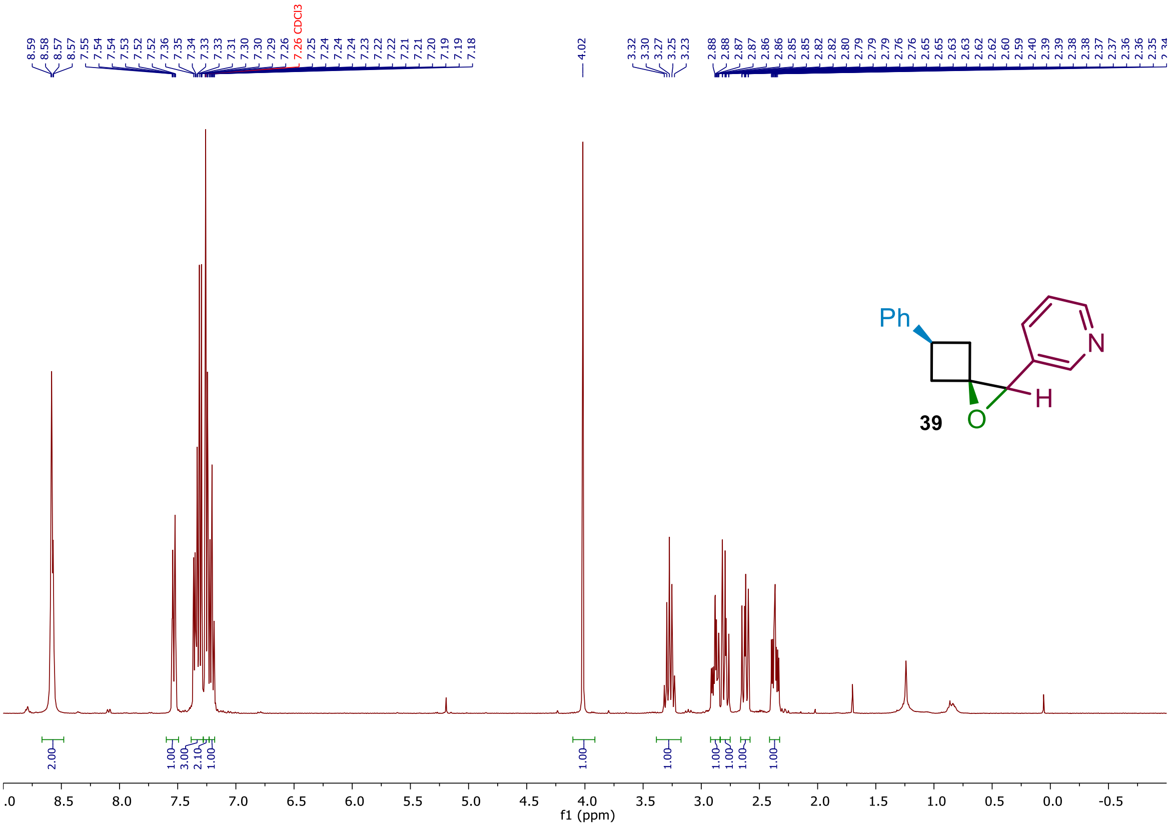

^{13}C NMR (101 MHz, $CDCl_3$) of **39**

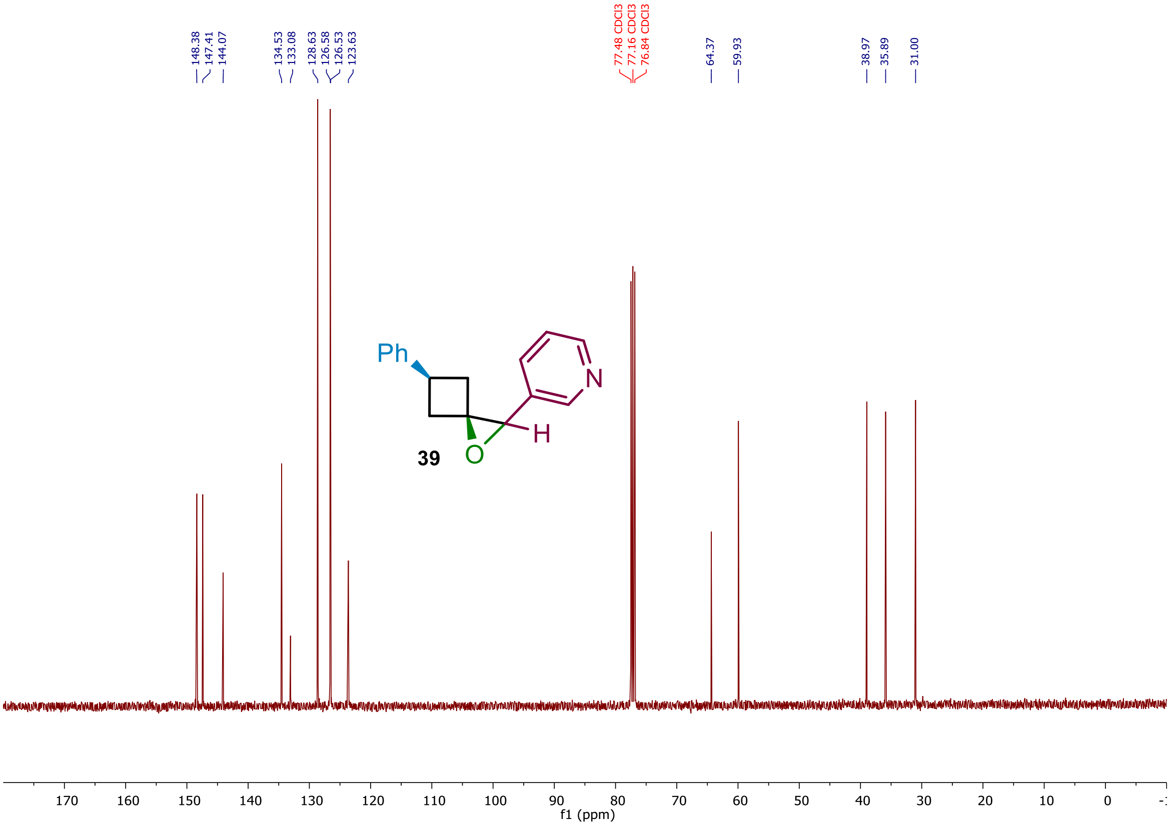

^{1}H NMR (400 MHz, $CDCl_3$) of **40** (*see procedure*)

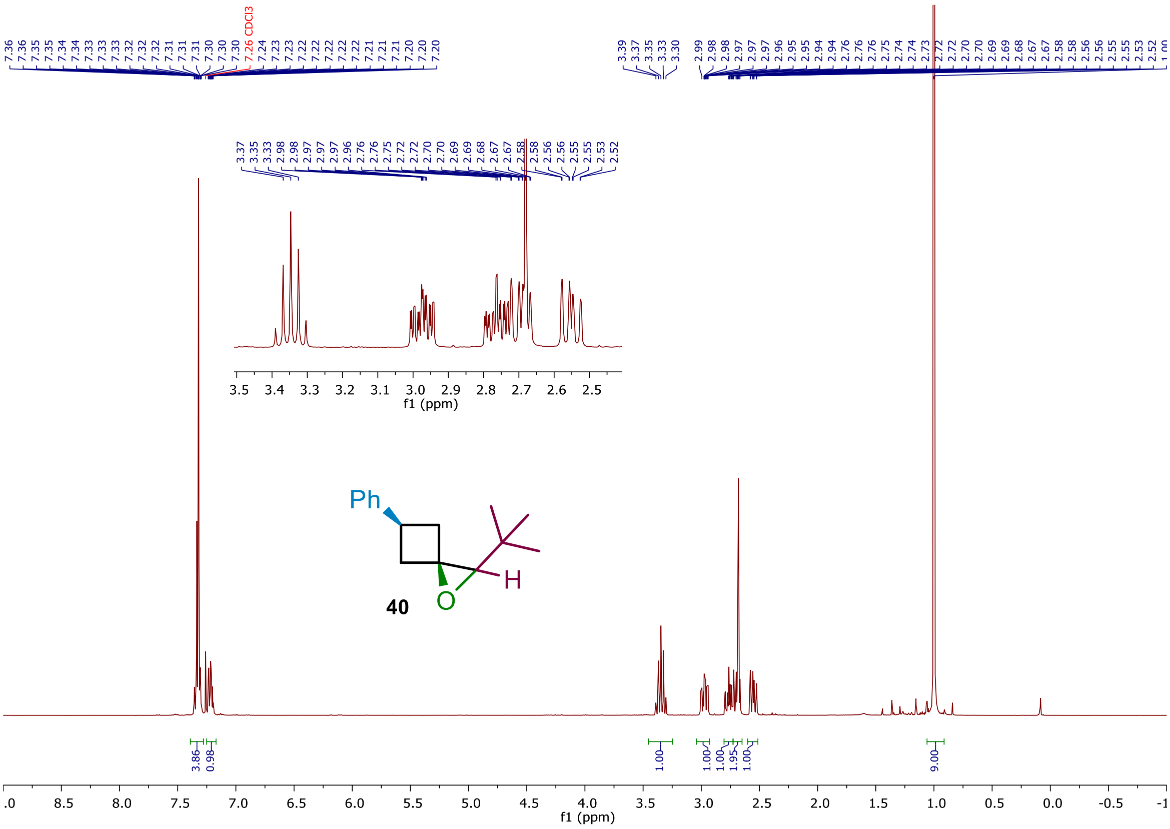

^{13}C NMR (101 MHz, $CDCl_3$) of **40**

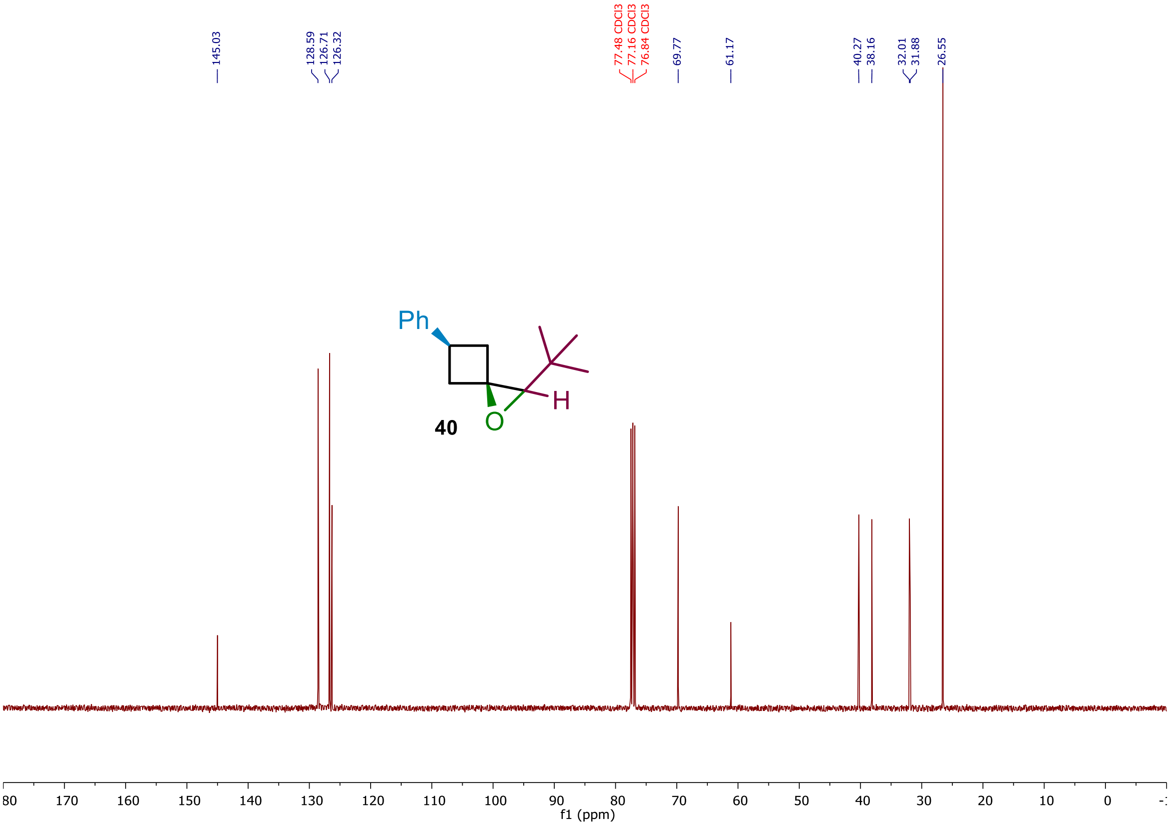

^{1}H NMR (400 MHz, $CDCl_3$) of **41** (*see procedure*)

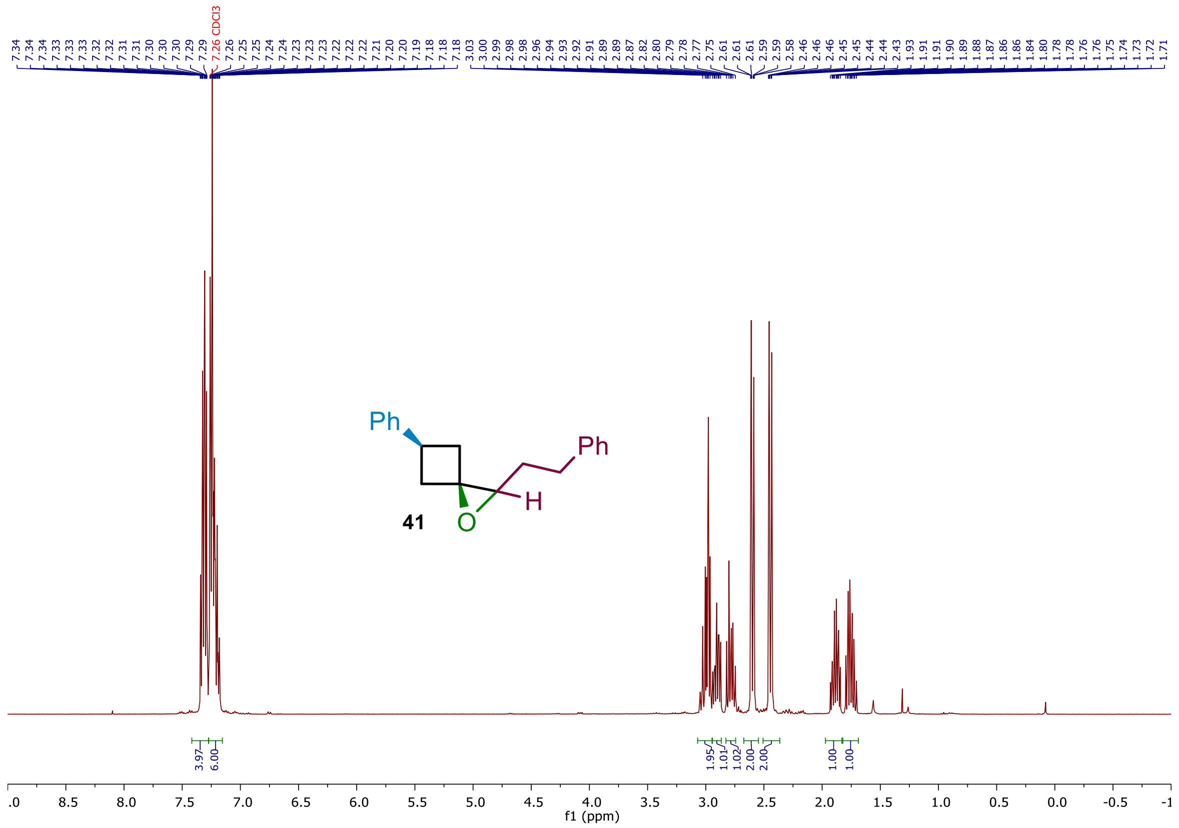

^{13}C NMR (101 MHz, $CDCl_3$) of **41**

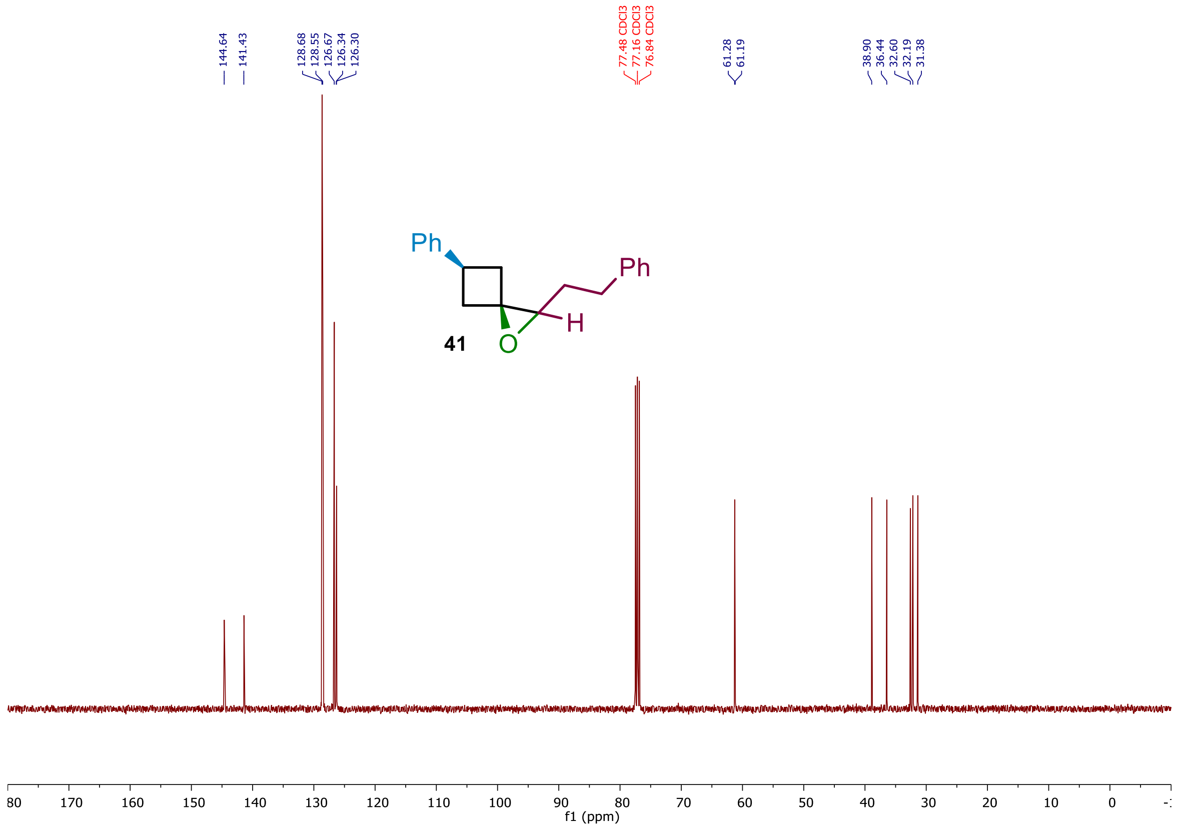

^{1}H NMR (400 MHz, $CDCl_3$) of **42** (*see procedure*)

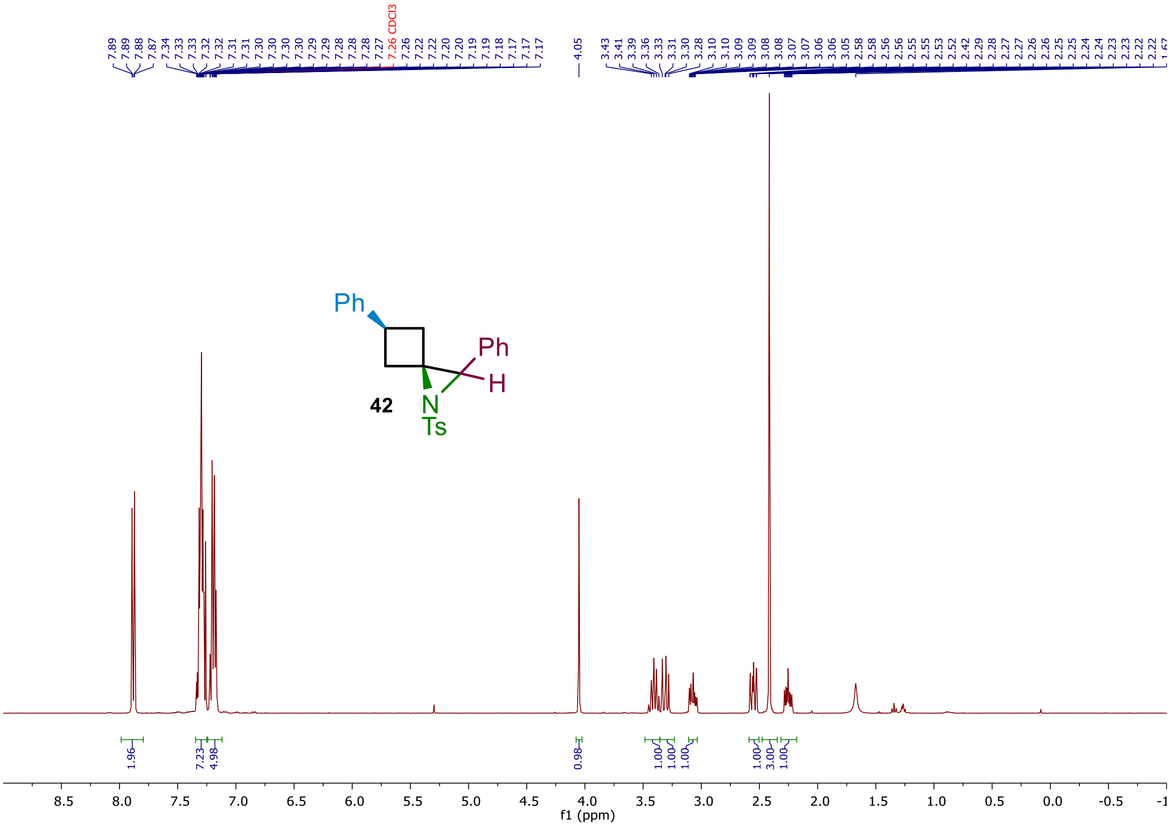

^{13}C NMR (101 MHz, $CDCl_3$) of **42**

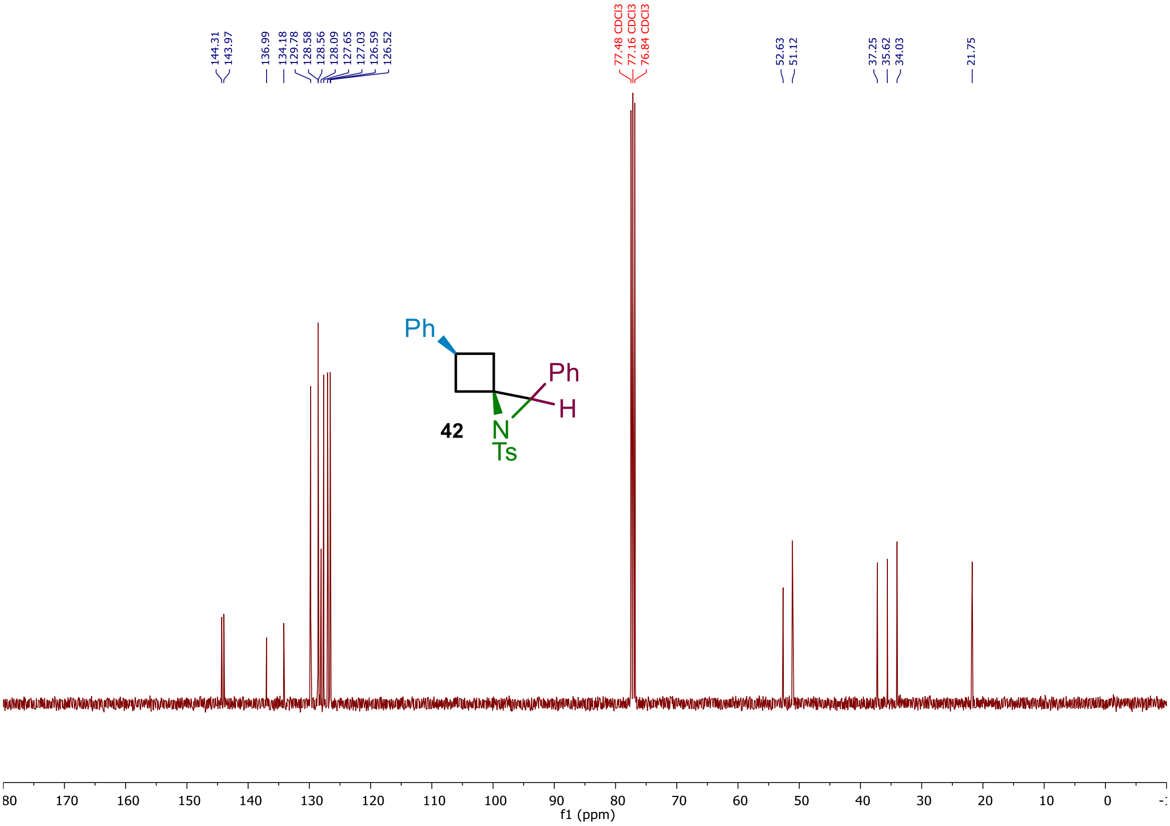

^{1}H NMR (400 MHz, $CDCl_3$) of **43** (*see procedure*)

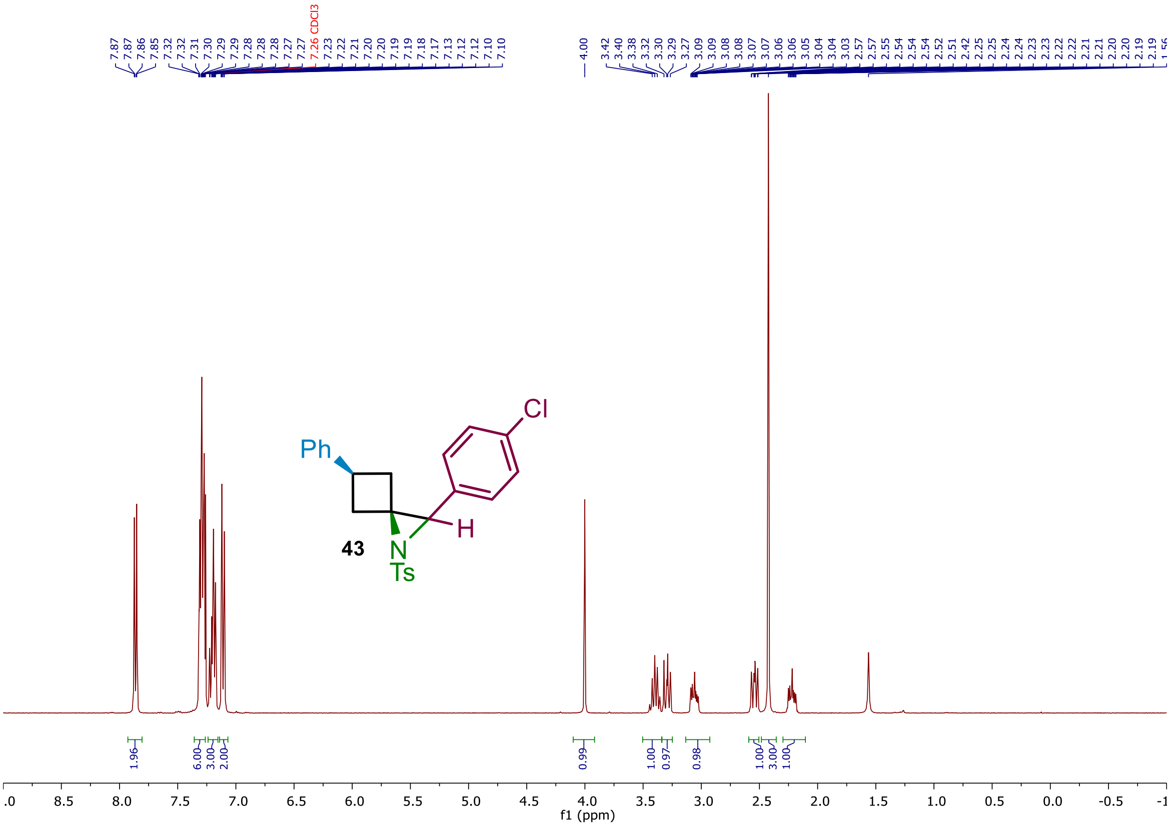

^{13}C NMR (101 MHz, $CDCl_3$) of **43**

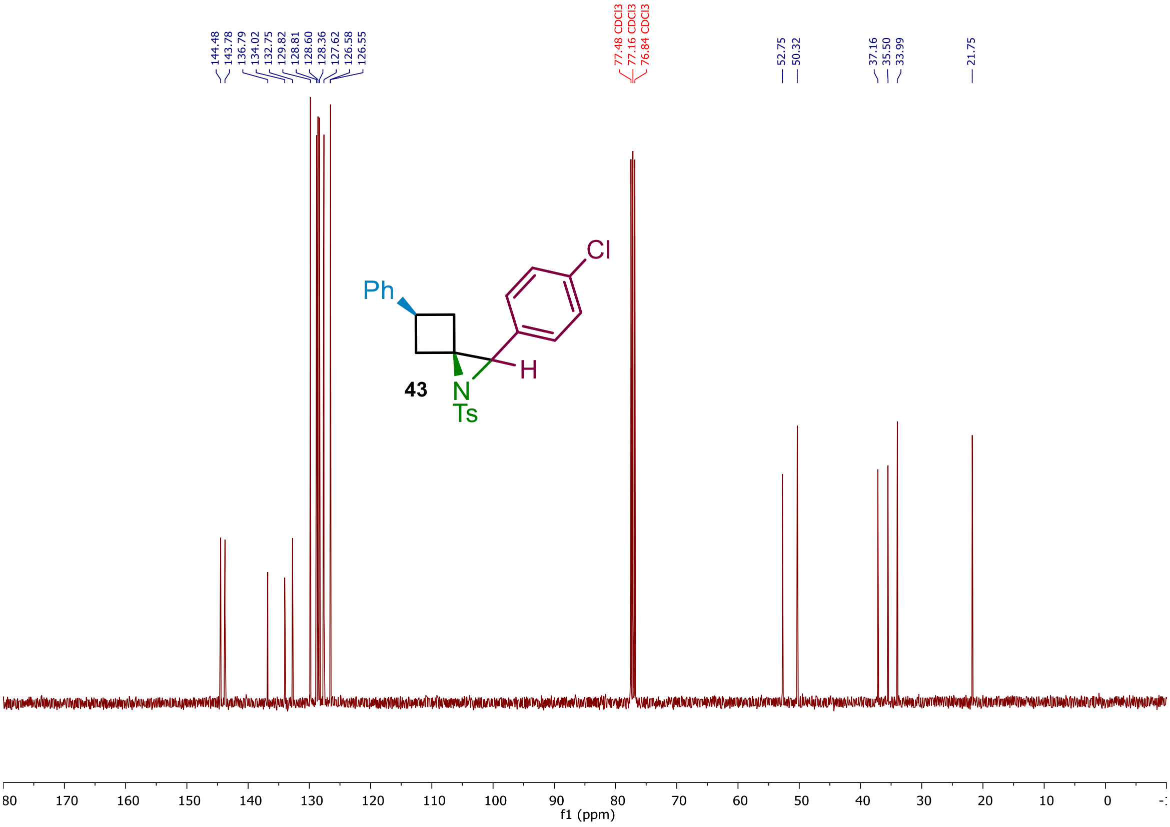

^{1}H NMR (400 MHz, $CDCl_3$) of **44** (*see procedure*)

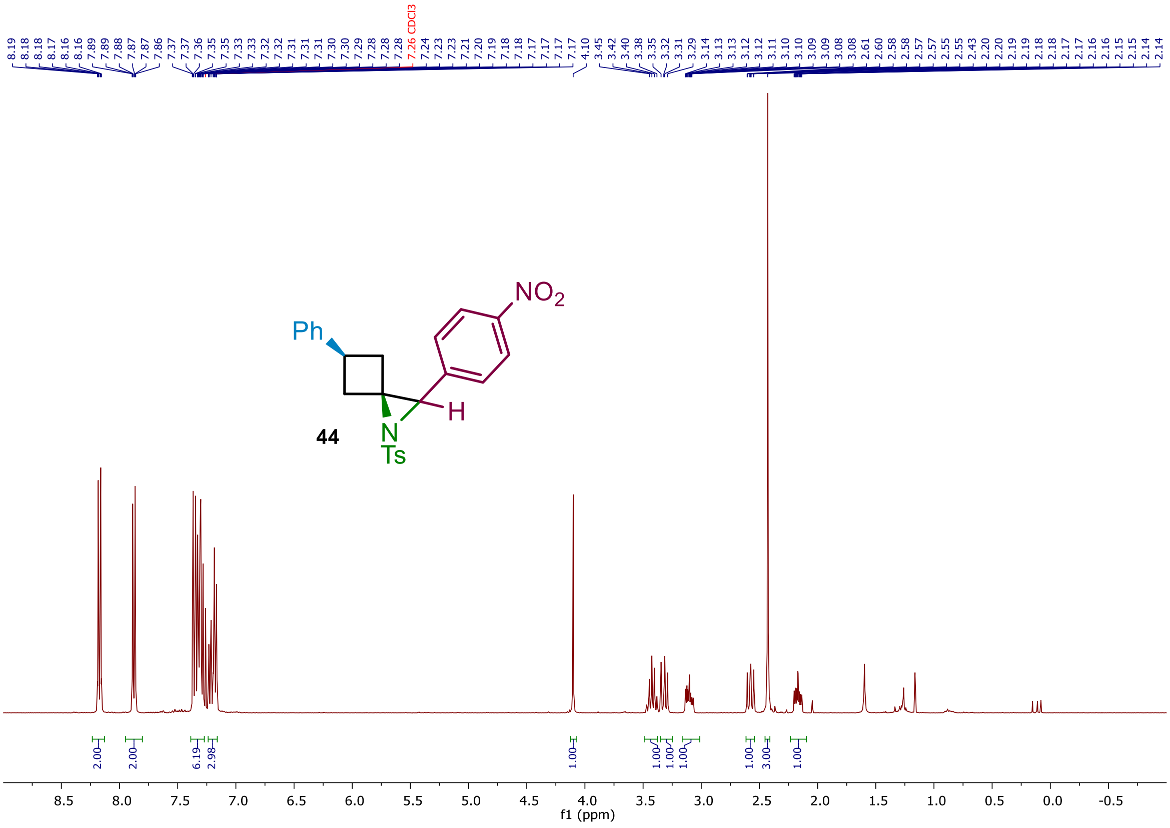

^{13}C NMR (101 MHz, $CDCl_3$) of **44**

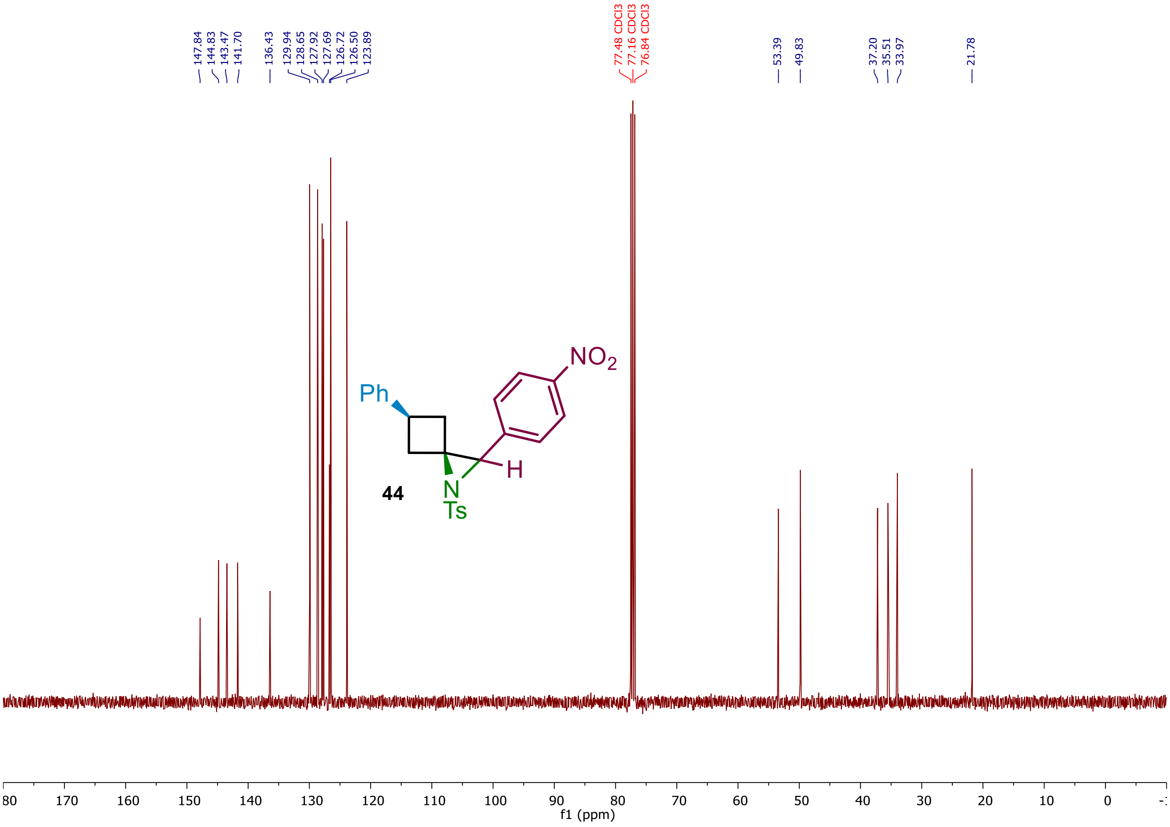

^{1}H NMR (400 MHz, $CDCl_3$) of **45** (*see procedure*)

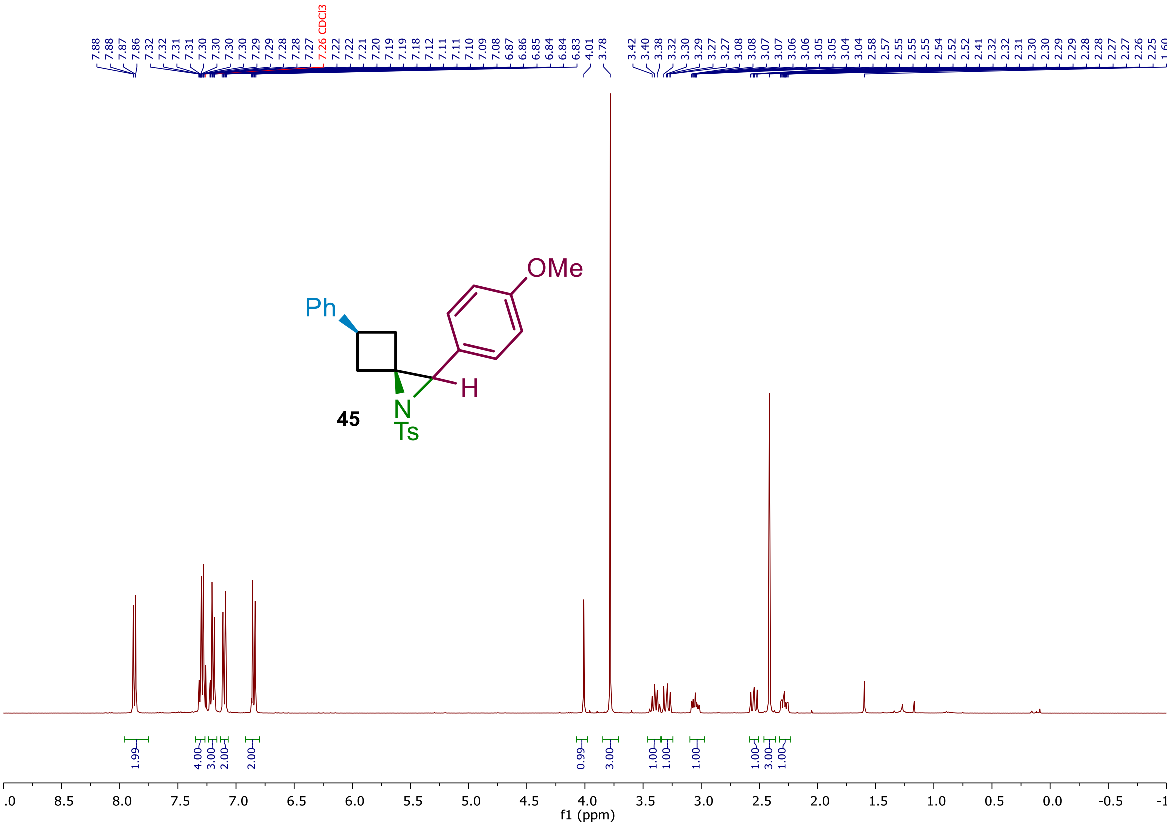

^{13}C NMR (101 MHz, $CDCl_3$) of **45**

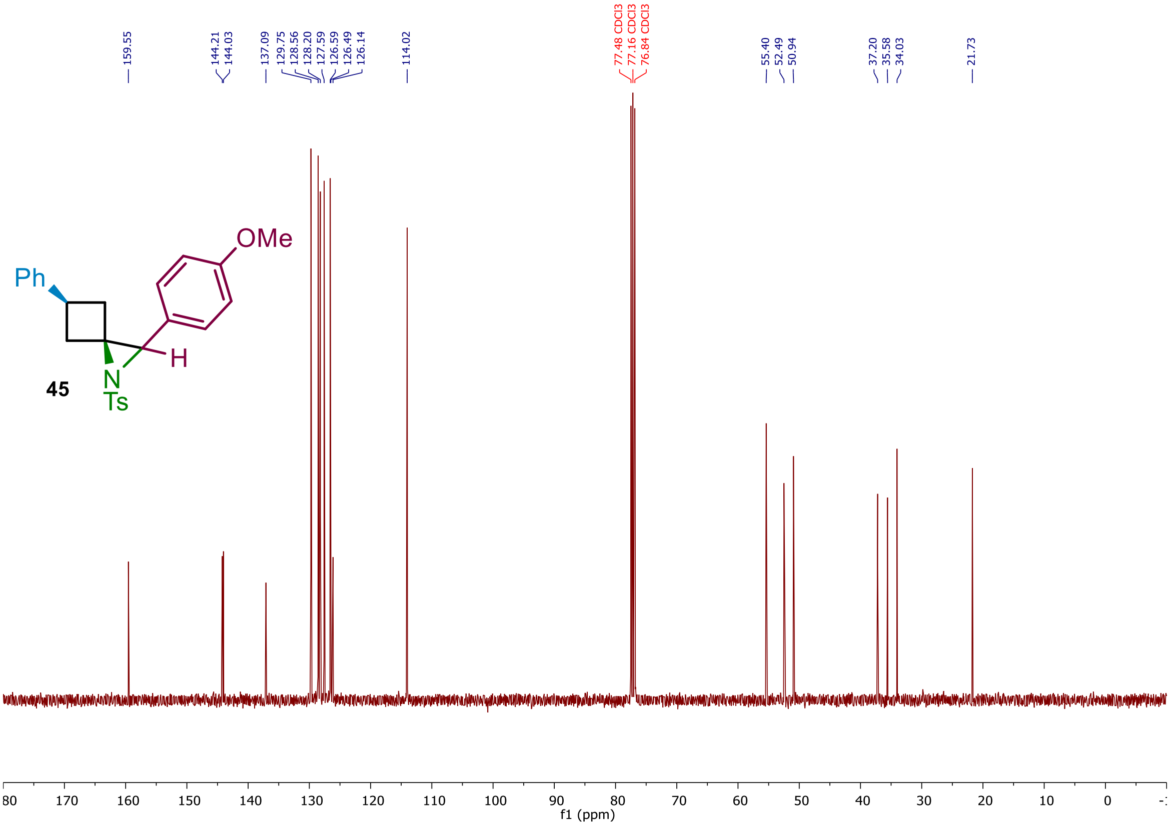

^{1}H NMR (400 MHz, $CDCl_3$) of **46** (*see procedure*)

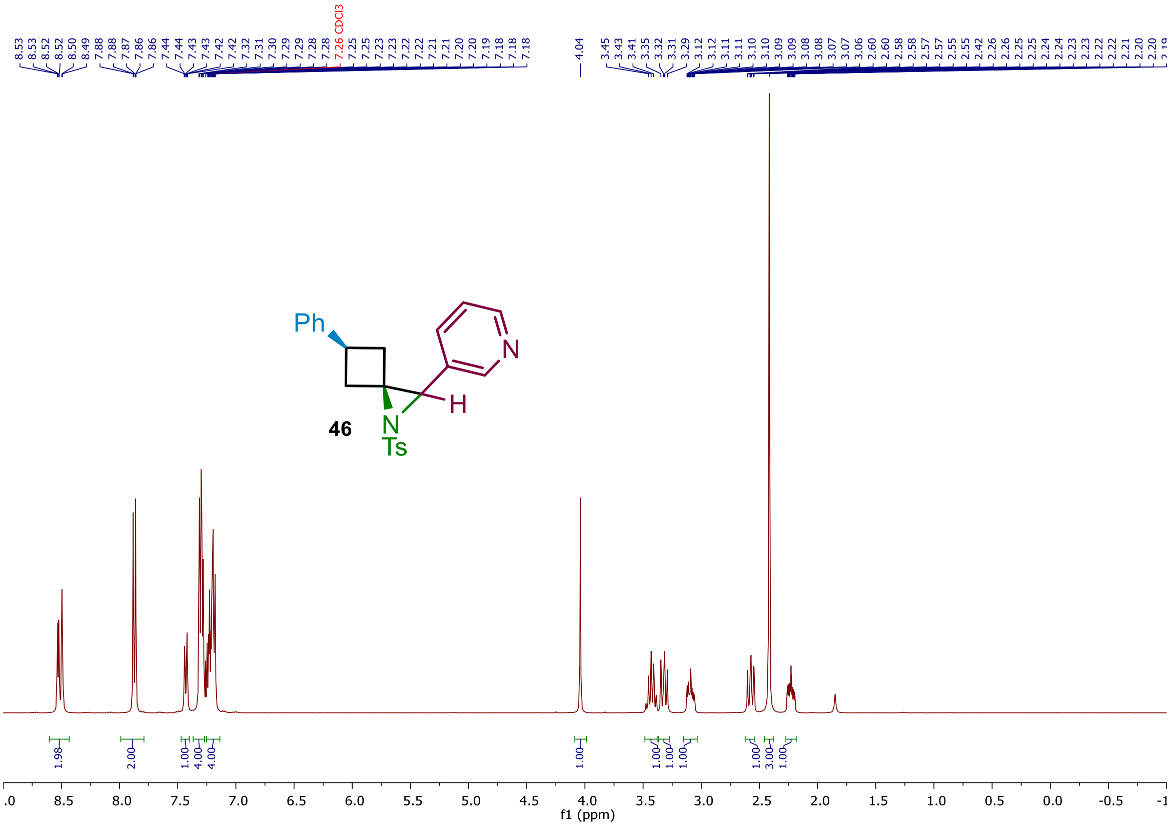

^{13}C NMR (101 MHz, $CDCl_3$) of **46**

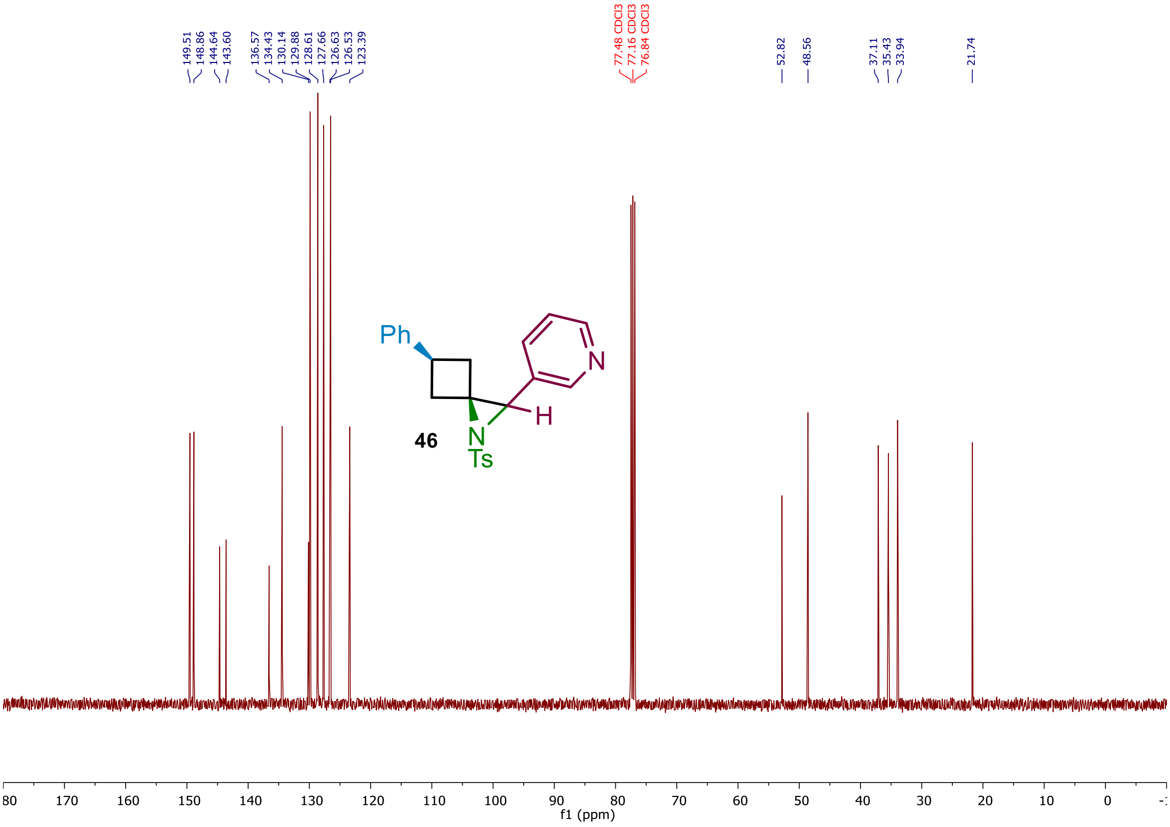

^{1}H NMR (400 MHz, $CDCl_3$) of **47** (*see procedure*)

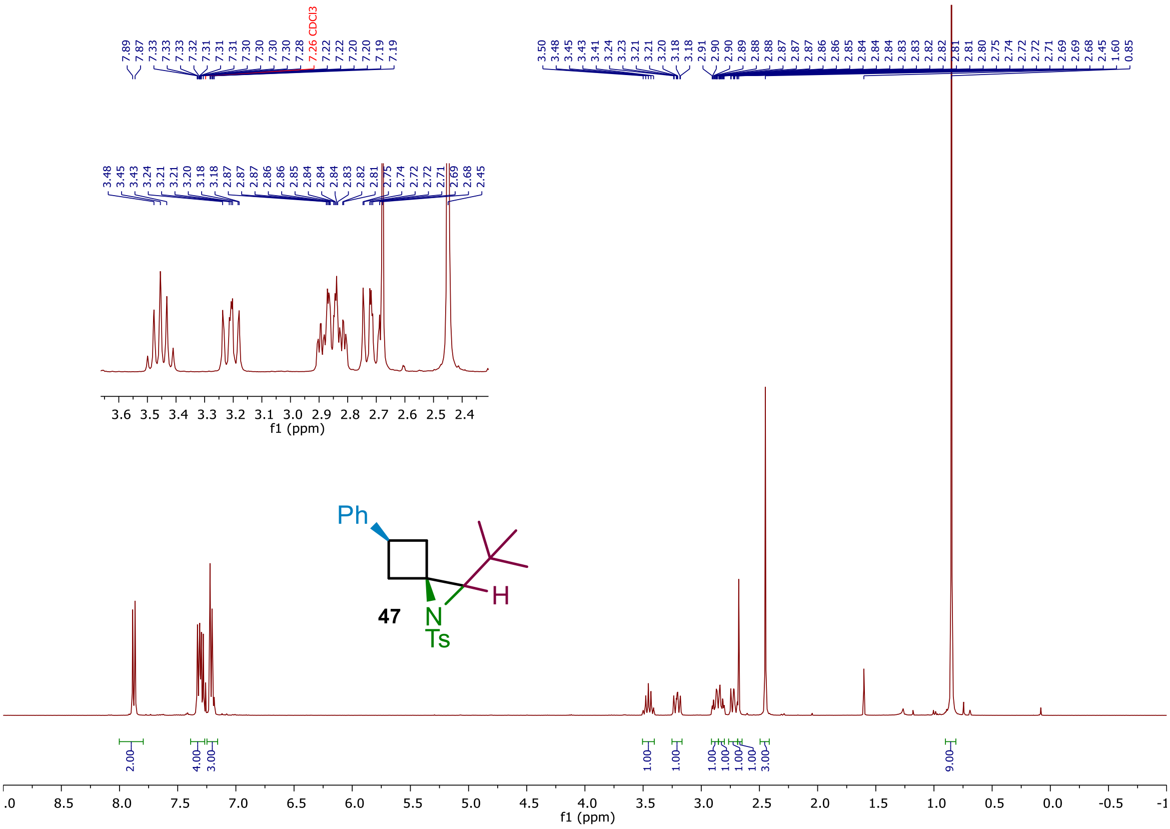

^{13}C NMR (101 MHz, $CDCl_3$) of **47**

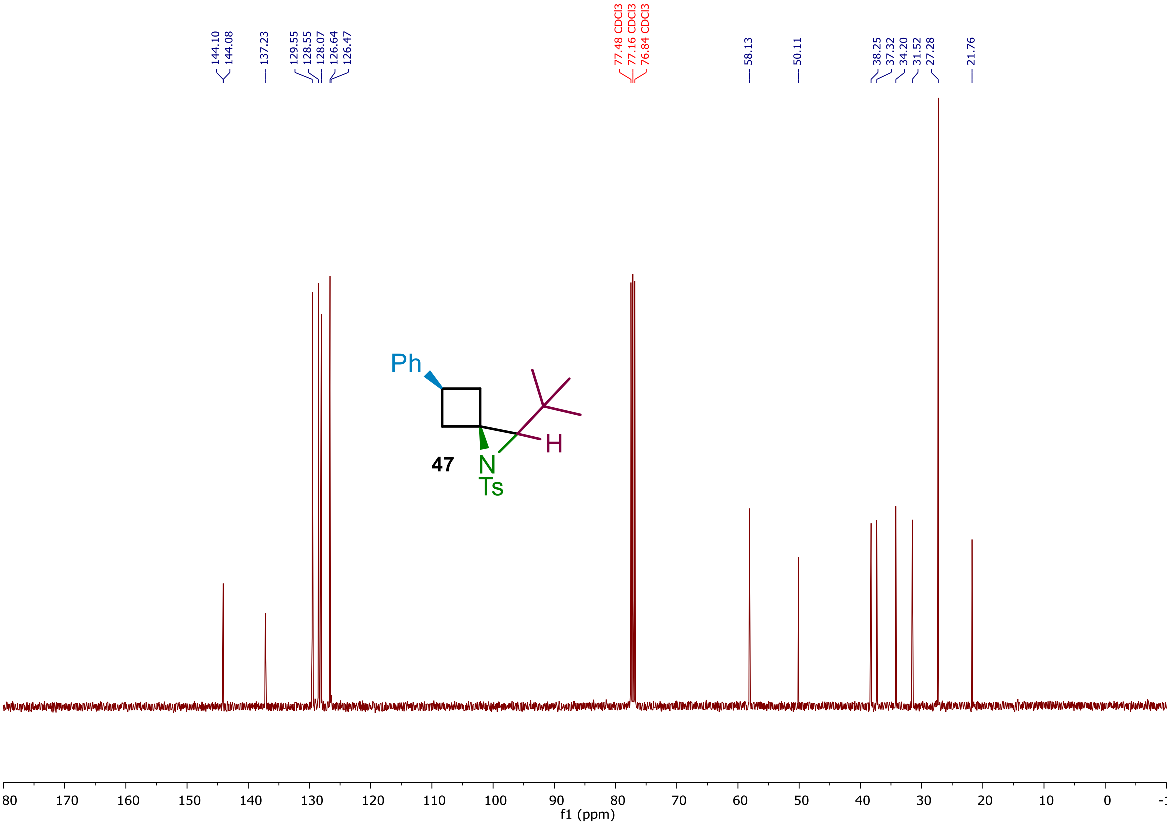

^{1}H NMR (400 MHz, $CDCl_3$) of **48** (*see procedure*)

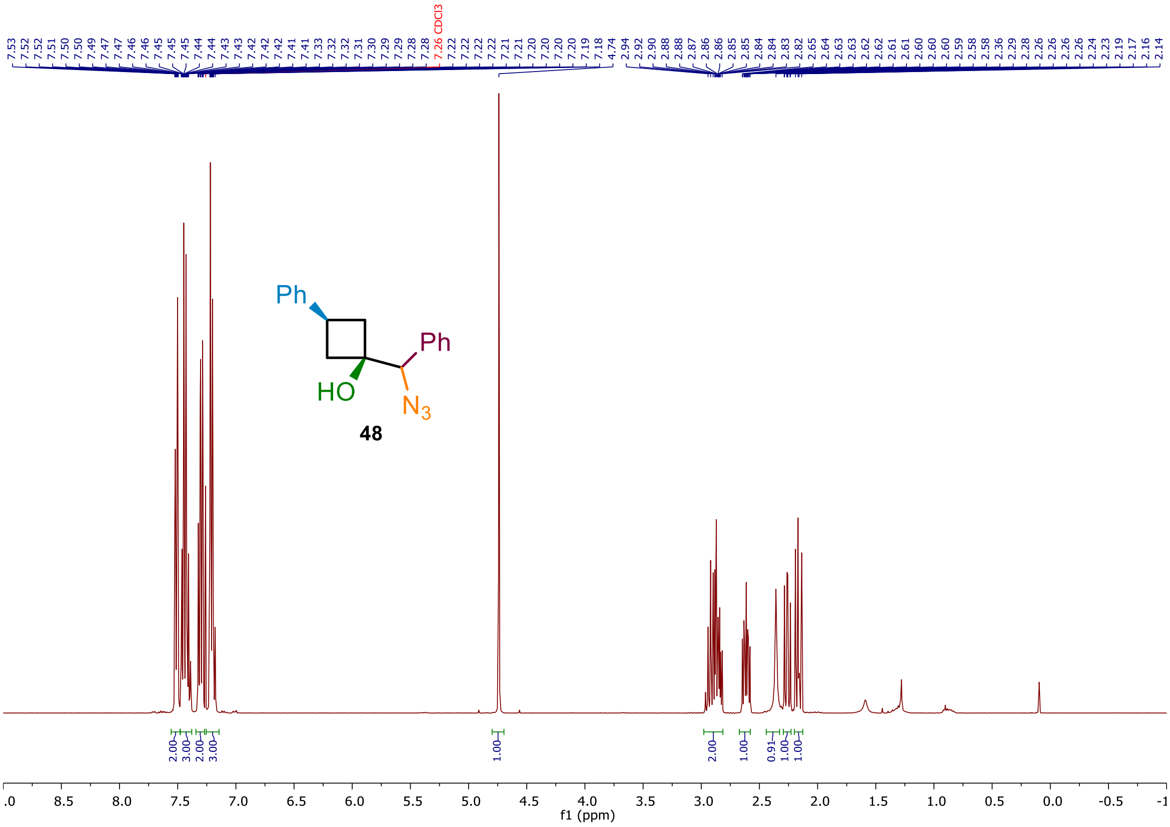

^{13}C NMR (101 MHz, $CDCl_3$) of **48**

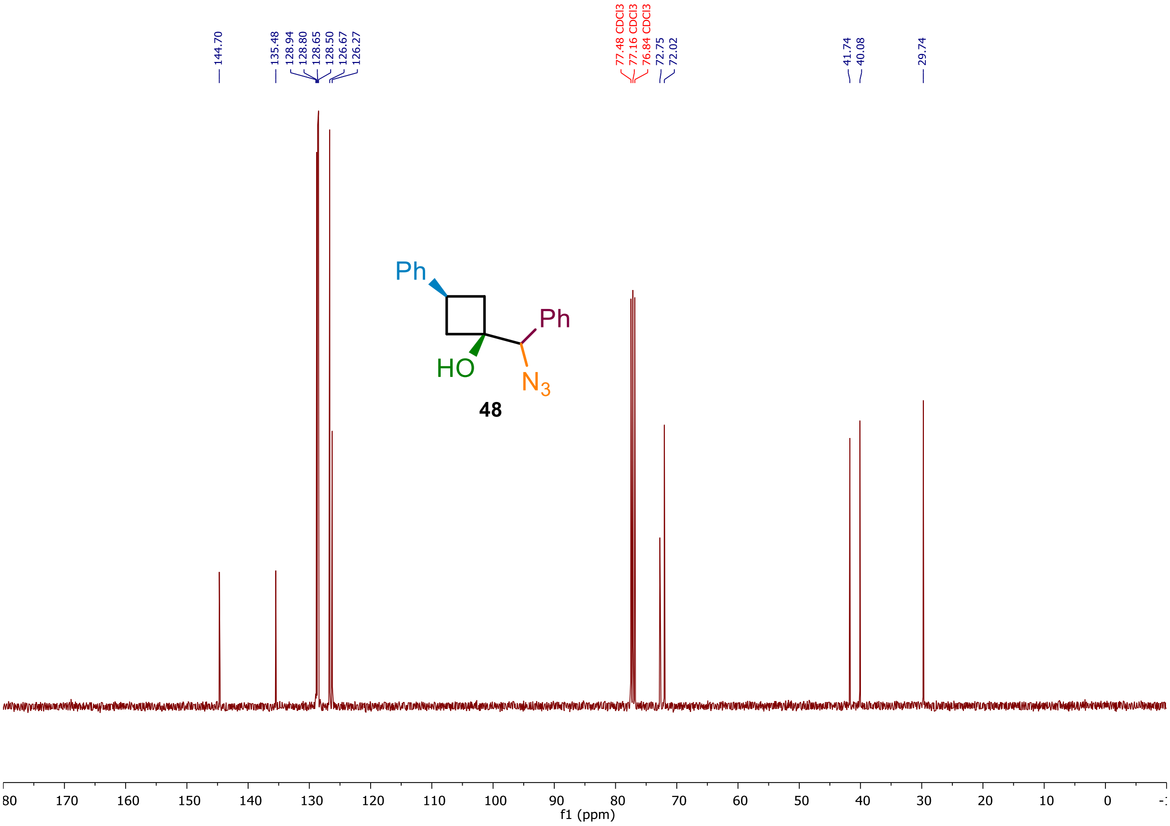

^{1}H NMR (400 MHz, $CDCl_3$) of **49** (*see procedure*)

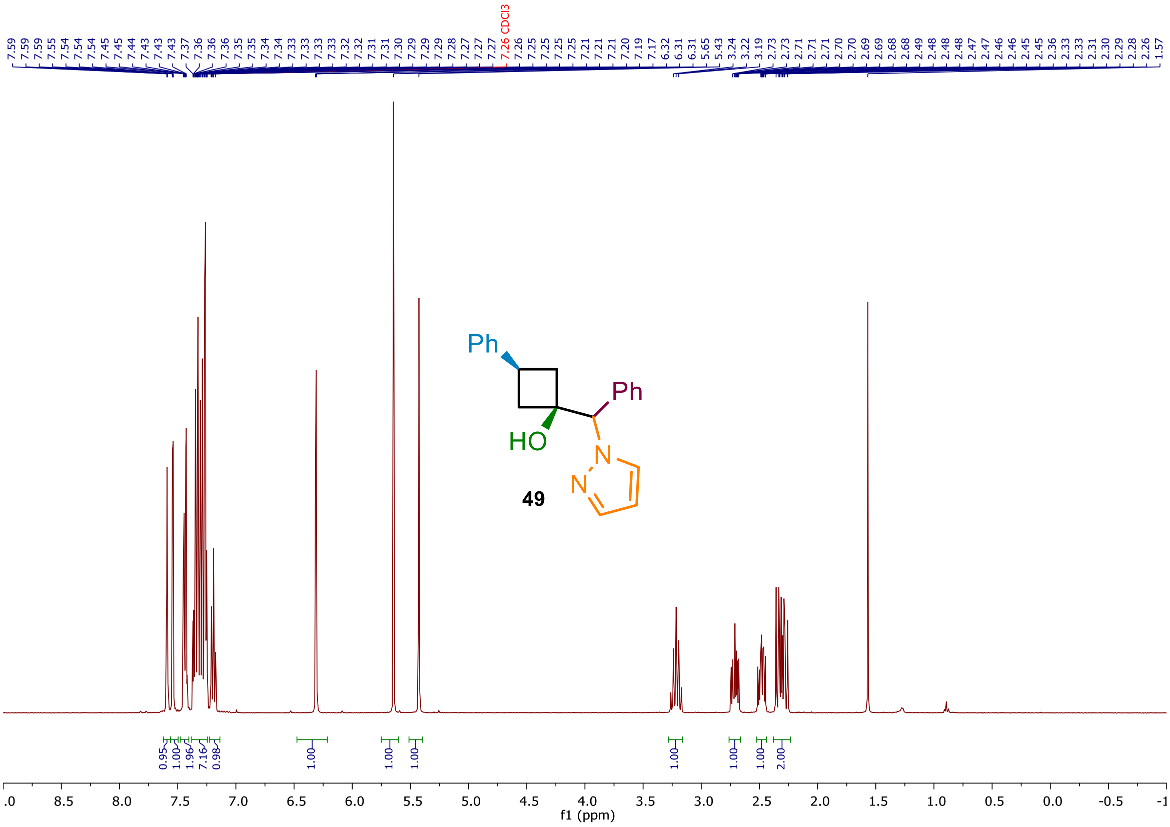

^{13}C NMR (101 MHz, $CDCl_3$) of **49**

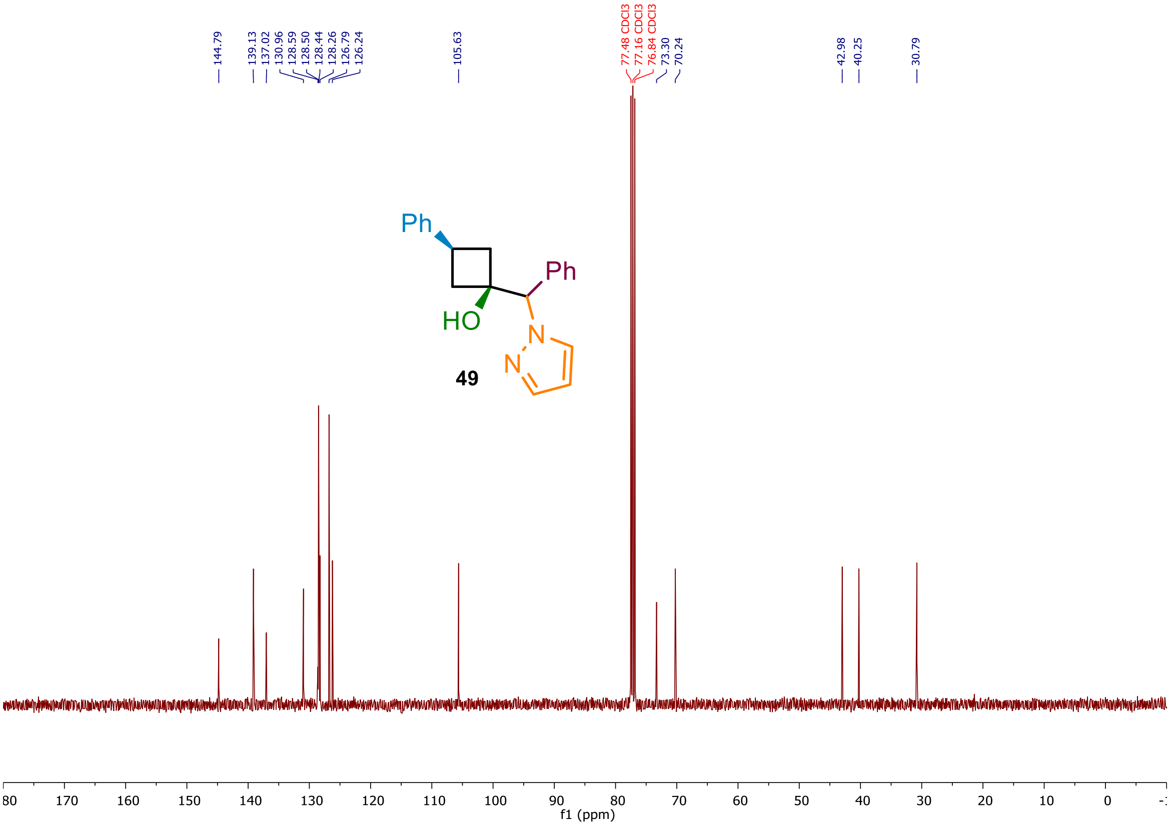

^{1}H NMR (400 MHz, $CDCl_3$) of **50** (*see procedure*)

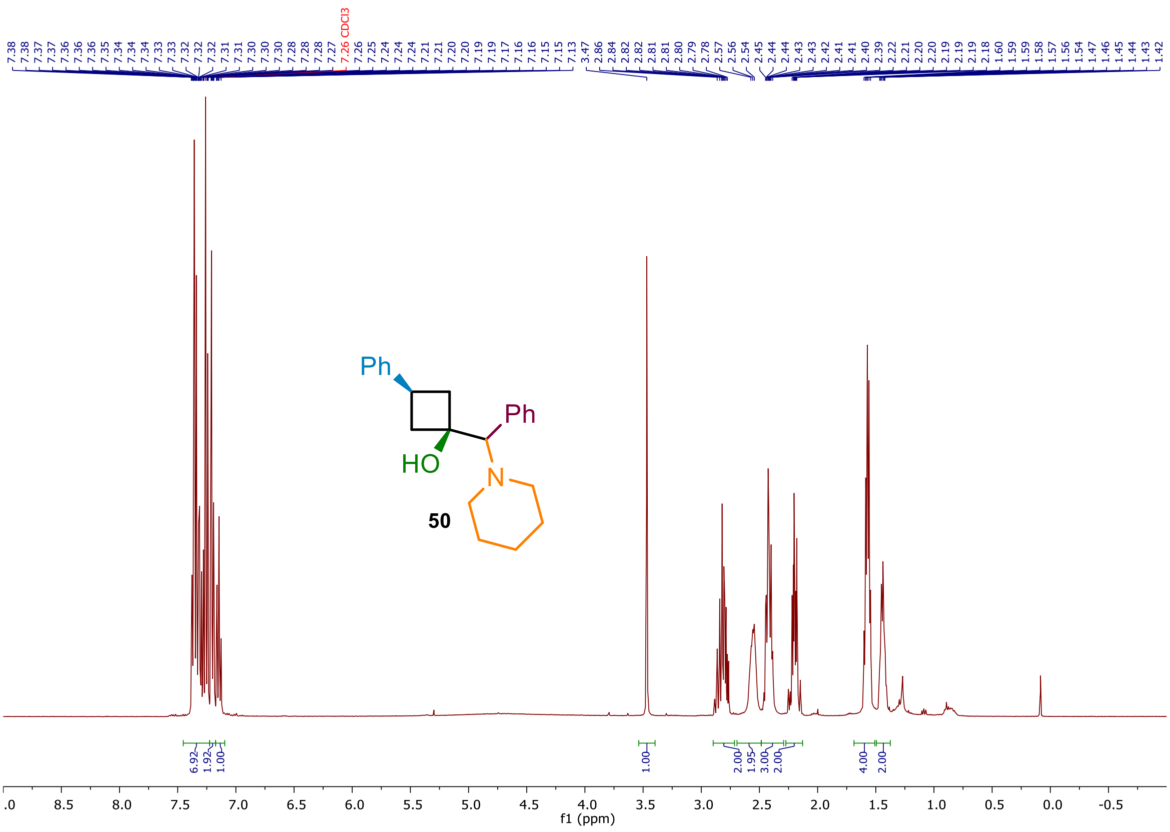

^{13}C NMR (101 MHz, $CDCl_3$) of **50**

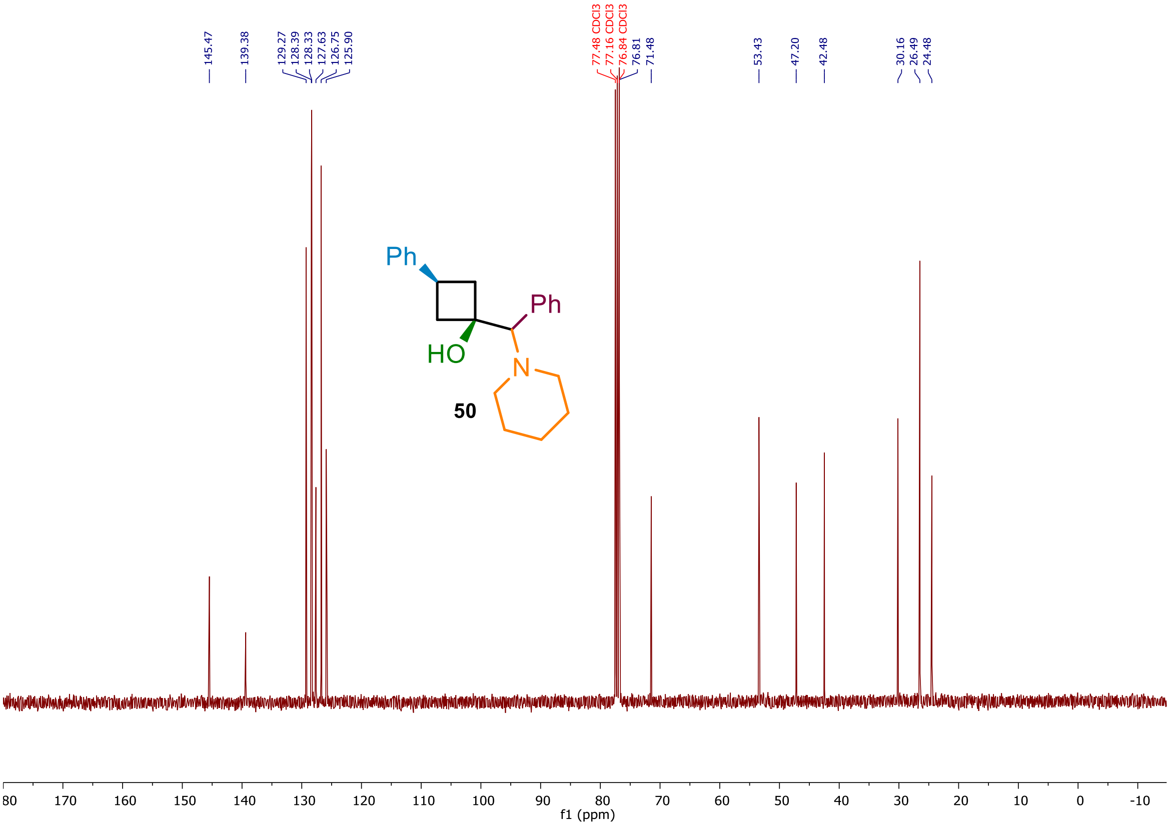

^{1}H NMR (400 MHz, $CDCl_3$) of **51** (*see procedure*)

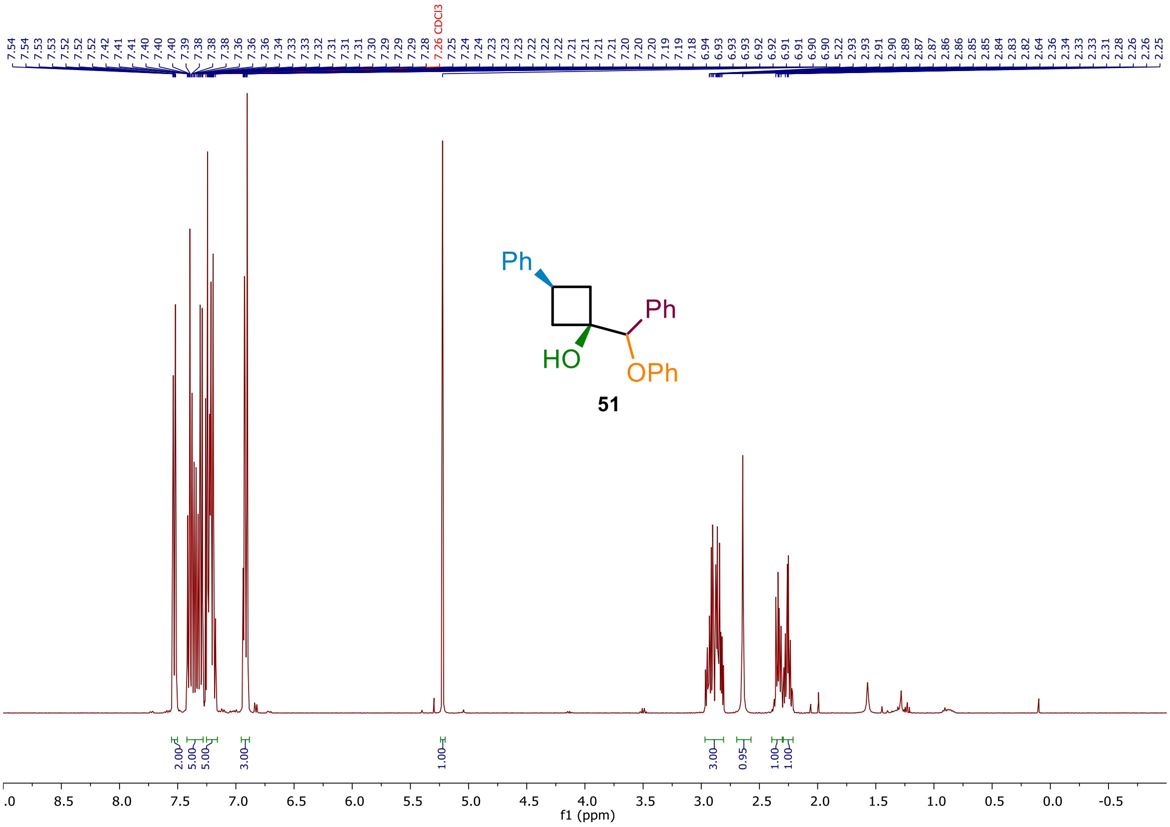

^{13}C NMR (101 MHz, $CDCl_3$) of **51**

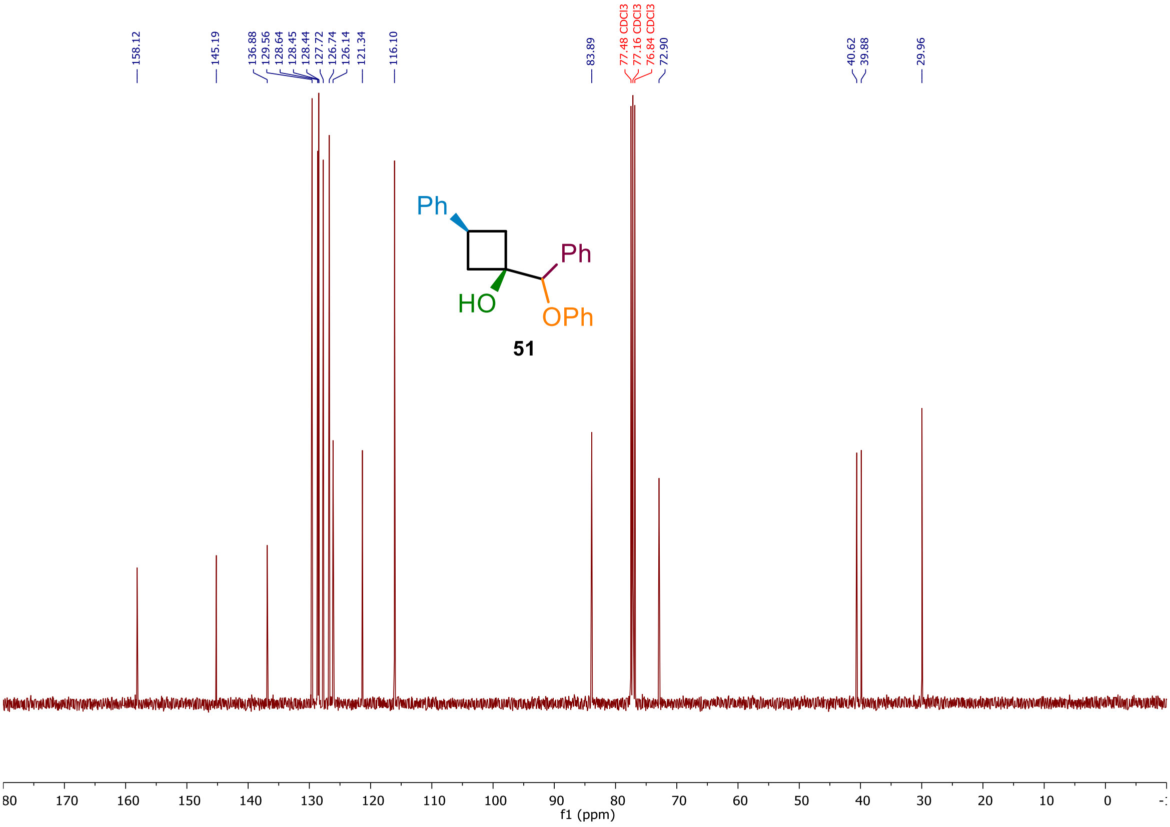

^{1}H NMR (400 MHz, $CDCl_3$) of **52** (*see procedure*)

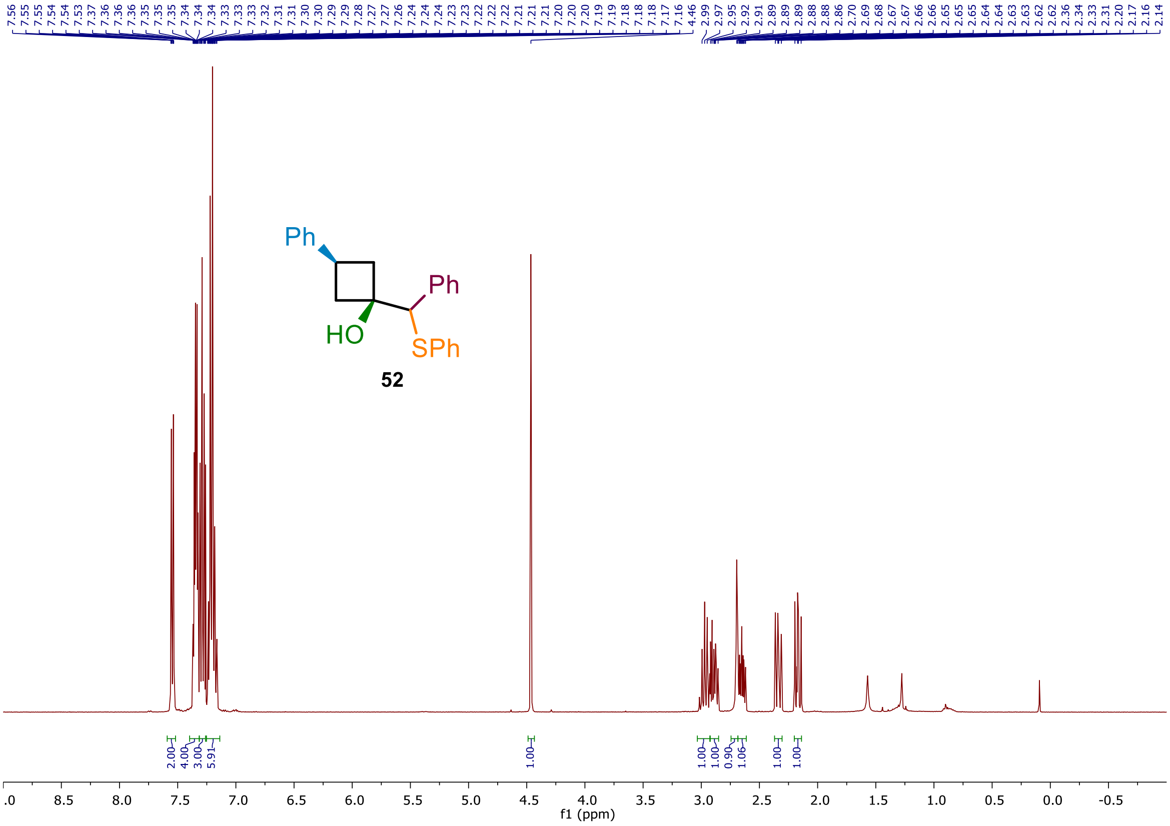

^{13}C NMR (101 MHz, $CDCl_3$) of **52**

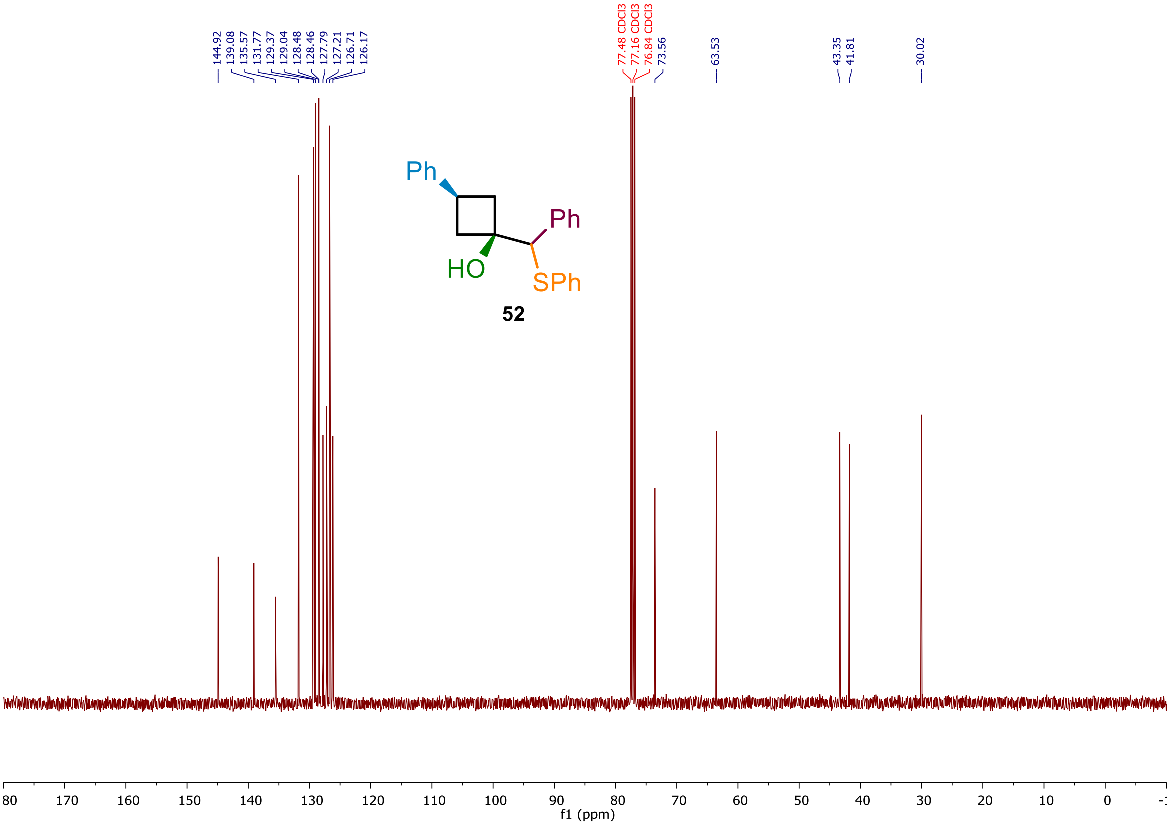

5. REFERENCES

1) A. F. Burchat, J. M. Chong, N. Nielsen, *J. Organomet. Chem.* **1997**, *542*, 281-283.
2) Y.-X. Liao, C.-H. Xing, Q.-S. Hu, *Org. Lett.* **2012**, *14*, 1544-1547.
3) X. Wang, Y. Tang, S. Ye, J. Zhang, Y. Kuang, J. Wu, *Org. Lett.* **2022**, *24*, 2059-2063.
4) M. Silvi, V. K. Aggarwal, *J. Am. Chem. Soc.* **2019**, *141*, 9511-9515.
5) Y. Zou, L. Qin, X. Ren, Y. Lu, Y. Li, J. Zhou, *Chem. Eur. J.* **2013**, *19*, 3504-3511.
6) D. Wang, C. Mück-Lichtenfeld, A. Studer, *J. Am. Chem. Soc.* **2019**, *141*, 14126-14130.
7) Z. Lei, A. Banerjee, E. Kusevska, E. Rizzo, P. Liu, M.-Y. Ngai, *Angew. Chem. Int. Ed.* **2019**, *58*, 7318-7323; *Angew. Chem.* **2019**, *131*, 7396–7401.
8) S. Morales, F. G. Guijarro, J. L. García Ruano, M. B. Cid, *J. Am. Chem. Soc.* **2014**, *136*, 1082-1089.
9) F. Sun, T. Yin, Y. Wang, A. Feng, L. Yang, W. Wu, C. Yu, T. Li, D. Wei, C. Yao, *Organic Chemistry Frontiers* **2020**, *7*, 578-583.
   For reference spectrum see: M. T. Bilodeau, A. M. Cunningham, *J. Org. Chem.* **1998**, *63*, 2800-2801.
10) A. Solladié-Cavallo, M. Roje, R. Welter, V. Šunjić, *J. Org. Chem.* **2004**, *69*, 1409-1412.
11) Z. Zhao, P. R. Bagdi, S. Yang, J. Liu, W. Xu, X. Fang, *Org. Lett.* **2019**, *21*, 5491-5494.
12) J. J. Acton, III, J. Bao, Q. Deng, M. Egbertson, R. Ferguson, III, X. Gao, S. T. Harrison, T. J. Henderson, S. L. Knowles, M. M.-C. Lo, R. D. Mazzola, Jr., M. Na, O. B. Selyutin, T. Suzuki, F. Zhang (Merck Sharp & Dohme Corp., MSD R&D (China) Co., Ltd.), WO2019005588, **2019**.
13) Q. Yang, M. Sheng, Y. Huang, *Org. Process Res. Dev.* **2020**, *24*, 1586-1601.
14) C. Lee, T. Jang, D. Choi, M. Ko, D. Kim, S. Kim, J. Min, W. Kim, Y. Lim (Chong Kun Dang Pharmaceutical Corp.), WO2013187646, **2013**.
15) M. Hannaby, S. Warren, *J. Chem. Soc., Perkin Trans. 1* **1989**, 303-311.
16) a) for **26** Bruker, *SAINT+ v8.38A Integration Engine, Data Reduction Software*, *Bruker Analytical X-ray Instruments Inc.*, *Madison, WI, USA*, 2015; b) for **43** Bruker, *SAINT+ v8.39.0 Integration Engine, Data Reduction Software*, *Bruker Analytical X-ray Instruments Inc.*, *Madison, WI, USA*, 2018.
17) a) for **26** Bruker, *SADABS 2014/5, Bruker AXS area detector scaling and absorption correction*, *Bruker Analytical X-ray Instruments Inc.*, *Madison, Wisconsin, USA*, 2014/5; b) for **43** Bruker, *SADABS 2018, Bruker AXS area detector scaling and absorption correction*, *Bruker Analytical X-ray Instruments Inc.*, *Madison, Wisconsin, USA*, 2018.
18) G. M. Sheldrick, *Acta Crystallographica a-Foundation and Advances* **2015**, *71*, 3-8.
19) a) G. M. Sheldrick, *Acta Crystallogr., Sect. A: Found. Crystallogr.* **2008**, *64*, 112-122; b) G. M. Sheldrick, *Acta Crystallogr. C* **2015**, *71*, 3-8.
20) O. V. Dolomanov, L. J. Bourhis, R. J. Gildea, J. A. K. Howard, H. Puschmann, *J. Appl. Crystallogr.* **2009**, *42*, 339-341.
